# Prognosis and Characterization of Microenvironment in Cervical Cancer Influenced by Fatty Acid Metabolism-Related Genes

**DOI:** 10.1155/2023/6851036

**Published:** 2023-03-08

**Authors:** Yanjun Zhou, Jiahao Zhu, Mengxuan Gu, Ke Gu

**Affiliations:** ^1^Department of Radiotherapy and Oncology, Affiliated Hospital of Jiangnan University, Wuxi, Jiangsu 214000, China; ^2^Department of Outpatient Chemotherapy, Harbin Medical University Cancer Hospital, Harbin, Heilongjiang 150000, China; ^3^Jiangnan University, Wuxi, Jiangsu 214000, China

## Abstract

Increasing evidence suggests that diverse activation patterns of metabolic signalling pathways may lead to molecular diversity of cervical cancer (CC). But rare research focuses on the alternation of fatty acid metabolism (FAM) in CC. Therefore, we constructed and compared models based on the expression of FAM-related genes from the Cancer Genome Atlas by different machine learning algorithms. The most reliable model was built with 14 significant genes by LASSO-Cox regression, and the CC cohort was divided into low-/high-risk groups by the median of risk score. Then, a feasible nomogram was established and validated by *C*-index, calibration curve, net benefit, and decision curve analysis. Furthermore, the hub genes among differential expression genes were identified and the post-transcriptional and translational regulation networks were characterized. Moreover, the somatic mutation and copy number variation landscapes were depicted. Importantly, the specific mutation drivers and signatures of the FAM phenotypes were excavated. As a result, the high-risk samples were featured by activated de novo fatty acid synthesis, epithelial to mesenchymal transition, angiogenesis, and chronic inflammation response, which might be caused by mutations of oncogenic driver genes in RTK/RAS, PI3K, and NOTCH signalling pathways. Besides the hyperactivity of cytidine deaminase and deficiency of mismatch repair, the mutations of POLE might be partially responsible for the mutations in the high-risk group. Next, the antigenome including the neoantigen and cancer germline antigens was estimated. The decreasing expression of a series of cancer germline antigens was identified to be related to reduction of CD8 T cell infiltration in the high-risk group. Then, the comprehensive evaluation of connotations between the tumour microenvironment and FAM phenotypes demonstrated that the increasing risk score was related to the suppressive immune microenvironment. Finally, the prediction of therapy targets revealed that the patients with high risk might be sensitive to the RAF inhibitor AZ628. Our findings provide a novel insight for personalized treatment in CC.

## 1. Introduction

Even with the implementation of HPV vaccination and screening programs, cervical cancer (CC) remains a major public health problem among women in high-development index countries and poverty areas [1]. CC patients often progress into the advanced stage, and recurrence leads to a poor prognosis [2]. How to identify the CC patients with high risk at the time of diagnosis still needs to be addressed in clinical practice.

Fatty acids (FAs) serve as important components of the membrane structure, secondary messengers, and fuels of energy production in cells [3]. To keep rapid and uncontrolled growth, the cancer cells consume a huge amount of nutrients, such as FAs and glucose, while excreting wastes which lead to a nutrient-deficient, acidic, and hypoxic tumour microenvironment (TME) [4]. Such hostile TME impairs the normal metabolic requirements of other intertumoural cells [5]. Among the genetically driven metabolic reprogramming of cancer cells, the FA metabolism (FAM) has been demonstrated to influence the growth and metastasis of tumour cells and modulate the recruitment and differentiation of tumour infiltrating cells in the TME [6]. Memory T cells fail to develop without FAs in culture [7]. Dendritic cells accumulating large amounts of lipids have been found to lose their antigen-presenting function in a variety of cancers [8]. Cancer-associated fibroblasts in the TME enhance FAO to boost colorectal cancer metastasis resulting in a poor prognosis [9]. The enhanced lipolysis and de novo FA synthesis also lead to lymphangiogenesis with endothelial cells [10]. However, rare research studies focus on the correlation between the prognosis of CC and FAM, which needs to be elucidated. Furthermore, the FAM can be regulated by oncogenic signalling pathway directly, namely, growth-factor receptor tyrosine kinases (RTKs)/RAS [11], phosphoinositide 3-kinase/protein kinase B (PI3K/AKT), and the mitogen-activated protein kinase (MAPK) signalling pathway [12]. However, the mechanisms driving particular FAM phenotypes in CC are still unclear. Moreover, increasing pieces of evidences indicate that the FAs as pivotal mediators can rewrite the TME and enhance cancer immune evasion and spread [5]. How FAM phenotypes affect the infiltration of immune and stromal cells is also unknown in the TME of CC.

To clarify the questions mentioned above, our study aims to construct a reliable and feasible prognostic model according to the FAM to stratify the CC patients. Furthermore, the specific mutation drivers of the FAM phenotypes and the connotations between the TME landscape and FAM phenotypes were evaluated systematically. Finally, due to the essential role of FAM reprogramming in cancer progression, potential therapy targets were predicted in CC patients which may provide a novel insight for personalized treatment.

## 2. Materials and Methods

### 2.1. Data Resource and Collection of FAM-Associated Genes

The transcriptome data and clinicopathologic information were downloaded from the Cancer Genome Atlas (TCGA) database (https://tcga-data.nci.nih.gov/tcga/ and https://portal.gdc.cancer.gov/), and mRNA expression was extracted from TCGA RNA-seq data for 306 CCs and 3 surrounding non-cancer tissues [13]. The genes were annotated by gencode.gene.info.v22. After removing patients without detailed clinicopathologic and overall survival (OS) information, we obtained 274 patients with CC in TCGA. The expression profiles of GSE44001 [14], which contained 300 early CC cases with disease-free survival (DFS) information, were obtained from the GEO (https://www.ncbi.nlm.nih.gov/geo/) database. The intersection was made from the genes related with FAM from GeneCards (https://www.genecards.org) and gene sets concerning FAM from the Molecular Signature Database (MSigDB) v7.4. Finally, 309 FAMs were selected (Supplementary Table 1).

### 2.2. Construction and Validation of FAM-Relevant Prognostic Signature

Randomly drawn 55% of samples (151 samples) were used for model training, and the remaining 45% (123 samples) were used for validation in the following analysis. The least absolute shrinkage and selection operator (LASSO) Cox regression analysis was employed in the training set to build a prognostic model for OS, using the R package “glmnet” [15]. LASSO-Cox regression analysis primarily selected useful predictive features to reduce the model complexity and multicollinearity and avoided overfitting to some extent. The proportional hazards (PH) assumption was conducted on the FAM-relevant prognostic genes by the R package “survival” and “survminer.” According to the prognostic model, the risk score was exported for each CC patient:

(1) $$\begin{array}{c} risk\;score\left(RS\right)=\overset{n}{\underset{i}{\sum }}\left(Expi*Coefi\right)\text{.} \end{array}$$

Expi means the expression level of each FAM gene, and Coefi stands for the corresponding regression coefficient. To make the prognostic model as concise as possible, the patients were divided into high-risk and low-risk groups by the median of RS. The risk curve was plotted according to the RS and risk group, and the survival status and RS were evaluated according to the curve.

To evaluate the feasibility of the prognostic model in predicting survival in CC patients, we conducted a Kaplan–Meier analysis of overall survival (OS) by the R package “survival,” operating characteristic curve (ROC), and area under the curve (AUC) by the means of the R package “timeROC” in both the training dataset and testing dataset. Kaplan–Meier survival curves were plotted and *p* values were calculated using the log-rank test to explore the survival difference between risk groups [16]. The AUC ranges from 0 to 1. When AUC lies between 0.5 and 0.6, 0.6 and 0.7, or is >0.7, the performance of the model is considered poor, fair, or good, respectively.

### 2.3. Establishment and Validation of a Nomogram

The PH assumption was conducted on the RS and risk grouping by the R package “survival” and “survminer.” To compare the predictive value of risk grouping or RS in survival analysis with traditional clinical-pathologic parameters, the univariate Cox regression and multivariate Cox regression were employed to calculate hazard ratios (HRs) and 95% confidence intervals (CIs) by the R package “survival” and “survminer.” To further improve the predictive accuracy of our FAM gene signature by combining it with other clinical-pathologic features (e.g., body mass index (BMI), pathology type, pathology grade, and stage), we built an easy-to-use and clinically adaptable risk nomogram for predicting the OS probability in CC patients using the “rms” package in R [17]. The OS probabilities were predicted for 1-, 2-, 3-, and 5-year survival.

The validation of the nomogram-based prediction model was accessed via bootstrapped calibration curves using “rms” in R and quantified as a Harrell's concordance index (*C*-index) by the function “rcorrcens” in R package “Hmisc.” *C*-index was utilized to evaluate the discriminative capabilities of the nomograms. Calibration curves (1000 bootstrap resamples) were generated to compare the consistency between the predicted and observed OS for 1, 2, 3, and 5 years [18]. The net reclassification index (NRI) was employed to evaluate the added value of new risk group or RS to existing prognostic models. Decision curve analysis (DCA) was applied to evaluate the impact on decision making in clinical practice of the nomograms using the “stdca” function in R [19].

### 2.4. Comparison of Models Built by Other Machine Learning Methods

Furthermore, to choose the best prognostic model, the support vector machine (SVM) and the random forest method were performed to classify the vital status in the CC cohort using “e1071” and “randomForest” packages in R. The 274 CC cohort was randomly divided into a training cohort and a testing cohort as described before. The Wilcoxon test assessed the performance of the SVM model and random forest model. The discriminatory power of the SVM model and random forest model on vital status was assessed by the AUC in training and testing datasets. To obtain the best SVM model, the AUC and prediction accuracy of the linear, polynomial, radial, and sigmoid models were compared. The variables were derived from the best polynomial model. Then, to further compare the discriminative ability between the FAM genes and our signature genes, principal component analysis (PCA) was carried out using the “pca” function.

### 2.5. Identification of Differential Expression Genes and Functional Enrichment Analysis

Differential expression genes (DEGs) for low/high-risk groups were calculated by the R “limma” package. The threshold (adjusted *p* value <0.05 and |Log2 fold change (FC)| > 1.2) was used as a selection criterion for the DEGs. A volcano plot and a heat map of the DEGs were depictured. Gene ontology (GO) enrichment [20] and gene set enrichment analysis (GSEA) [21] were employed to decipher the enrichment of signalling transductions and biological functions in the DEGs in CC patients using the functions of gseGO and gseKEGG in “GSEA” package. The enrichments according to the MSigDB and Reactome were analysed. Then, gene set variation analysis (GSVA) was further carried out by the “GSVA” in R [22]. The gene sets of “h.hallmark.v7.4.symbols.gmt” (HALLMARK) and “c2.cp.kegg.v7.4.symbols.gmt” (KEGG) were used as the reference molecular signature databases, and adjusted *p* value <0.05 and |Log2 (FC)| > 0.1) were considered statistically significant.

### 2.6. Identification of Hub Genes and Regulation Network

To obtain the hub genes among the DEGs, the “GOSemSim” package in R was employed [23]. Meanwhile, the likelihood of protein-protein interactions (PPIs) among the DEGs was identified in our study from STRING database, which is based on either literature of direct interaction experiments or prediction from co-expression and gene arrangement in the genome. Moreover, the list of 318 transcription factors was acquired from https://www.cistrome.org/. The correlation between the DEGs and transcription factors was defined as the correlation coefficient (*R*) = 0.5, *p* value = 0.001. Additionally, the miRNAs that interact with the DEGs, validated by luciferase reporter assay, were obtained by R packages “multiMiR” and “mirtarbase” [24]. The long non-coding RNAs (lncRNAs) interacting with miRNAs were obtained from “starbase” [25]. The correlation among the DEGs, miRNAs, and lncRNAs was illustrated by “ggalluvial” package.

### 2.7. Somatic Genomic Alternation Analysis

To identify the gene mutation characteristics in CC patients, we analysed somatic mutation data by the R package “maftools” [26]. The summary oncoplots were based on MutSigCV algorithm by maftools. The mutation pattern of specific genes was represented by oncoplot function in maftools. Transitions and transversions were calculated using the titv function in maftools. The changes in the amino acid of a certain protein were depictured by the lollipopPlot in maftools. The tumour mutational burden (TMB) values were calculated in units of mutations per megabase (MB) and characterized as low (TMB < 6), intermediate (6 ≤ TMB < 20), or high (TMB ≥ 20) [27].

### 2.8. Identification of Mutation Driver and Affected Signalling Pathway

The function oncodrive in maftools [26] was employed to identify driver genes, based on OncodriveCLUST algorithm [28]. The OncogenicPathways function was used to check the enrichment of oncogenic signalling pathways. The effects of a specific gene mutation on OS were manifested by mafSurvival in maftools [26]. The comparison of the two risk groups to detect differentially mutated genes was achieved by mafCompare in maftools and then the result was visualized by forestPlot in maftools. The drug-gene interactions were checked by the drugInteractions in maftools.

### 2.9. De Novo Mutational Signature Analysis and APOBEC Enrichment Estimation

The signature analysis was performed by a series of functions in maftools. The mutational matrix was first decomposed into signatures by negative matrix factorization. The extracted signatures then were compared against the Catalogue of Somatic Mutations in Cancer (COSMIC) mutational signatures v2 and updated v3 [29]. The different mutation patterns between apolipoprotein B mRNA editing enzyme, catalytic polypeptide-like (APOBEC) enriched and non-APOBEC enriched samples were achieved by the function plotApobecDiff of maftools.

### 2.10. Copy Number Variation Analysis

Because the copy number variations (CNVs) can contribute to cancer susceptibility, we further detected the common CNV regions by the GenePattern website (https://cloud.genepattern.org/gp/pages/index.jsf) with corresponding GISTIC 2.0 module [30].

### 2.11. Identification of Neoantigens

We sought to explore neoantigen in the both groups of CC patients and responsiveness to therapies. The neoantigen data from the CC cohort were downloaded from https://biopharm.zju.edu.cn/tsnadb [31] and https://tcia.at/home [32]. The neoantigen burden of a certain patient was predicted bioinformatically, as following standards. The half maximal inhibitory concentration (IC_50_) < 500 nM was considered a predicted binder. Patient-specific neoantigens were defined as any unique combination of peptide sequence: human leukocyte antigen (HLA)-allele with mutant peptide-binding affinity IC_50_ < 500 nM, and corresponding wild-type peptide IC_50_ > 500 nM. Expressed neoantigens were defined as neoantigens with RNA-sequencing counts ≥1 [33].

### 2.12. Correlation between Cancer Germline Antigens and Immune Cell Infiltration

To chart the antigenome for each sample, we used RNA-sequencing data to derive expression levels of cancer germline antigens (CGAs). Due to the low tumoural specificity of CGAs, we used the CGA gene list retrieved from https://tcia.at [32]. The expression levels of CGA genes were compared between both of the groups, and we obtained 37 differentially expressed CGAs according to the risk levels. Furthermore, we explored the relationship between the 37 genes and CD8 T and regulatory T cell enrichment by the “corrplot” in R.

### 2.13. Evaluation of the Cellular Composition in Tumour Microenvironment

To provide a comprehensive view of the cellular composition of the intratumoural immune infiltrates, we carried out the immunogenomic characterization of the CC patients by the “IOBR” package [34], which includes 8 algorithms to estimate the immune infiltrating cells. To further identify the significantly enriched cells in the TME, the correlations between the RS and each type of infiltrating cells were calculated by “corrplot” in R and the importance of each infiltrating cells in survival was calculated as log10 transformed *p* value by Cox regression. The infiltrating cell types with a *p* value of correlation under 0.01 were selected and depictured. Potential implications for immunotherapy were calculated by the website https://tide.dfci.harvard.edu/ [35].

### 2.14. Exploration of Potential Therapeutic Drugs concerning Prognostic Models

To explore potential clinical drugs for the treatment of high-risk CC patients, we used the R package “pRRophetic” to predict the sensitivity to the compounds obtained from the Genomics of Drug Sensitivity in Cancer (GDSC) website according to the CC dataset in TCGA database [36].

### 2.15. Statistical Analysis

Continuous variables were compared by the Wilcox test, while categorized variables were compared by ANOVA. All the analyses were performed by R software (Version 4.1.3, the R foundation for statistical computing). *P* values lower than 0.05 were considered to be significant unless special instruction was given.

## 3. Results

### 3.1. Construction and Validation of FAM-Relevant Prognostic Model

**Figure 1:**
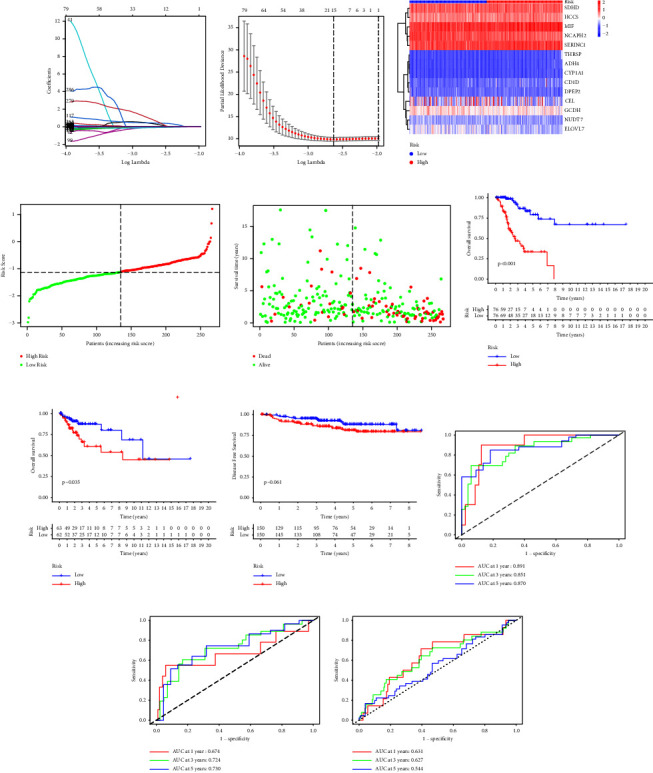
Construction and validation of FAM-relevant prognostic signature. Shrinkage of coefficients (a) and partial likelihood deviance (b) in LASSO-Cox regression analysis. (c) Expression levels of 14 significant genes in LASSO model. (d) The grouping of patients based on the median risk score. (e) The survival time of patients concerning the risk score. Kaplan–Meier (KM) analyses of OS based on risk group in training cohort (f) and testing cohort (g). KM analyses of DFS based on risk group in validation cohort GSE44001 (h). The receiver operating characteristic curve of the LASSO model in training cohort (i), testing cohort (j) and validation cohort (k). CD1D, CD1d molecule; CEL, carboxyl ester lipase; NCAPH2, non-SMC condensin II complex subunit H2; SDHD, succinate dehydrogenase complex subunit D; ADH4, alcohol dehydrogenase class II Pi chain; HCCS, holocytochrome C synthase; THRSP, thyroid hormone responsive; GCDH, glutaryl-CoA dehydrogenase; NUDT7, nudix hydrolase 7; DPEP2, dipeptidase 2; SERINC1, serine incorporator 1; MIF, macrophage migration inhibitory factor; ELOVL7, ELOVL fatty acid elongase 7; CYP1A1, cytochrome P450 family 1 subfamily A member 1.

We utilized the LASSO-penalized Cox regression to determine the LASSO tuning parameter *λ*, resulting in the minimum squared error. The results showed that when specific 14 genes were included in the prognostic model, the model contraction was stable, the partial likelihood deviance was minimal, and the optimal *λ* was 0.01961 (Figures 1(a) and 1(b)). Finally, 14-gene signature based on FAM, including CD1d molecule (CD1D), carboxyl ester lipase (CEL), non-SMC condensin II complex subunit H2 (NCAPH2), succinate dehydrogenase complex subunit D (SDHD), alcohol dehydrogenase class II Pi chain (ADH4), holocytochrome C synthase (HCCS), thyroid hormone-responsive (THRSP), glutaryl-CoA dehydrogenase (GCDH), nudix hydrolase 7 (NUDT7), dipeptidase 2 (DPEP2), serine incorporator 1 (SERINC1), macrophage migration inhibitory factor (MIF), ELOVL fatty acid elongase 7 (ELOVL7), and cytochrome P450 family 1 subfamily A member 1 (CYP1A1), was identified to construct the prognostic model. The coefficient of each gene is summarized in Supplementary Table 2. The results of the PH assumption of each gene are listed in Supplementary Table 3 and Supplementary Figures 3a–3n and 3q–3t. The CC cohort was divided into a low-risk group and a high-risk group by the median of RS (Figure 1(d)). The expression levels of these 14 genes were represented in the different risk groups (Figure 1(c)). To verify the utility of our prognostic model, the associations among vital status, time, and RS of each group were determined. With the increasing RS, the death events tended to increase in CC patients (Figure 1(e)). Furthermore, the results showed that the patients in the high-risk group had a worse OS than those in the low-risk group in both the training cohort and testing cohort (*p* < 0.001 and *p*=0.035, respectively, Figures 1(f) and 1(g)). Furthermore, ROC was employed to confirm the predictive value of the model. We observed that the AUC values were 0.891, 0.851, and 0.870 at 1 year, 3 years, and 5 years, respectively, which may suggest that the performance of the model was good at all three time points in the training dataset (Figure 1(i)). In the testing dataset, the performance of the model decreased a bit at 1 year to a fair level (AUC: 0.674), but it came back to a good level at 3 years and 5 years (AUC: 0.724 and 0.730, Figure 1(j)). As there is no available CC dataset with OS, we utilized the early CC dataset GSE44001 with DFS to validate the predictive validity of our model. We revealed a trend that the patients had shorter DFS in the high-risk group than those in the low-risk group (*p*=0.061, Figures 1(h)). In the early CC cohort, the AUC was 0.631, 0.627, and 0.544 at 1, 3, and 5 years, respectively (Figure 1(k)), which indicates a fair performance to predict DFS in early CC patients. All the results above suggest that our FAM model can be used in the prediction of the survival status in CC patients.

### 3.2. Establishment and Validation of a Nomogram

**Figure 2:**
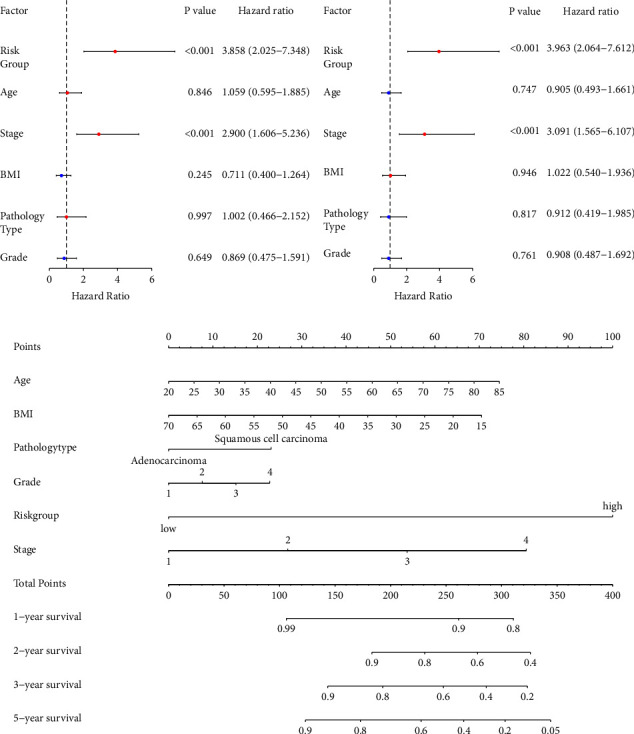
Establishment of a FAM-related clinicopathologic nomogram. Univariate Cox regression analysis (a) and multivariate Cox regression analysis (b) of the risk group based on FAM signature and clinicopathologic parameters. Bars indicate 95% CI of HR. (c) Establishment of a prognostic nomogram to predict 1-, 2-, 3-, and 5-year OS in CC patients.

In the univariate and multivariate analysis, the risk group (*p* < 0.001, HR 3.858, 95% CI: 2.025–7.348; *p* < 0.001, HR: 3.963, 95% CI: 2.064–7.612, respectively) and advanced stage (*p* < 0.001, HR: 2.900, 95% CI: 1.606–5.236; *p* < 0.001, HR: 3.091, 95% CI: 1.585–6.107, respectively) emerged as significant risk factors for worse OS (Figures 2(a) and 2(b)). We also established the univariate and multivariate Cox regression model for the RS and revealed that the RS was the independent predictor for predicting worse OS in both univariate and multivariate analysis in CC patients (*p* < 0.001, HR: 5.746, 95% CI: 3.281–10.061; *p* < 0.001, HR: 5.210, 95% CI: 2.695–10.072; Supplementary Figures 1a and 1b), while only the advanced stage showed significance in univariate analysis (*p* < 0.001, HR: 1.779, 95% CI: 1.353–2.339; Supplementary Figures 1a and 1b). In addition, the results of the PH assumption of risk score and risk group are shown in Supplementary Figures 3o, 3p, 3u, and 3v and Supplementary Table 3, and no statistically significant results were found. Taken together, our results suggest that our FAM gene signature is not inferior to traditional clinicopathological variables, such as stage, and even superior to the pathology grade and type in the clinical practice and can serve as an independent predictor of survival in CC patients. As depicted in Figure 2(c) and Supplementary Figure 1c, a higher total score according to the sum of the assigned numbers for each parameter in the nomogram was correlated with worse 1-, 2-, 3-, and 5-year OS probabilities. For instance, a patient with an advanced stage and a higher risk score would yield a total of 180 points (80 points for stage 4, and 100 points for the high-risk group), with predicted 3-year OS rates of less than 90% (Figure 2(c)).

**Figure 3:**
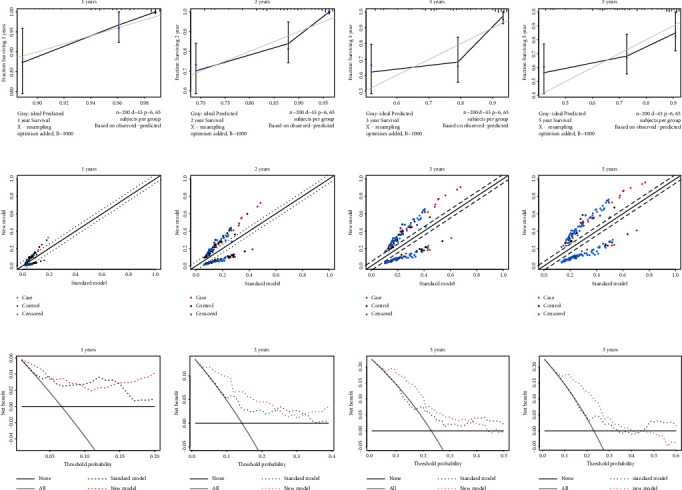
Validation of nomogram with risk group. Calibration curve to assess the consistency of predicted at 1 year (a), 2 years (b), 3 years (c), and 5 years (d) by the nomogram with risk group and actual overall survival. The net reclassification index (NRI) to evaluate the added value of new nomogram with risk group to existing prognostic models at 1 year (e), 2 years (f), 3 years (g), and 5 years (h). Decision curve analysis (DCA) to evaluate the clinical decision-making benefits of the nomogram with risk group at 1 year (i), 2 years (j), 3 years (k), and 5 years (l). NRI, net reclassification index; DCA, decision curve analysis.

To validate the risk nomogram model, the predictive performance of the nomogram was assessed by computing the discrimination index and the calibration plot of the model for the 1-, 2-, 3-, and 5-year survival. The *C*-index was 0.77 or 0.79 for our nomogram with the risk group or RS, respectively, which suggests a good discriminative ability of the nomogram. Calibration plots measure the coherence between the outcomes predicted by the nomogram models and the actual outcomes in the CC cohort. The predictions made by the nomogram model were close to the observed outcomes (1-, 2-, 3-, and 5-year survival) (Figures 3(a)–3(d) for the nomogram with risk group; Supplementary Figures 2a–2d for the nomogram with RS). In addition, to access the accuracy of movement in risk classification, we calculated the NRI for our new prognostic model with the risk group. As a result, when our new model with risk group was compared with the previous standard, NRI displayed an improved reclassification with 72.14% improvement in the prediction accuracy of 3-year survival probability and 49.98% improvement in the prediction accuracy of 5-year survival probability (Figures 3(e)–3(h) for the nomogram with risk group). For the nomogram model with RS, a 45.72% improvement in the prediction accuracy of 3-year survival probability and a 33.75% improvement in the prediction accuracy of 5-year survival probability were observed in the new model with RS (Supplementary Figures 2e–2h). Finally, DCA plots revealed the clinical utility of the nomogram model with or without the risk group and the net benefit of using both models to stratify patients relative to none (assuming that no patient will have an event). The nomogram with risk group displayed a larger net benefit across the range of risk thresholds (≥0.15 for 1-year survival, 0 to around 0.22 for 2-year survival, and 0 to around 0.4 for 3-year and 5-year survival) compared to the model with clinical variables only (Figures 3(i)–3(l)). Better net benefits were observed in the nomogram with RS in comparison to the traditional model of 1-, 2-, 3-, and 5-year survival (Supplementary Figures 2i–2l).

### 3.3. Comparison of Models Built by Other Machine Learning Methods

**Figure 4:**
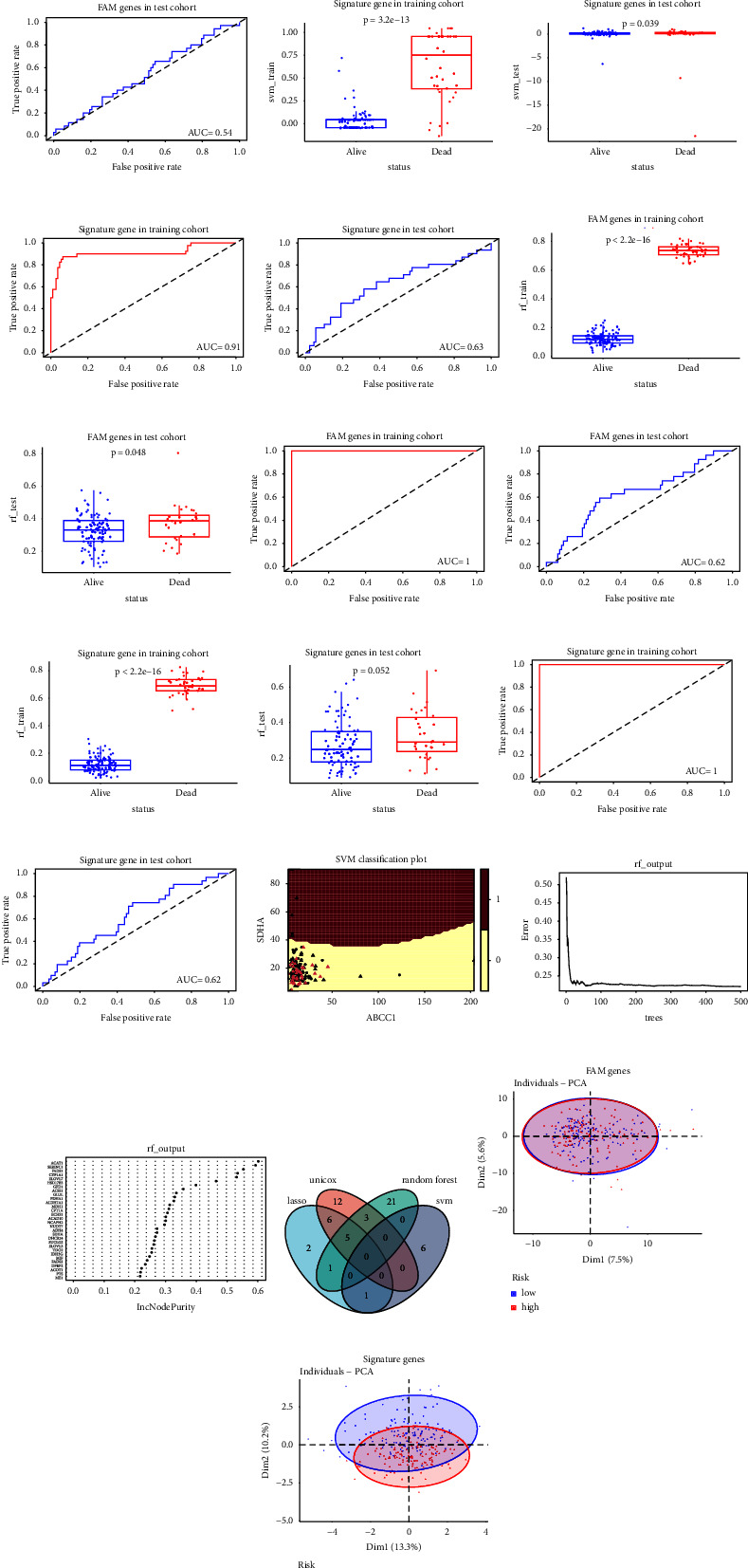
Comparison of models built by other machine learning methods. The classification capacity of the model based on FAM genes built by SVM accessed by Wilcoxon test in the training cohort (a) and the testing cohort (b) and the predictive value by AUC in the training cohort (c) and in the testing cohort (d). The classification capacity of the model based on 14 signature genes built by SVM accessed by Wilcoxon test in the training cohort (e) and the testing cohort (f) and the predictive value by AUC in the training cohort (g) and in the testing cohort (h). The classification capacity of the model based on FAM genes built by RF accessed by Wilcoxon test in the training cohort (i) and the testing cohort (j) and the predictive value by AUC in the training cohort (k) and in the testing cohort (l). The classification capacity of the model based on 14 signature genes built by RF accessed by Wilcoxon test in the training cohort (m) and the testing cohort (n) and the predictive value by AUC in the training cohort (o) and in the testing cohort (p). (q) The classification capacity of significant variables in SVM model. (r) The error rate as the trees grown in RF model. (s) The top 30 important variables by RF. (t) The Venn diagram to identify the overlapping genes by different methods. (u) Scatter plot concerning the risk distribution based on FAM genes by PCA. (v) Scatter plot based on 14 signature genes concerning the risk distribution by PCA. The error bars in (a), (b), (e), (f), (i), (j), (m), and (n) represent the standard deviation of measurements for 151 samples in training cohort or 123 samples in testing cohort. SVM, support vector machine; FAM, fatty acid metabolism; AUC, area under the curve; RF, random forest; unicox, univariate Cox regression; PCA, principal component analysis.

Next, to compare the predictive value among different models, we used the expression levels of the 309 FAM genes to construct the SVM model. The polynomial SVM model performed rather impressively with the best degree of 4 and coefficient of 0.1 in the training dataset (*p* < 0.001 by Wilcoxon test, AUC = 1, Figures 4(a) and 4(c)), but it failed in the testing dataset (*p* = 0.52 by Wilcoxon test, AUC = 0.54, Figures 4(b) and 4(d)). The significant variable list in SVM is summarized in Supplementary Table 4 (Figure 4(q)). Similar classification results were observed using the random forest algorithm, a significant result in the training cohort (*p* < 0.001 by Wilcoxon test, AUC = 1, Figures 4(i) and 4(k)), but an underwhelming result in test dataset (*p* = 0.048 by Wilcoxon test, AUC = 0.62, Figures 4(j) and 4(l)). The selection process and the top 40 important genes in the random forest are shown in Figures 4(r) and 4(s). Besides, we performed the univariate Cox regression to select the genes correlated with OS (Supplementary Table 5). The intersection of significant genes in each model is displayed in Figure 4(t) and Supplementary Table 6. Then, we employed the 14 specific genes to construct the classification by SVM and random forest to check whether the predictive value was improved. In the training cohort, the performance of the SVM and random forest model was impressive (*p* < 0.001 by Wilcoxon test, AUC = 0.91 in the SVM model; *p* < 0.001 by the Wilcoxon test, AUC = 0.1 in the random forest model; Figures 4(e), 4(g), 4(m) and 4(o)). The predictive value of the SVM model with signature genes (*p* = 0.039 by the Wilcoxon test, AUC = 0.63, Figures 4(f) and 4(h)) was slightly improved as compared to the SVM model with the FAM genes in the testing dataset. The discriminative ability of the random forest model with signature genes did not improve compared to the model with FAM genes (*p* = 0.052 by Wilcoxon test, AUC = 0.62, Figures 4(n) and 4(p)) in the testing cohort. In addition, a separation was observed using a PCA analysis using the 14-gene signature, but there was no separation using the FAM genes (Figures 4(u) and 4(v)). Taken together, the predictive ability of the LASSO-Cox model was the best among all the models we established, which was superior to the SVM and random forest models according to the AUC.

### 3.4. Identification of the FAM Phenotype concerning the Risk Grouping

The FAM remodeling in cancer contains aberrant changes in endogenous FA uptake, de novo synthesis, and *β*-oxidation to produce energy and store FA. Therefore, we further explored the FAM alteration in high-risk CC patients. The transporters of FA on the plasma membranes contain the FA transport protein family, FA binding proteins, and FA translocase [37]. We found that the FA translocase, CD36, tended to be increased (*p* = 0.067, Supplementary Figure 4e), while the solute carrier protein family 27 (SLC27) was decreased (*p* = 0.029 for SLC27A1, *p* = 0.0069 for SLC27A2, *p* = 0.00044 for SLC27A3, *p* = 0.015 for SLC27A5, Supplementary Figures 4ag, 4ah, 4ad, and 4ak) in the high-risk group, which may suggest that those cancer cells do not rely on the exogenous uptake of FA much. Since the CC cells in the high-risk group do not rely on the exogenous uptake of FA, the biosynthesis of FA from glucose, acetate, or glutamine is apparent to be important. Notably, we revealed that the de novo synthesis of FA was significantly upregulated in CC patients with high risk. The enzymes involved in the synthesis of glutamine or acetate to citrate were enhanced, including glutaminase (GLS, *p* = 0.0013) (Supplementary Figure 4ab). Moreover, the production of palmitate from citrate was promoted through the upregulated expression of ATP-citrate lyase (ACLY, *p* = 0.034, Supplementary Figure 4a), FA synthase (FASN, *p* = 0.042, Supplementary Figure 4aa), and long-chainacyl-CoA synthetase 3 (ACSL3, *p* = 0.041, Supplementary Figure 4b). Then, the saturation of FA was promoted by high expression of stearoyl-CoA desaturase (SCD, *p* = 0.018, Supplementary Figure 3af). However, the alternation of *β* oxidation was complex. Some enzymes of *β* oxidation were downregulated, such as carnitine palmitoyltransferase 1A (CPT1A, *p* = 1.9 × 10^−5^) and CPT1B (*p* = 0.00024) while the CUB domain-containing protein 1 (CDCP1) was increased (*p* = 2.6 × 10^−5^, Supplementary Figure 4g). Similarly, the elongation of FA has to be checked comprehensively, as the expression of ELOVL FA elongase 2 (ELOVL2, *p* = 0.0088) was increased while ELOVL7 (*p* < 0.001) was decreased in the high-risk group (Supplementary Figures 4k and 4p). So, we may speculate that the CC patients with high risk were featured by enhanced de novo synthesis of FA in our study.

### 3.5. Identification of DEGs and Functional Enrichment Analysis

**Figure 5:**
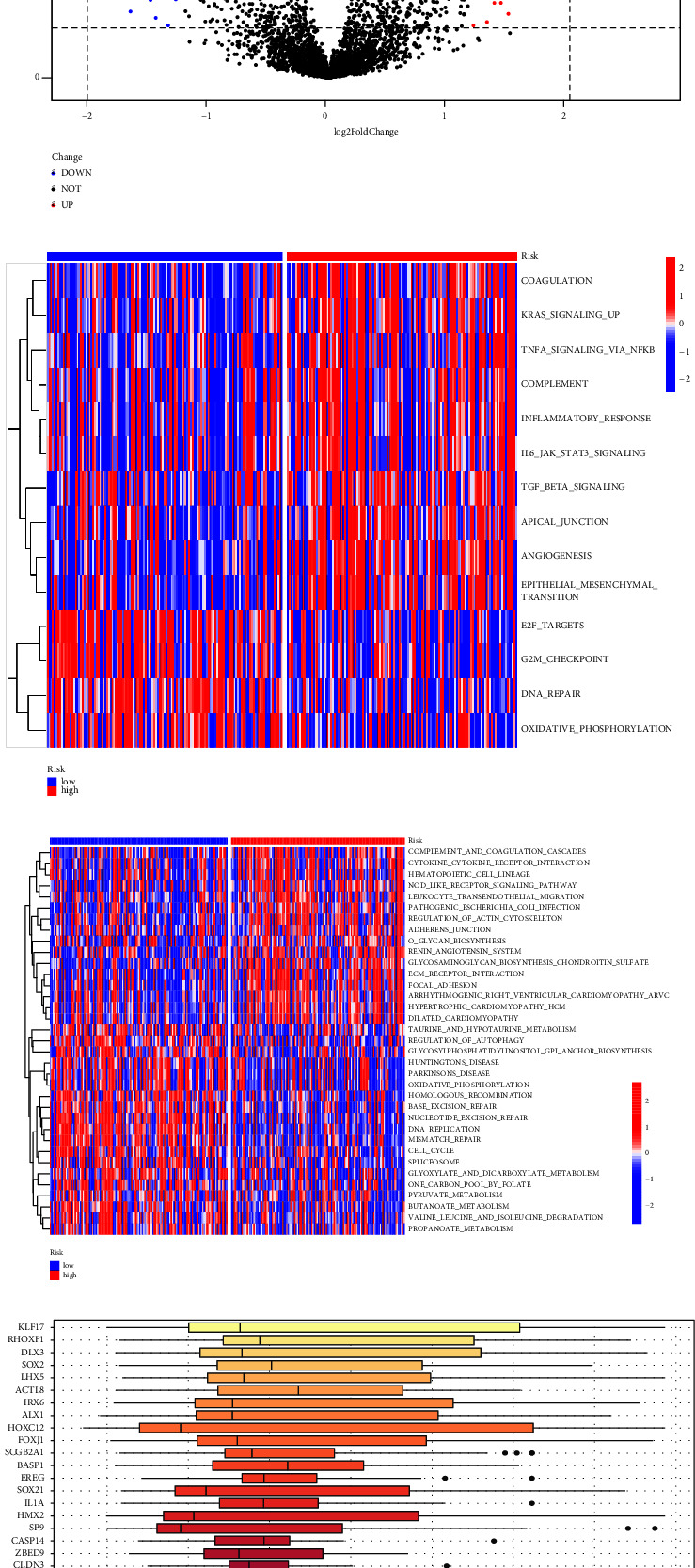
Identification of differential expression genes and functional enrichment analysis. (a) Heatmap of 51 differentially expressed genes concerning the risk groups. (b) Volcano plot exhibiting 51 DEGs with highlighting of the top 5 upregulated genes in red and top 5 downregulated genes in blue. HALLMARK (c) and KEGG (d) pathways based on risk groups by GSVA. The hub genes among DEGs identified by “friends” analysis (e) and “closeness” in Cytoscape CytoHubba (f).

To search for the regulation factors and effectors between the two groups, differential gene expression analysis was first performed. Differential expression analysis identified 51 DEGs between the two groups, in which 27 genes were upregulated, whereas 24 genes were downregulated (Figure 5(a)). The top 5 upregulated and downregulated genes are highlighted in Figure 5(b). Then, the GO and KEGG enrichment analyses were performed among the DEGs. The results of the GO analysis are demonstrated in Supplementary Figure 5a. In the KEGG analysis, the metabolic pathways were significantly enriched (*p*=0.0054, Supplementary Figures 5b and 5c). In addition, the housekeeping genes were activated in the Msigdb enrichment, among which COX6A1 and COX8A were involved in ATP synthesis and mitochondrial energy metabolism (Supplementary Figure 6a), and the metabolic genes regulated by TP53 were activated in the Reactome analysis (Supplementary Figure 6b). The results above indicate that the metabolism was enhanced in the high-risk group.

In the GSVA analysis, according to the HALLMARK gene set, we found that the coagulation, Kirsten rat sarcoma viral oncogene homolog (KRAS) signalling, tumour necrosis factor (TNF) signalling via nuclear factor *κ*B (NF*κ*B), complement and inflammatory response, interleukin 6(IL6)-Janus kinase (JAK)-signal transducer and activator of transcription 3 (STAT3) signalling, transforming growth factor *β* (TGF*β*) signalling, apical junction, angiogenesis, and epithelial-mesenchymal transition were activated in the high-risk group, whereas the E2F targets, G2M checkpoint, DNA repair, and oxidative phosphorylation were inhibited in the high-risk group (Figure 5(c)). Similar results were obtained in KEGG analysis, for example, complement and coagulation cascades were activated in the high-risk group, and oxidative phosphorylation, homologous recombination, base excision repair nucleotide excision repair, DNA replication, mismatch repair, and cell cycle were downregulated in the high-risk group (Figure 5(d)). Those results may indicate that special inflammatory mediators, TNF*α*, IL6, TGF*β*, and complements, might create an immunosuppressive microenvironment with chronic inflammation in high-risk CC patients, which support tumour progression and metastasis by activating several signalling pathways, namely, NF*κ*B, JAK-STAT3, TGF*β*, and KRAS signalling.

### 3.6. Identification of Hub Genes and Regulation Network

**Figure 6:**
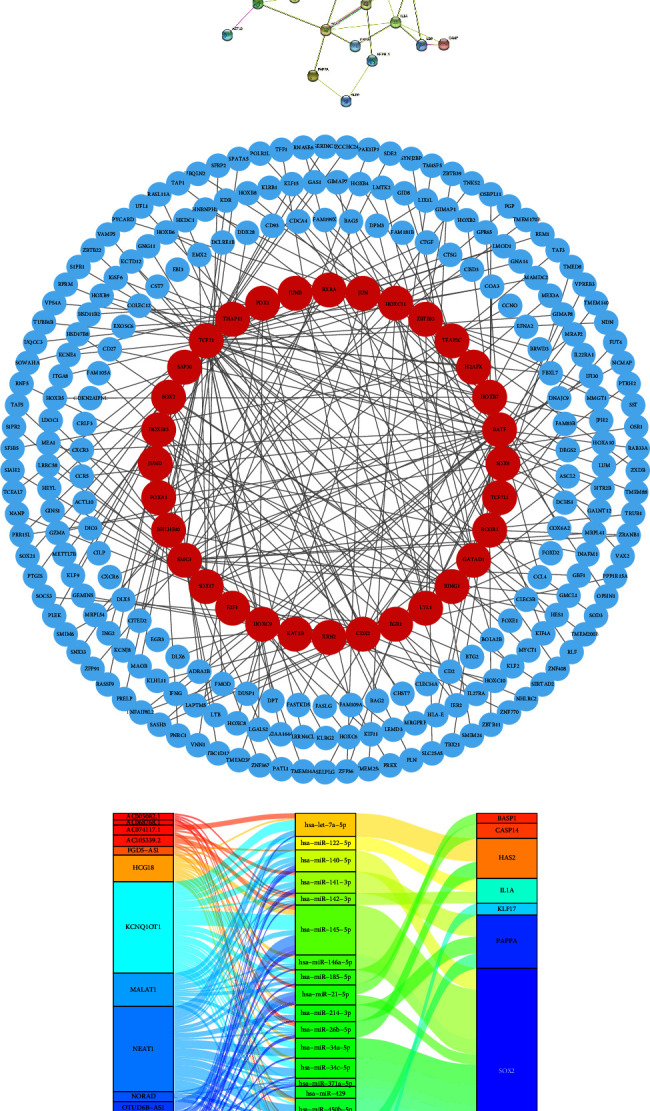
Regulation network of DEGs. (a) The direct interactions of protein among DEGs. (b) The transcriptional regulation network among DEGs. (c) The post-transcriptional regulation network of DEGs.

The hub genes among the DEGs obtained by “GOSemSim” analysis are listed in Figure 5(e). The likelihood of PPI is identified in Figure 6(a). Then, the proteins in the PPI network were further analysed by Cytoscape CytoHubba. The top 40 genes were filtered by the algorithm “closeness” as in Figure 5(f). The intersection of the hub genes derived from “friends” and “closeness” is listed in Supplementary Table 7. Besides the direct interaction of proteins among DEGs (Figure 6(a)), we also explored the transcription regulation and identified 32 transcription factors based on the DEGs as shown in Figure 6(b) (the transcription factors are listed in the Supplementary Table 8). Furthermore, the post-transcriptional regulations by miRNA and lncRNA were inferred (Figure 6(c)).

Notably, the SRY-box transcription factor 2, SOX2, was rather active. SOX2 is the centre of the transcriptional network influencing pluripotency and is essential in formation of cancer stem cells and resistance to treatment [38, 39].

### 3.7. Somatic Genomic Alternation Analysis

**Figure 7:**
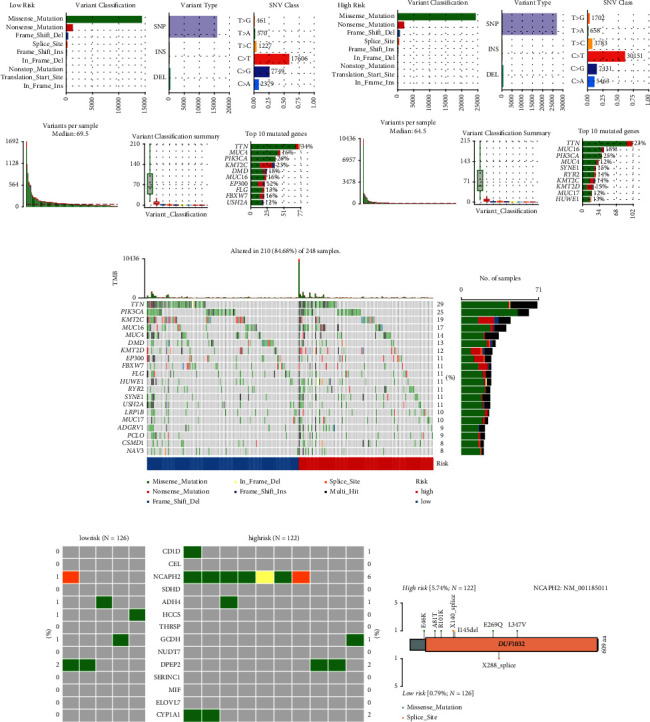
Somatic genomic alternation analysis. The summary of the mutated genes, according to variant classification, variant type and SNV class, mutation load for each sample, and variant classification type in the low-risk group (a) and the high-risk group (b). (c) The oncoplot displays the somatic mutation landscape of top 20 genes based on risk levels. (d) The mutation patterns of the 14 signature genes based on risk groups. (e) Lollipop plot displays mutation distribution and protein domains for NCAPH2 in different risk groups with labelled recurrent hotspots. SNVs, single-nucleotide variants.

First, the somatic mutation landscapes were summarized according to risk grouping (Figures 7(a) and 7(b)). The somatic variants contain single-nucleotide variants (SNVs) and small insertions/deletions (indels). In both the risk groups, the top 3 variant classifications were missense mutation, nonsense mutation, and frameshift deletion and the most frequent variant type was single-nucleotide polymorphism (SNP) (Figures 7(a) and 7(b)). SNV with *C* > *T* occurred predominantly in both groups. There were 17606 *C* > *T*base substitutions in the high risk group and 30151 *C* > *T* base substitutions in the high risk group (Figures 7(a) and 7(b)). The median of variants per sample was 69.5 in the low-risk group and 64.5 in the high-risk group (Figures 7(a) and 7(b)). Similar to the results of variants per sample, the TMB was 1.39/MB in the low-risk group and 1.29/MB in the high-risk group, suggesting low TMB in CC patients (Supplementary Figures 7c and 7d). The top three mutated genes were tinin (TTN), mucin 4 (MUC4), and phosphatidylinositol-4,5-bisphosphate 3-kinase catalytic subunit alpha (PIK3CA) in the low-risk group and TTN, mucin 16 (MUC16), and PIK3CA in the high-risk group (Figures 7(a)–7(c)). In addition, 210 samples (84.68%) were detected to have somatic mutations in the whole CC cohort (Figure 7(c)). Among them, 114 samples (90.48%) had somatic mutations in the low-risk group, and 107 (87.7%) had somatic mutations in the high-risk group (Supplementary Figures 8a and 8b). Next, we found that the mutation frequency of the signature genes differed in different risk groups (Figure 7(d)). For example, the mutation rate of NCAPH2 was 6% in the high-risk group, whereas only 1% was in the low-risk group (Figure 7(d)). The mutation pattern of the signature genes was distinguished in the respective group (Figure 7(e)), especially NCAPH2, which was identified as a potential driver gene in CC [40].

As SNPs are classified into two conversions of transitions (*A* > *G*/*G* > *A* and *T* > *C*/*C* > *T*) and four conversions of transversions (*C* > *A*/*A* > *C*, *C* > *G*/*G* > *C*, *T* > *A*/*A* > *T*, and *T* > *G*/*G* > *T*) according to the types of base substitution. Supplementary Figure 9 shows the fraction of conversions in each sample. The *C* > *T* transversion accounted for the highest incidence among the six conversions in both groups.

### 3.8. Identification of Mutation Driver and Affected Signalling Pathway

**Figure 8:**
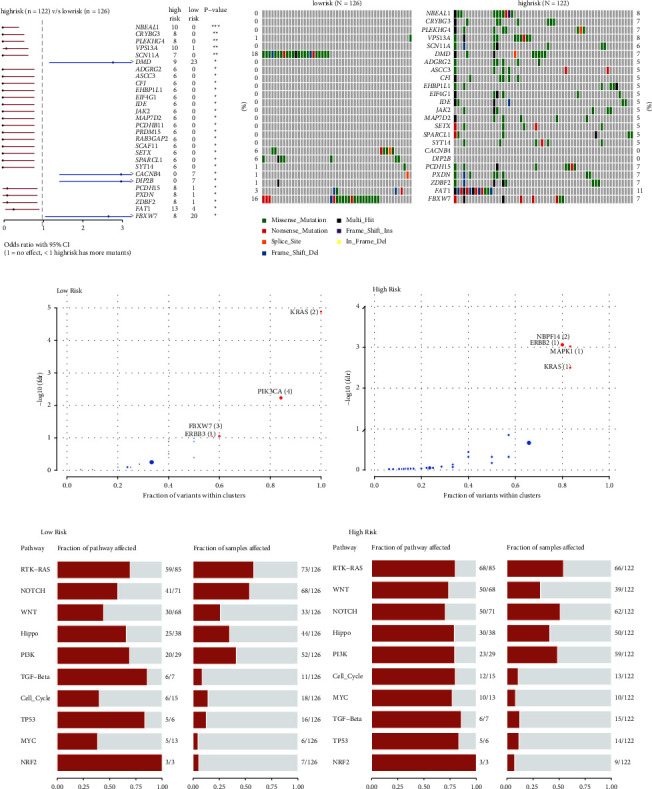
Identification of mutation driver and affected signalling pathway. (a) Differentially mutated genes between the low-risk group and the high-risk group are displayed as a forest plot. Bars indicate 95% CIs of OR. The table in the right demonstrated the number of sampleswith the mutant in the low-risk group and the high-risk group.. ^*∗∗∗*^*p* < 0.001, ^*∗∗*^*p* < 0.01, and ^*∗*^*p* < 0.05. (b) The mutation patterns of the differentially mutated genes are based on risk groups. Driver genes identified by oncodrive function in maftools in the low-risk group (c) and in the high-risk group (d), and the highlighted number with the brackets stands for closely spaced mutational clusters. The enrichment of known oncogenic signalling pathways in the low-risk group (e) and in the high-risk group (f) displayed in fraction of pathway affected and fraction of samples affected.

During the progression of cancer, initiation and promotion of tumour development are considered by driver mutations [41]. The comparison revealed 28 significant genes with differential mutation patterns concerning the risk grouping (*p* < 0.05, Figure 8(a)). Among them, 25 genes were significantly enriched in the high-risk group, and the other four were enriched in the low-risk group (Figures 8(a) and 8(b)). Furthermore, the top four driver genes, KRAS, PIK3CA, F-box and WD repeat domain containing 7 (FBXW7), and ERBB3, were identified in the low-risk group, enriched in RTK-RAS and PI3K-AKT signalling pathway (Figure 8(c)). Interestingly, the mutation of FBXW7, involved in NOTCH signalling, was detected in 16% of low-risk patients (Figure 8(b)); however, the overall mutation frequency of FBXW7 in CC was around 6% [42]. Meanwhile, neuroblastoma breakpoint family member 14 (NBPF14), ERBB2, MAPK1, and KRAS were identified as driver genes in the high-risk group, mainly enriched in the RTK-RAS-MAPK signalling pathway (Figure 8(d)). The mutation hotspots of the top four driver genes in the two cohorts are shown in Supplementary Figure 10. G12V and G12D mutations of KRAS were observed in the low-risk groups, while G12C was observed in the high-risk group and G13D was observed in both groups (Supplementary Figure 10a). The E542K and E545K mutations of PIK3CA were present in both groups, (Supplementary Figure 10b). This result was consistent with the previous result that PIK3CA is the third mutated gene in both groups (Figures 7(a) and 7(b)). The E322K of MAPK1 was found in both groups, and D321N and R135K were exclusively in the high-risk group (Supplementary Figure 10c). The R505, R479, and R465 mutations of FBXW7 mostly occurred in the low-risk group (Supplementary Figure 10d). The E872G mutation of NBPF14 was only found in the high-risk group (Supplementary Figure 10e).

**Figure 9:**
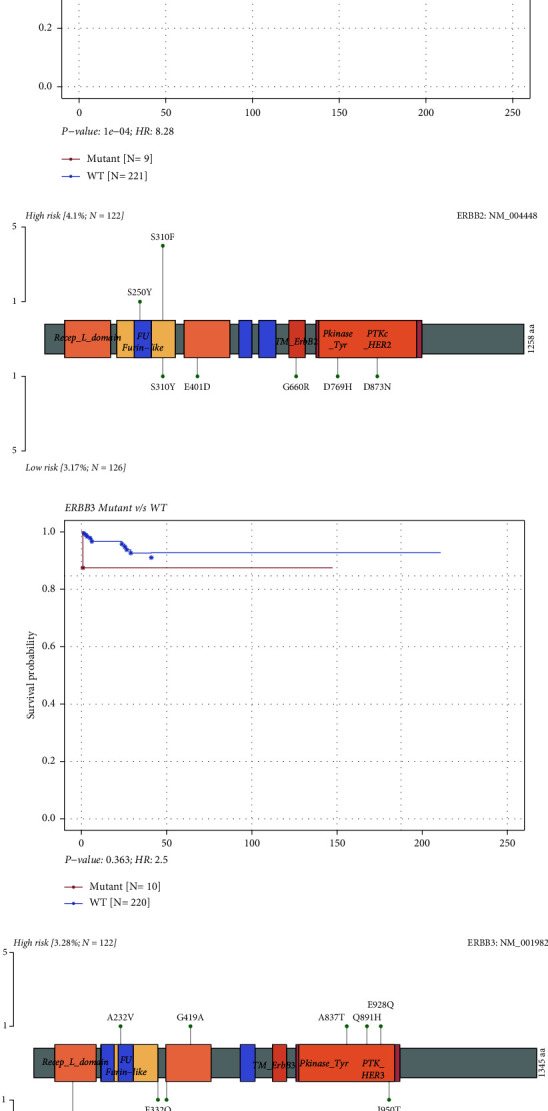
Effects of ERBB2 and ERBB3 mutations on survival. Survival analysis based on ERBB2 (a) and ERBB3 (c) mutation status. Mutant is shown in red, and WT is shown in blue. Lollipop plots displaying mutation distribution and protein domains for ERBB2 (b) and ERBB3 (d) in different risk groups with labelled recurrent hotspots. WT, wild type.

Notably, the mutation patterns of RTKs, ERBB3 and ERBB2, differed in both groups (Figures 9(b) and 9(d)). The S310F mutation of ERBB2 was only present in the high-risk group, known as oncogenic driver mutation (Figure 9(b)) [43]. The mutated ERBB2 led to worse OS in CC patients (*p*=1 × 10^−4^, Figure 9(a)), whereas the mutated ERBB3 seemed to not affect OS (*p*=0.363, Figure 9(c)). The results may partially explain the high-risk group with the ERBB2 as the driver gene had worse OS. Additionally, mutations occurred in a mutual co-occurrence manner in both groups (Supplementary Figures 7a and 7b).

Consistent with the enriched signalling pathway by driver mutation genes, the top three frequently mutated oncogenic signalling pathways were RTK/RAS, NOTCH, and PI3K in the two groups (Figures 8(e) and 8(f)). The detailed mutation patterns of RTK/RAS, PI3K, and NOTCH signalling pathways are shown in Supplementary Figures 11–13.

### 3.9. De Novo Mutational Signature Analysis

**Figure 10:**
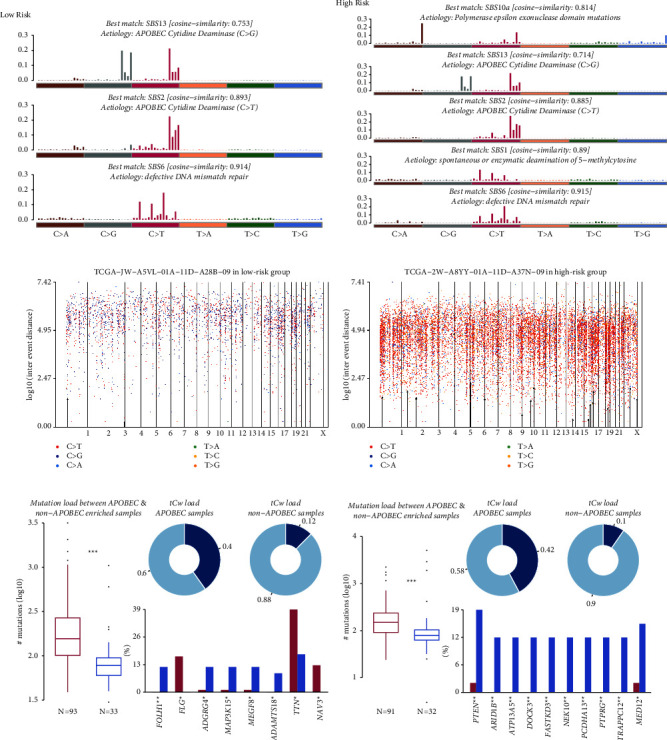
De novo mutational signature analysis. Mutation signatures matched against the validated COSMIC v3 signatures in the low-risk group (a) and the high-risk group (b). Rainfall plots for the sample with most kataegis in the low-risk group (c) and the high-risk group (d). The dots are coloured according to the SNV class. Hypermutated genomic segments are highlighted by black arrowheads. The APOBEC enrichment analysis in the low-risk group (e) and the high-risk group (f). The boxplot in the left panel demonstrated the differences in mutation load between APOBEC-enriched and non-enriched samples. The error bars represent the standard deviation. The bar plot in the right displayed the top 10 differentially mutated genes between APOBEC-enriched and non-enriched samples. ^*∗∗∗*^*p* < 0.001, ^*∗∗*^*p* < 0.01, and ^*∗*^*p* < 0.05. SNVs, single-nucleotide variants.

The progression of cancer leaves behind a distinctive mutational pattern that can display its mutagenic processes [29]. In the mutational processes analysis, we obtained three signatures as compared against the COSMIC signatures v2 in the low-risk group, while five signatures were in the high-risk group (Supplementary Figures 14a and 14b). Those signatures also were compared against the updated COSMIC signatures v3, and the results are demonstrated in Figures 10(a) and 10(b). The matched COSMIC signatures and corresponding aetiology are summarized in Table 1. Notably, the SBS10, only observed in the high-risk group, is related to defective DNA polymerase *ε* which is responsible for the exonuclease proofreading and prevention of the accumulation of mutations. The POLE gene encodes the catalytic subunit for 5′-3′ DNA polymerase and 3′-5′ exonuclease, which is important for genome stability. The incidence of POLE somatic mutations was 2.79%; however, it is 4.92% in the high-risk group while 2.38% in the low-risk group (Supplementary Figure 14c). We further identified the P254L, S297F, and V411L mutations of POLE only presented in the high-risk group (Supplementary Figure 14c).

Oncogenes are clustered around mutational hotspots [28]. Hypermutated genomic regions, named “kataegis,” are defined as those genomic segments containing six or more consecutive mutations with an average inter-mutation distance of less than or equal to 100 base pairs [44]. The formation of kataegis is hypothesized to result from multiple cytosine deamination and enrichment in *C* > *T* and *C* > *G* substitutions, which is caused by the unleashed activity of apolipoprotein B mRNA editing enzyme, catalytic polypeptide-like (APOBEC), a family of cytidine deaminases [44]. Figures 10(c) and 10(d) demonstrate the samples with the most kataegis region, TCGA-JW-A5VL in the low-risk group and TCGA-2W-A8YY in the high-risk group. Furthermore, we explored the status of APOBEC-associated mutations in both the risk groups in CC patients (Figures 10(e) and 10(f)). As a result, 73.81% (93 of 126 samples) of patients in the low-risk group were enriched for APOBEC-associated mutations (APOBEC enrichment score >2, Figure 10(e)), while 73.98% (91 of 123 samples) of patients in the high-risk group (Figure 10(f)). Furthermore, in the low-risk group, increased mutation rates within FLG, TNN, and NAV3 genes were detected in APOBEC-enriched samples, while FOLH1, ADGRG4, MAP3K15, MEGF8, and ADAMTS18 with higher mutation rates were detected in non-APOBEC-enriched samples (Figure 10(e)). However, in the high-risk group, top genes with increased mutation rates were found in non-APOBEC-enriched samples, such as PTEN and ARID1B (Figure 10(f)). Since the most frequent mutations were in the non-APOBEC-enriched samples in the high-risk group, we speculate that the mutations might be related to the SBS10a exonuclease domain mutations of POLE in the high-risk group. Interestingly, R793C, R616C, V411L, and P254L of POLE occurred in the same patient with the most kataegis region in the high-risk group, TCGA-2W-A8YY (Figure 10(d)).

### 3.10. Copy Number Variation Analysis

**Figure 11:**
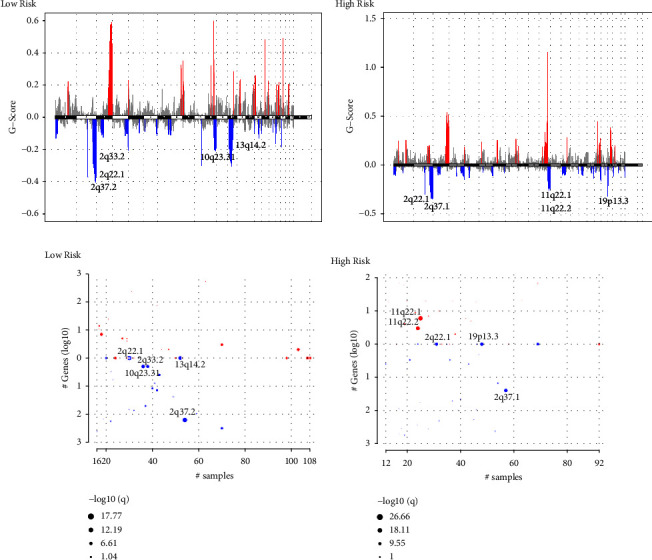
Copy number variation analysis. G-scores assigned by GISTIC for every cytoband plotted along the chromosome in the low-risk group (a) and in the high-risk group (b). GISTIC results plotted as function of altered cytobands, mutated samples, and genes involved within the cytoband in the low-risk group (c) and in the high-risk group (d). The bubble sizes are according to –log10 transformed *q* values.

We identified several CNVs in the low-risk group (Supplementary Figure 15), including the deletions on 2q33.2 (NBEAL1, CD28, CTLA4, CYP20A1), 2q22.1 (THSD7B, CXCR4), 2q37.2 (SH3BP4), 10q23.31 (FAS, PTEN), and 13q14.2 (RCBTB1) (Figures 11(a) and 11(c)). In the high-risk group, the most prevalent duplications were 11q22.2 (MMP1) and 11q22.1 (YAP1, BIRC2/3), while the most prevalent deletions were 2q37.1 (UGT1A1), 2q22.1 (THSD7B, CXCR4), and 19p13.3 (granzyme M) (Figures 11(b) and 11(d)). In the high-risk group, the deletion of granzyme M might lead to deficiency of cytotoxic lymphocytes [45] and amplification of YAP1 and MMP1 may result in enhanced angiogenesis and EMT [46, 47].

### 3.11. Identification of Neoantigens

**Figure 12:**
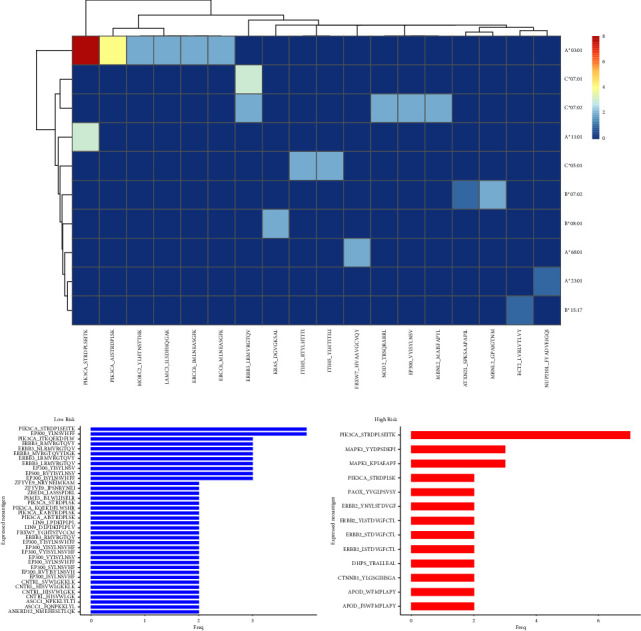
Identification of neoantigens. (a) The heatmap displaying the frequency of predicted neoantigen based on the HLA-allele. The most frequently expressed neoantigen in the low-risk group (b) and the high-risk group (c). HLA, human leukocyte antigen.

The correlation between the mutation burden and predicted neoantigen load revealed a positive linear relationship (*r* = 0.89, *p* = 1.36 × 10^−53^, Supplementary Figure 16). Sparse predicted neoantigens were shared across the population. The most common neoantigen, PIK3CA-STRDPISEITK-HLA *A∗*03 : 01, was present in 8 patients (Figure 12(a)), which might be generated as off-shelf products. The most frequent neoantigens were derived from the mutation of PIK3CA, E1A binding protein P300 (EP300), and ERBB3in the low-risk group (Figure 12(b)) and PI3KCA, MAPK1, and ERBB2 in the high-risk group (Figure 12(c)), which is coherent with the driver mutation in respective risk groups (Figures 8(c) and 8(d)). More types of neoantigen were identified in the low-risk group than in the high-risk group, and this result is partially in agreement with the previous results that higher TMB in the low-risk group (Supplementary Figures 7c and 7d).

### 3.12. Correlation between Cancer Germline Antigens and Immune Cell Infiltration

**Figure 13:**
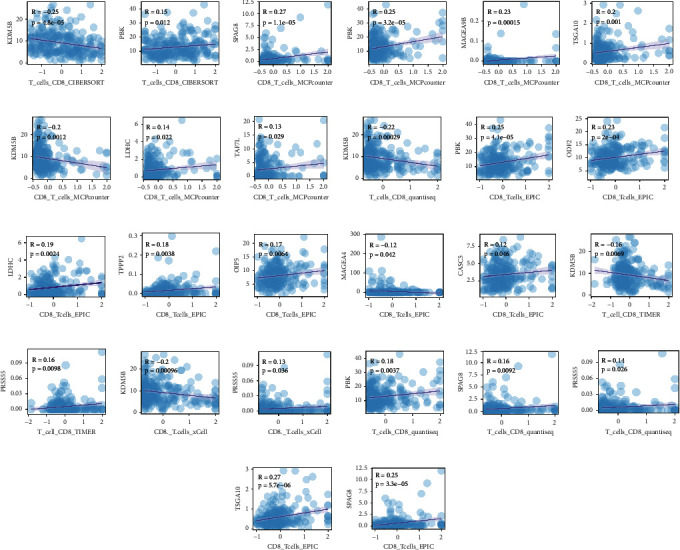
Correlation between cancer germline antigens and immune cell infiltration. The correlation between the expressions of cancer germline antigens and CD8 T cell infiltration calculated by different algorithms.

Besides neoantigens, which result from somatic mutations, the cancer antigenome also contains CGAs. CGAs are proteins normally expressed in germline cells and aberrantly expressed in tumour tissue. We found a distinct expression pattern of CGAs between low/high-risk groups (Supplementary Figure 17). There are 36 significant genes and differentially expressed CGA genes between the low- and high-risk groups (Supplementary Figure 18). The expression of a number of CGA genes, including PBK, SPAG8, TSGA10, LDHC, TAF7L, PRSS55, ODF2, TPPP2, OIP5, NUF2, TSSK6, CEP55, IGSF11, and CASC5, was significantly downregulated in the high-risk group (Supplementary Figure 18). The expression levels of MPP1 andKDM5B were enhanced in the high-risk group (Supplementary Figure 18). Next, we further explored whether the expression of CGAs is associated with the immune infiltration cells. Notably, we identified that several CGAs were positively correlated with the CD8 T cell enrichment, namely, PBK (Figures 13(b), 13(d), 13(k), and 13(v)), SPAG8 (Figures 13(c), 13(z), and 13(w)), TSGA10 (Figures 13(f) and 13(y)), LDHC (Figures 13(h) and 13(m)), TAF7L (Figure 13), ODF2 (Figure 13(l)), TPPP2 (Figure 13(n)), OIP5 (Figure 13(o)), CASC5 (Figure 13(q)), and PRSS55 (Figure 13(s) and 13(x)), which were remarkably suppressed in the high-risk group (Supplementary Figure 17), indicating an immune inhibitory environment in the high-risk group, while KDM5B, enhanced expression in the high-risk group, was negatively associated with CD8 T cell enrichment, also suggesting an inhibitory immune environment in the high-risk group (Figures 13(a), 13(g), 13(j), 13(r), and 13(t)). However, there were some CGA genes negatively correlated with CD8 T cell enrichment and most of them were in a relatively low expression level such as MAFEA9B (Supplementary Figure 19). Our results are consistent with the findings that some CGAs are significantly correlated with activated CD8T cells [32]. Moreover, we also identified a negative correlation between the enrichment of Treg cells and the expression of PBK, NUF2, TSGA10, TSSK6, ODF2, OIP5, LDHC, and CEP55 (Supplementary Figures 20 and 21). Those genes were downregulated in the high-risk group (Supplementary Figure 18), also suggesting a suppressive immune environment in the high-risk group. Interestingly, KDM5B, associated with negative T cell enrichment in the high-risk group in our analysis, was reported to promote immune evasion and reprogramming lipid metabolism [48, 49]. Our results might suggest that the FAM phenotype in the high-risk group may be related to the inhibitory immune environment.

### 3.13. Evaluation of the Cellular Composition in Tumour Microenvironment

To explore the landscape of TME, an analysis of immune infiltrating cells and other cells in the TME of CC was performed by the “IOBR” package in R [34]. The influence of infiltrating cells and risk score on survival by Cox regression are summarized in Supplementary Table 9.

**Figure 14:**
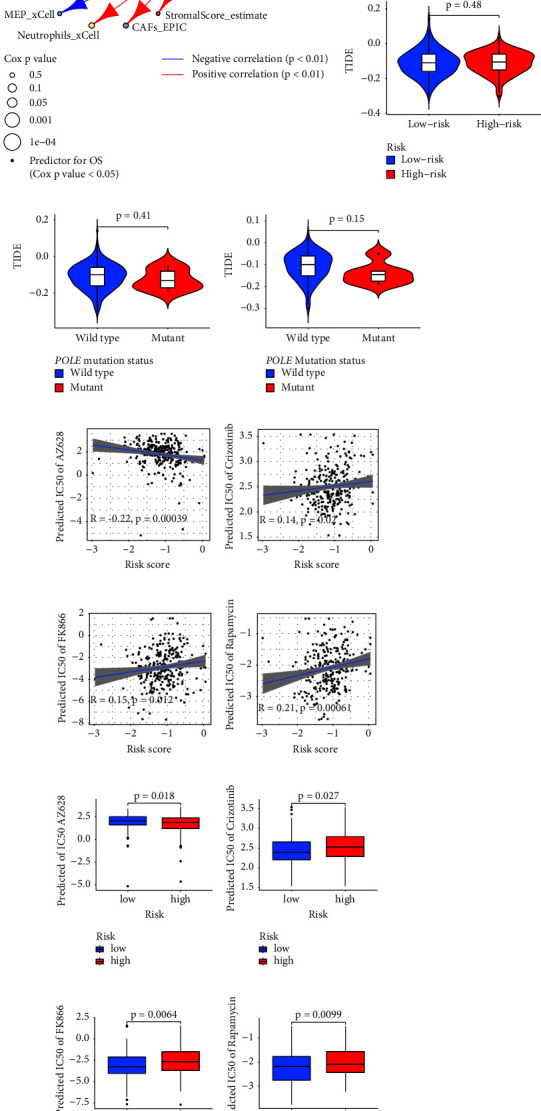
Evaluation of the cellular composition in tumour microenvironment and exploration of potential therapeutic drugs concerning prognostic models. (a) Network showing the correlation among risk score of the prognostic model and the immune cells calculated by different algorithms. Significantly positive and negative correlations are presented in red and blue line, respectively. The size of the nodes indicates the *p* values from Cox regression. The prognostic predictors for OS are marked with dots in the nodes. The violin plots display the TIDE score concerning the risk grouping (b), POLE mutation status in CC patients (c), and POLE mutation status in the high-risk group (d). A high TIDE score indicates a potential immune escape phenotype and resistance to cancer immunotherapies. The predicted sensitivity of the AZ628 (e), crizotinib (f), FK866 (g), and rapamycin (h) plotted against the risk score in the CC patients. A linear regression line and 95% confidence intervals are contained. The boxplots show the predicted sensitivity of AZ628 (i), crizotinib (j), FK866 (k), and rapamycin (l) for CC patients according to the risk grouping. OS, overall survival; TIDE, tumour immune dysfunction and exclusion; POLE, polymerase *ε*; CC, cervical cancer.

As shown in Supplementary Figures 22 and 14(a), CD8 T cells, B naïve, plasma cells, and resting mast cells by CIBERSORT [50] were negatively associated with the RS which suggests an inhibition of adaptive immune responses in the high-risk group. The CD8 T cell by CIBERSORT was associated with an improved prognosis (HR = 0.86, 95% CIs: 0.52–0.89, *p*=0.0053, Supplementary Table 9 and Figure 14(a)). However, activated mast cells by CIBERSORT were positively correlated with RS and may lead to a worse prognosis (HR = 2.31, 95% CIs: 1.76–3.04, *p*=1.93 × 10^−9^, Supplementary Table 9 and Figure 14(a)).

Moreover, endothelial cells by both MCPcounter and *x*Cell [51] were positively correlated with the RS, which coincided with our result that the genes of hallmarks of angiogenesis were significantly enriched in the high-risk group in the GSVA analysis (Figures 4(c) and 4(d)). The endothelial cells in the MCPcounter resulted in worse survival (HR = 1.37, 95%CIs: 1.02–1.84, *p*=0.0032, Supplementary Table 9 and Figure 14(a)).

Furthermore, fibroblasts by MCPcounter [52], cancer-associated fibroblasts (CAFs) by EPIC [53], and stromal score by estimate [54] were positively related to the RS, indicating that abnormal FAM may relate to enhance fibroblast activity. Meanwhile, *M*0 macrophages were inhibited, but the *M*1 macrophages were enhanced with the increasing RS, indicating a chronic inflammation featured by macrophage and lymphocyte infiltration [55].

Adipocytes were notably accumulated in the TME of the high-risk group and had a negative influence on survival (HR = 4.89, 95% CIs: 2.01–10.94, *p*=0.00035, Supplementary Table 9 and Figure 14(a)). Megakaryocyte-erythroid progenitors (MEPs) by xCell and AZ by IPS [32] were abrogated in the high-risk group (Figure 14). It has been reported that the activated mast cells, macrophages, and neutrophils can secrete the pro-inflammatory cytokines, IL-6 and TNF *α*, which may activate the IL-6-JAK-STAT3 signalling and TNF-NF*κ*B signalling in the high-risk group as the results in HALLMARK enrichment (Figures 4(c) and 4(d)). The immunosuppressive and chronic inflammatory TME may be the reason for worse OS in the high-risk CC patients in our study.

### 3.14. Exploration of Potential Therapeutic Drugs concerning Prognostic Models

According to the prediction, the RS was positively correlated with the predicted IC_50_ of crizotinib (*R* = 0.14, *p*=0.02, Figure 14(f)), FK866 (*R* = 0.15, *p*=0.012, Figure 14(g)), and rapamycin, (*R* = 0.21, *p*=0.00061, Figure 14(h)) but negatively correlated with the predicted IC_50_ of AZ628 (*R* = −0.22, *p*=0.00039, Figure 14(e)). Furthermore, the CC patients with high risk were more resistant to crizotinib, FK866, and rapamycin (*p*=0.027, *p*=0.0064, and *p*=0.0099, respectively, Figures 14(j)–14(l)). Meanwhile, the CC patients with high risk were predicted to be more sensitive to the irreversible rapidly accelerated fibrosarcoma (RAF) inhibitor AZ628 than the CC patients with low risk (Figures 14(e) and 14(i)). A further prediction of the immune therapy response revealed no difference between the two groups (*p*=0.48, Figure 14(b)). Since POLE mutations are reported to be related with good response to the immunotherapy [56], we compared the prognosis and predicted immune therapy benefits concerning the POLE mutation status. However, concerning the POLE mutation status, we did not find the improved prognosis (Supplementary Figure 14d) and predicted immunotherapy benefits either in all samples or in the high-risk group (*p*=0.41, *p*=0.15, respectively, Figures 14(c) and 14(d)). Additionally, the potential drug-target categories based on the risk grouping are summarized in Supplementary Figure 23.

## 4. Discussion

Fatty acids (FAs) are the major components of phospholipids, sphingolipids, and triglycerides and have significant roles in the production and storage of energy, synthesis of the membrane, regulation of membrane fluidity, and secondary messengers [3]. Remodeling of FAM broadly contains alterations in the transportation of FA, de novo FA synthesis in the cytosol, and *β*-oxidation of FA to generate ATP in the mitochondria in cancer [57]. Enhanced de novo FA synthesis is necessary for cancer cells to produce phospholipids for membranes and lipid rafts [58]. *β*-Oxidation of FA supplies the tumour cells with tremendous energy for aggressiveness [59].

With Pap smear-based screening and HPV vaccination, the incidence of CC decline significantly in high-income countries [1]. However, CC is still the fourth most commonly diagnosed cancer and the fourth leading cause of cancer deaths in women worldwide [1]. Especially in transitioning countries, patients diagnosed at advanced stages lack efficient therapy [43]. On the other hand, as mentioned in previous research, diverse activation patterns of metabolic signalling pathways may be the reason for the molecular diversity of CC [60]. Therefore, we constructed a valid prognostic model based on FAM genes to distinguish CC patients at different risks. By LASSO-Cox regression, we obtained a prognostic model with good to fair performance. Other models built by the SVM and the random forest did not reach a good performance in the testing cohort. The nomogram for clinical application also achieved a good performance in calibration curves, NRI, and DCA analysis. Therefore, our model is easy to use and robust. In our FAM signature, SDHD is vital for cell growth and metabolism [61]. HCCS participates in oxidative phosphorylation and apoptosis [62]. SERINC1 is involved in serine-derived lipids [63]. THRSP can maintain mitochondrial function and regulate sphingolipid metabolism in human adipocytes [64]. Importantly, we identified that the high-risk CC patients were featured by enhanced de novo synthesis of FA.

Then, we set out to find out the underlying mechanisms leading to different FAM phenotypes and OS in our model. Besides the activation of metabolic pathways in the high-risk CC patients, our FAM phenotypes are also related to increased inflammatory responses. The inflammatory factors, such as complements, IL6, TNF*α*, and TGF*β*, and the inflammatory responses were enriched in the high-risk group. The inflammatory TME is also verified from cell levels. With the increase of RS and the infiltration of CD8 T cells, B naïve, plasma cells, and resting mast cells, *M*0 macrophages were suppressed, while neutrophils, activated mast cells, and *M*1 macrophages were boosted. The impaired infiltration of CD8 T cells may lead to immunosuppressive TME and a worse prognosis in CC patients with high RS. Next, we identified several CGAs which were associated with CD8 T cell enrichment. However, those genes were downregulated in the high-risk group, which might be one reason for the inhibitory immune TME. Notably, enriched adipocytes in the high-risk group are reported to secrete a variety of inflammatory cytokines and adipokines, such as TNF*α* and IL-6, recruiting lymphocytes, and macrophages [65].

Moreover, in the GSVA analysis, the angiogenesis and EMT were also enhanced in the high-risk group, which is in agreement with the results that endothelial cells, CAFs, and stromal score enrichment are positively associated with RS. CAFs can be derived from the migration of adipocytes [66], and endothelial and epithelial cells, through endothelial or epithelial to mesenchymal transition [67, 68]. CAFs have been reported to induce EMT and enhance angiogenesis and immunosuppression in TME [69, 70]. Besides the production of free FAs to support metastatic cancer cell survival [71], enriched adipocytes can promote the EMT of tumour cells and stabilize vascularization [72, 73].

Above all, we might confer that aberrant FAM may trigger tumour-extrinsic inflammation, which leads to an immunosuppressive, proangiogenic, and pro-tumoural microenvironment [74].

Oncogenic signalling pathways can directly regulate FAM enzymes to shape tumour lipidome. We identified PIK3CA as the frequently mutated gene in both risk groups, as in the literature [75]. E542K and E545K mutations in CC patients, activating mutations of the PIK3A helical domain, are considered to be correlated with APOBEC mutagenesis [60]. In agreement with the finding that PI3K/AKT pathway increases FA synthesis while suppressing the *β*-oxidation in diabetes [76], we also found that the CC patients were featured by enrichment of PI3K/AKT signalling and enhanced de novo FA synthesis.

Besides oncogenic mutations in PICK3CA, aggravated stimulation from RTKs can activate the PI3K-AKT signalling. We identified ERBB3 in the low-risk group and ERBB2 in the high-risk group as driver mutations. Especially the S310F in the high-risk group is most frequent among HER2 extracellular domain mutations, which can form an active heterodimer with the EGFR [77]. The activity of HER2 is stabilized and activated by MUC4, the second mutated gene in the low-risk group and the fourth mutated gene in the high-risk group [78]. Furthermore, the recruitment of PI3K to activated ERBB3 can be promoted by the interaction between MUC4 and ERBB2 [79]. HER2-positive tumours are featured by sustained de novo synthesis of lipids [80].

Besides the PI3K-AKT signalling pathway, the RTKs also activate the RAS/MAPK signalling transduction [81]. In the RAS/MAPK signalling, KRAS mutation in both groups and MAPK1 mutation in the high-risk group were also identified as driver mutations. KRAS is expressed by the uterus at a high level [82]. G12V and G12D were observed in the low-risk group, which are the most frequent mutations across tumour types [82]. G12V and G12D are weak drivers in colorectal cancer and lung adenocarcinoma with smoking; however, in endometrial cancer, they are considered major drivers [82, 83]. G12C was observed in the high-risk group, which is coherent with the KRAS-G12C as a major driver in lung adenocarcinoma with smoking [29]. G13D of KRAS, presented in both CC groups with the signature of mismatch repair deficiency, is reported to be associated with mismatch repair deficiency signatures in gastric and endometrial tumours [82]. MAPK1 E322K can hyperactivate EGFR and serve as a biomarker for erlotinib sensitivity in head and neck squamous cell cancer [84]. MAPK1 E322K was observed in both CC groups, coherent with the previous study [43]. D321N and R135K of MAPK1 were observed only in the high-risk group, which can enhance EGFR activation [85]. D321N has been reported to contribute high sensitivity to erlotinib, and R135K confers moderate sensitivity to erlotinib in head and neck squamous cell carcinoma [85], as potential therapeutic targets in high-risk CC patients. KRAS/ERK (MAPK1) signalling can increase the biosynthesis of acetyl-CoA from acetate and the expression of FASN and SCD in the de novo FA synthesis [86, 87].

In addition, the NOTCH signalling pathway was found to be activated in CC patients. The mutations of FBXW7, involved in NOTCH signal transduction, were detected in the low-risk group [88]. FBXW7, a tumour suppressor, can recognize the substrates, namely, Cyclin E, c-Myc, Mcl, mTOR, Jun, and NOTCH, for the component of the SCF E3 ubiquitin ligase [89]. The mutational hotspots of FBXW7 R505, R479, and R465 in the substrate binding domain, WD40 motif, were observed exclusively in the low-risk group, which may impair the ubiquitylation and degradation of specific substrates [90]. Previous studies have suggested that FBXW7 mutations strengthen the interaction among cancer-initiating cells via the NOTCH signalling pathway [42].

In the mutational process analysis, three signatures, SBS13, SBS2, and SBS6, were present in all CC patients. SBS13 and SBS2 mutations often occur in the kataegis in the same samples [91]. Both SBS2 and SBS13 are mainly associated with APOBEC hyperactivation, which may reflect the innate immune response to the virus, retrotransposon jumping, or tissue inflammation in cancer [91]. Therefore, we can infer that SBS2 and SBS13 may represent the damage to the genome in the context of HPV infection and persistent inflammation caused by aberrant FAM in CC patients. SBS6 is featured predominantly by *C* > *T* at NpCpG mutations leading to substitution and small indels termed as microsatellite instability caused by defective DNA mismatch repair, which is coherent with the findings that G13D mutation of KRAS, related to defective mismatch repair, was present in CC patients [91]. Acquisition of SBS1 mutations in the high-risk group referred to as a cell division/mitotic clock correlates with time or age and the rates of stem cell division which reflects the endogenous mutation process [91]. The SBS10a, only present in the high-risk group, may be generated by POLE exonuclease domain mutations. The exonuclease domain of POLE recognizes and removes wrong bases generated during replication [92]. The mutations in the exonuclease of POLE, referred to as hypermutators, cause a 10-to-100-fold increase in the mutation rate during replication [92], which is in accordance with the interesting finding that CC patients with several hypermutators had the most kataegis in the high-risk group. The mutations of S297F and V411L of POLE were reported to be hotspot mutations and associated with high TMB in endometrial carcinoma, which was present exclusively in the high-risk group in our study [93]. There are also some shreds of evidence that the non-exonuclease domain mutations of POLE have pathogenicity. The patients with POLE mutations had a high immune response and good prognoses in endometrial carcinoma [94]. However, we did not observe either a good prognosis or an improved prediction of immune therapy response according to the mutation status of POLE. According to the instruction, a high TIDE score indicates a potential immune escape phenotype and resistance to cancer immunotherapies. We might infer cautiously that the POLE mutation of exonuclease might lead to a good immune therapy response since the TIDE score had a decreasing trend in the high-risk group with mutated POLE [35]. Due to the insufficient sample size with POLE mutation in the CC cohort and follow-up information, further verification is needed.

In the following exploration of potential therapies for high-risk CC patients, we found that the CC patients with high risk might be more resistant to rapamycin (an allosteric inhibitor of mTOR), crizotinib (an adenosine triphosphate inhibitor of receptor tyrosine kinases), and FK866 (nicotinamide phosphoribosyltransferase inhibitor). However, AZ628 might be the potential therapeutic option for CC patients with high risk, which is reported to cause suppression of RAF/ERK signalling in KRAS mutant lung cancer [95]. We also identified that the neoantigen PIK3CA-STRDPISEITK-HLA *A∗*03 : 01 with relatively high frequency in CC patients might also be a treatment option. Hence, our results might improve current treatment strategies to defeat CC.

Concerning the limitation of our study, the gene expression analyses may not provide a direct reflection of enzyme activity or dependencies on specific metabolic pathways, and further experiments are needed to verify the FAM phenotypes, the altered signalling pathways, and the efficiency of those potential drug targets. Next, presently very limited open data on CC are available, additional studies are required to validate our prognostic model. Moreover, we did not consider HPV infection status in our model which is proved as an important factor in the progression of CC [96]. Disturbed FAM can influence chronic inflammation, persistent HPV infection, and carcinogenesis [97, 98].

According to our aforementioned results, we propose that distinct mutated driver genes may lead to different FAM features, and aberrant FAM then results in the differential infiltration and function of cells in TMEs, ultimately leading to different prognoses in CC.

## 5. Conclusions

In this study, we constructed a reliable model with 14 FAM-related genes by LASSO-Cox regression, by which we achieved a good risk stratification in cervical cancer patients. With the risk grouping, a feasible nomogram was established and validated. To understand the underlying mechanism, we found the high-risk samples featured by activated de novo fatty acid synthesis, epithelial to mesenchymal transition, angiogenesis, and inflammation response, which might be caused by mutations of oncogenic driver genes in RTK/RAS, PI3K, and NOTCH signalling pathways. Especially, the oncogenic mutations of ERBB2, only present in the high-risk group, led to worse survival. Besides the hyperactivity of cytidine deaminase and deficiency of mismatch repair, the mutations of POLE might be partially responsible for the gene mutation in patients with high risk. Moreover, increasing RS was found to be related to chronic inflammatory and suppressive immune microenvironment. The reduced expression of CGAs might result in the reduction of CD8 T cell infiltration in the high-risk group. Finally, the RAF inhibitor AZ628 was predicted to be sensitive in patients with high risk. Our findings provide a novel insight for personalized treatment in CC.

## Figures and Tables

**Table 1:** The major mutation signatures.

Risk group	Clustered signatures in our study	Most similar COSMIC signature v2	Aetiology of COSMIC signature v2	Most similar COSMIC signature v3	Aetiology of the COSMIC signature v3
Low risk	Signature 1	COSMIC 13	APOBEC cytidine deaminase (*C* > *G*)	SBS13	APOBEC cytidine deaminase (*C* > *G*)
	Signature 2	COSMIC 2	APOBEC cytidine deaminase (*C* > *T*)	SBS2	APOBEC cytidine deaminase (*C* > *T*)
	Signature 3	COSMIC 6	Defective DNA mismatch repair	SBS6	Defective DNA mismatch repair

High risk	Signature 1	COMSIC10	Defective polymerase POLE	SBS10a	Polymerase epsilon exonuclease domain mutations
	Signature 2	COSMIC 13	APOBEC cytidine deaminase (*C* > *G*)	SBS13	APOBEC cytidine deaminase (*C* > *G*)
	Signature 3	COSMIC 2	APOBEC cytidine deaminase (*C* > *T*)	SBS2	APOBEC cytidine deaminase (*C* > *T*)
	Signature 4	COSMIC 1	Spontaneous deamination of 5-methylcytosine	SBS1	Spontaneous or enzymatic deamination of 5-methylcytosine
	Signature 5	COSMIC 6	Defective DNA mismatch repair	SBS6	Defective DNA mismatch repair

SBS, single base substitution; APOBEC, apolipoprotein B mRNA editing enzyme, catalytic polypeptide-like; COSMIC, Catalogue of Somatic Mutations in Cancer.

## Data Availability

The data used to support the findings of this study are available from the corresponding author upon request.

## Supplementary Materials

Supplementary Materials Supplementary Table 1: fatty acid metabolism-related genes. Supplementary Table 2: the LASSO genes and their coefficient. Supplementary Table 3: the results of PH assumption. Supplementary Table 4: the significant genes in SVM model. Supplementary Table 5: the significant genes in univariate model. Supplementary Table 6: the intersection of significant genes in different models. Supplementary Table 7: the intersection of hub genes by “friends” analysis and “closeness” analysis. Supplementary Table 8: the transcription factors. Supplementary Table 9: the influence of infiltrating cells and risk score on survival by Cox regression. Supplementary Figure 1: construction of FAM-related clinicopathologic nomogram with risk score. Supplementary Figure 2: validation of nomogram with risk group. Supplementary Figure 3: results of PH assumption. Supplementary Figure 4: the expression levels of pivotal enzymes of FAM based on the risk groups. Supplementary Figure 5: functional enrichment according to GO and KEGG. Supplementary Figure 6: functional enrichment according to Msigdb and Reactome. Supplementary Figure 7: the cooccurrence of mutations and tumour mutation burden in CC patients. Supplementary Figure 8: the mutation pattern of most mutated genes in CC patients. Supplementary Figure 9: summary of transition and transversion in CC patients. Supplementary Figure 10: the mutation hotspots of oncogenic driver genes. Supplementary Figure 11: the distribution of mutated genes in oncogenic RTK-RAS pathway. Supplementary Figure 12: the distribution of mutated genes in oncogenic PI3K pathway. Supplementary Figure 13: the distribution of mutated genes in oncogenic NOTCH pathway. Supplementary Figure 14: comparison against known signatures and mutation hotspot of POLE and their influence on survival. Supplementary Figure 15: genome-wide distribution of chromatin amplification and deletion. Supplementary Figure 16: correlation between expressed neoantigen load and TMB. Supplementary Figure 17: expression levels of cancer germline antigens. Supplementary Figure 18: differentially expressed cancer germline antigens. Supplementary Figure 19: association between cancer germline antigen expression and the CD8 T cell infiltration in the tumour microenvironment. Supplementary Figure 20: the association between cancer germline antigen expression and the Treg cell infiltration in the tumour microenvironment. Supplementary Figure 21: the association between cancer germline antigen expression and the Treg cell infiltration in the tumour microenvironment. Supplementary Figure 22: distribution of infiltrating cells. Supplementary Figure 23: potential drug-target categories.
