# Supplementary material for: Prognosis and Characterization of Microenvironment in Cervical Cancer Influenced by Fatty Acid Metabolism-Related Genes
Source: J Oncol. 2023 Mar 8;2023:6851036. doi: 10.1155/2023/6851036 (PMC10017219; doi:10.1155/2023/6851036)
Supplement: Supplementary Materials — Supplementary Table 1: fatty acid metabolism-related genes. Supplementary Table 2: the LASSO genes and their coefficient. Supplementary Table 3: the results of PH assumption. Supplementary Table 4: the significant genes in SVM model. Supplementary Table 5: the significant genes in univariate model. Supplementary Table 6: the intersection of significant genes in different models. Supplementary Table 7: the intersection of hub genes by “friends” analysis and “closeness” analysis. Supplementary Table 8: the transcription factors. Supplementary Table 9: the influence of infiltrating cells and risk score on survival by Cox regression. Supplementary Figure 1: construction of FAM-related clinicopathologic nomogram with risk score. Supplementary Figure 2: validation of nomogram with risk group. Supplementary Figure 3: results of PH assumption. Supplementary Figure 4: the expression levels of pivotal enzymes of FAM based on the risk groups. Supplementary Figure 5: functional enrichment according to GO and KEGG. Supplementary Figure 6: functional enrichment according to Msigdb and Reactome. Supplementary Figure 7: the cooccurrence of mutations and tumour mutation burden in CC patients. Supplementary Figure 8: the mutation pattern of most mutated genes in CC patients. Supplementary Figure 9: summary of transition and transversion in CC patients. Supplementary Figure 10: the mutation hotspots of oncogenic driver genes. Supplementary Figure 11: the distribution of mutated genes in oncogenic RTK-RAS pathway. Supplementary Figure 12: the distribution of mutated genes in oncogenic PI3K pathway. Supplementary Figure 13: the distribution of mutated genes in oncogenic NOTCH pathway. Supplementary Figure 14: comparison against known signatures and mutation hotspot of POLE and their influence on survival. Supplementary Figure 15: genome-wide distribution of chromatin amplification and deletion. Supplementary Figure 16: correlation between expressed neoantigen load and TMB. Supplementary [file 6851036.f1.zip › supplementary figure 1-23 (1).pdf]

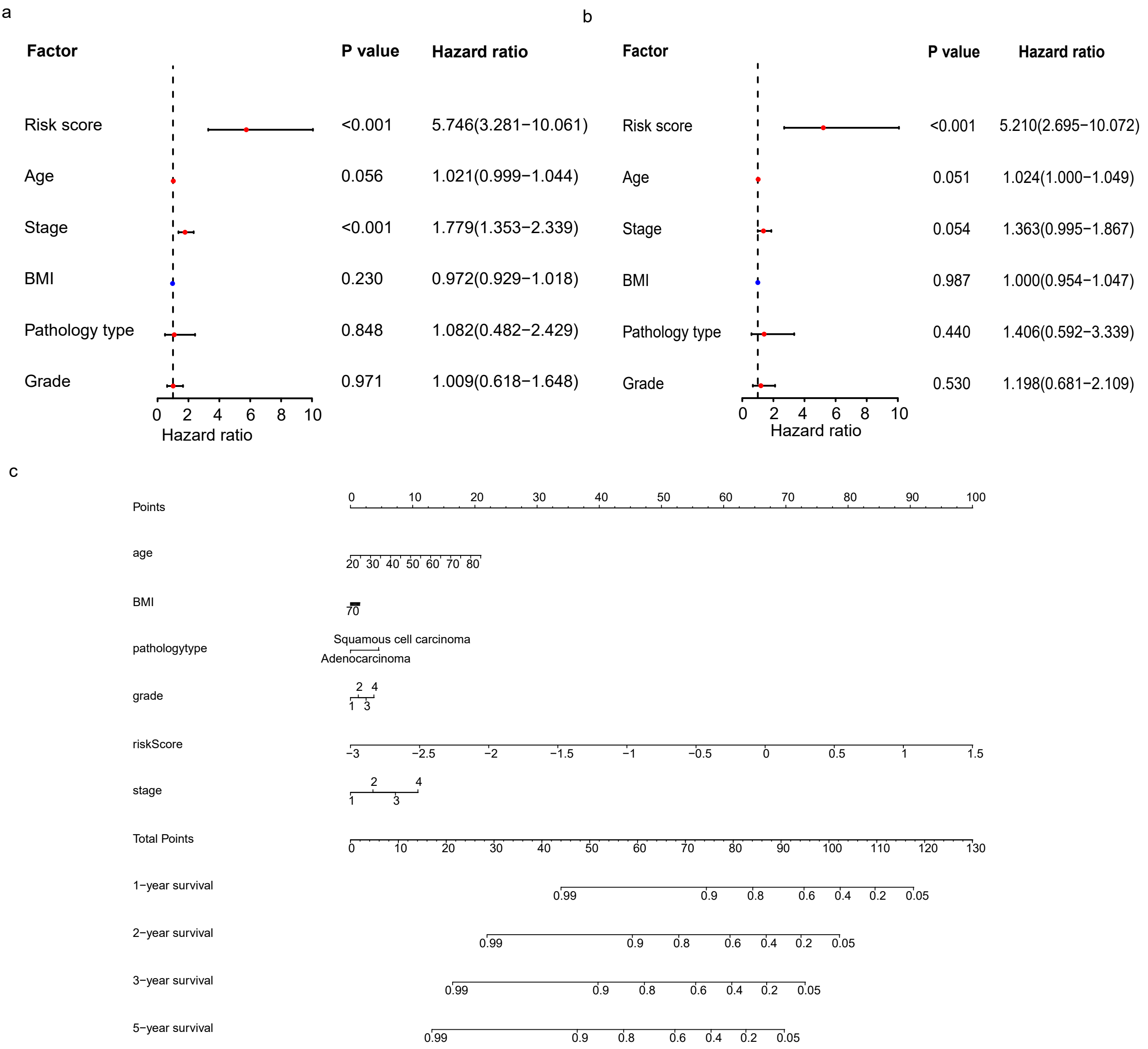

Supplementary Figure 1. Construction of FAM related clinicopathologic nomogram with risk score

Univariate Cox regression analysis (a) and multivariate Cox regression analysis (b) of the risk score based on FAM signature and clinical parameters. (c) Establishment of a prognostic nomogram with risk score to predict 1-, 2-, 3-, and 5-year OS in CC patients.

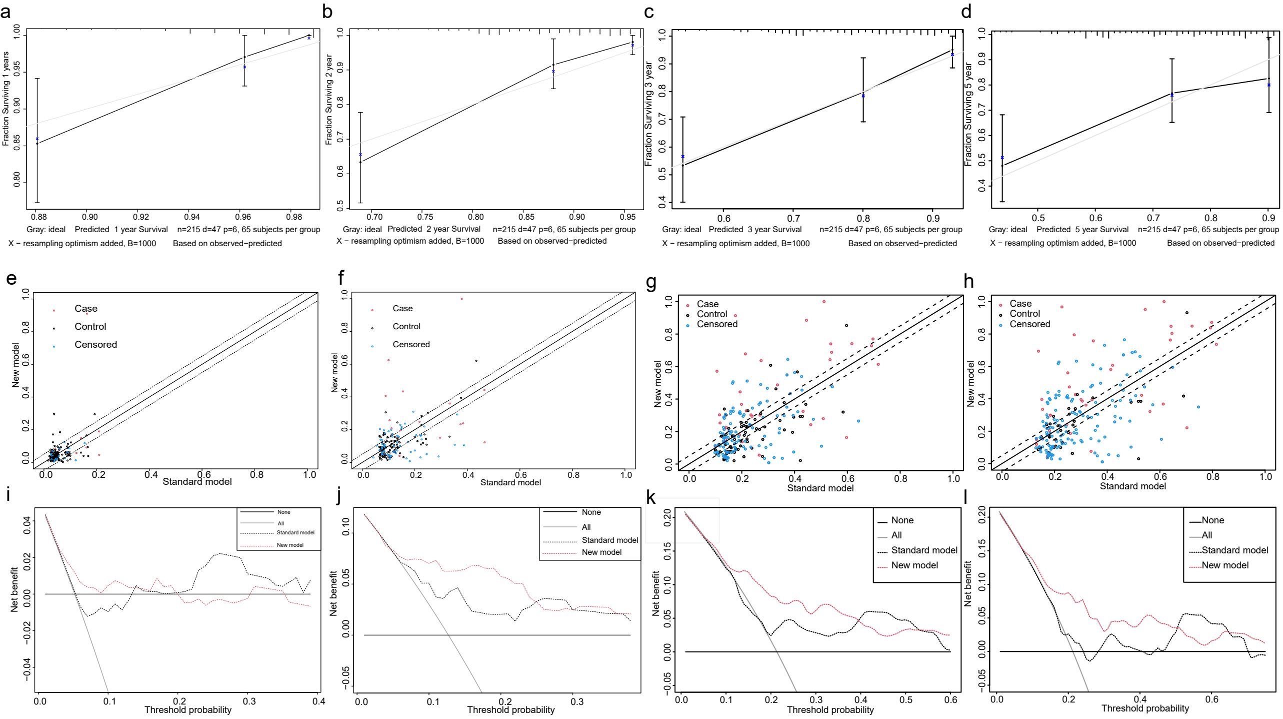

Supplementary Figure 2. Validation of nomogram with risk group.

Calibration curve to assess the consistency of predicted at 1 year (a), 2 years (b), 3 years (c), and 5 years (d) by the nomogram and actual overall survival. The net reclassification index (NRI) to evaluate the added value of new nomogram to existing prognostic models at 1 year (e), 2 years (f), 3 years (g), and 5 years (h). Decision curve analysis (DCA) to evaluate the clinical decision-making benefits of the nomogram at 1 year (i), 2 years (j), 3 years (k), and 5 years (l).

NRI, net reclassification index; DCA, Decision curve analysis;

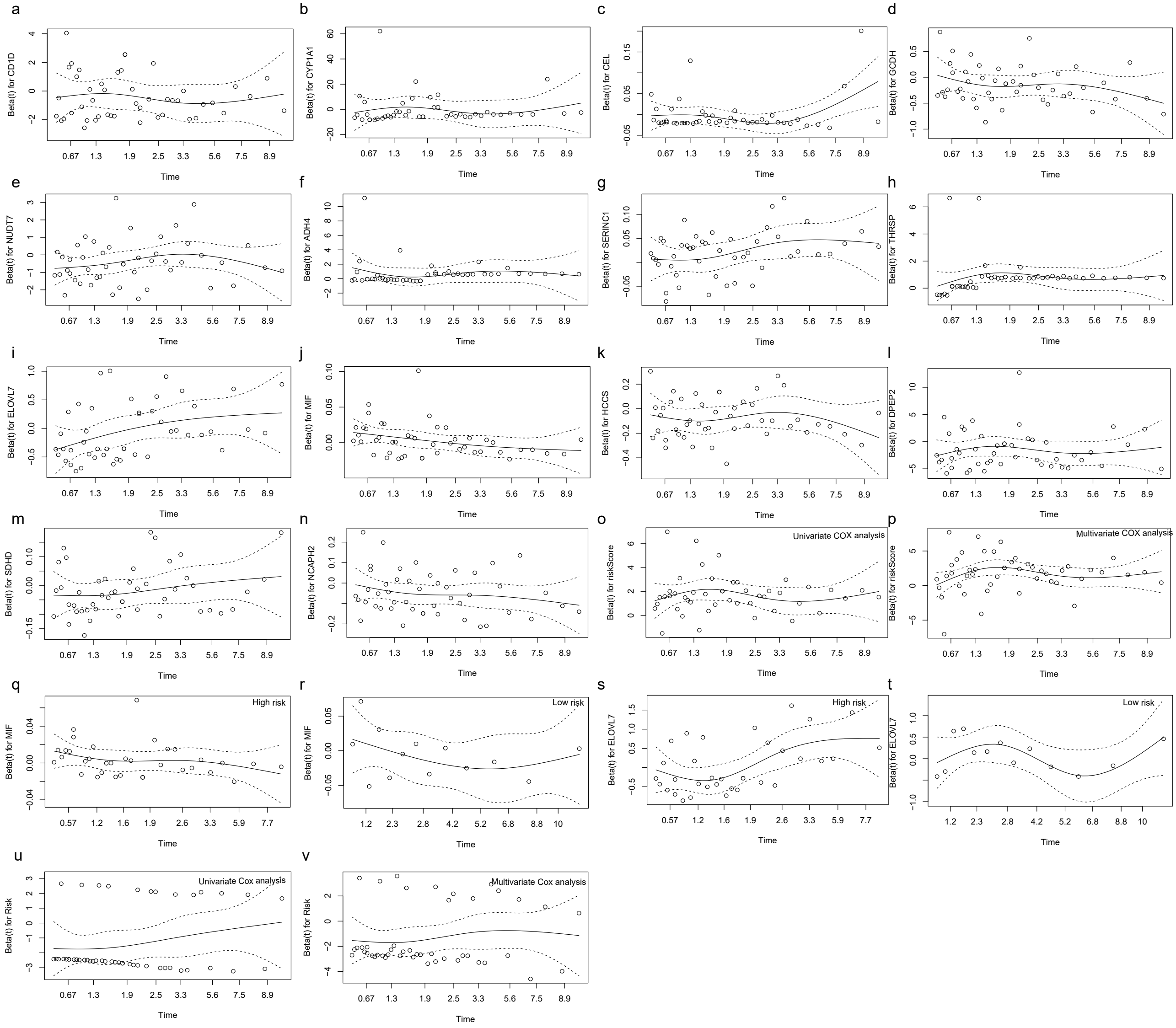

Supplementary Figure 3. Results of the PH assumption.

Results of the PH assumption on the 14 signature genes (a-n), *MIF* grouped by risk (q and r), *ELOVL* grouped by risk (s and t), risk score in univariate Cox analysis and multivariate Cox analysis (o and p), and risk grouping in univariate Cox analysis and multivariate Cox analysis (u and v).

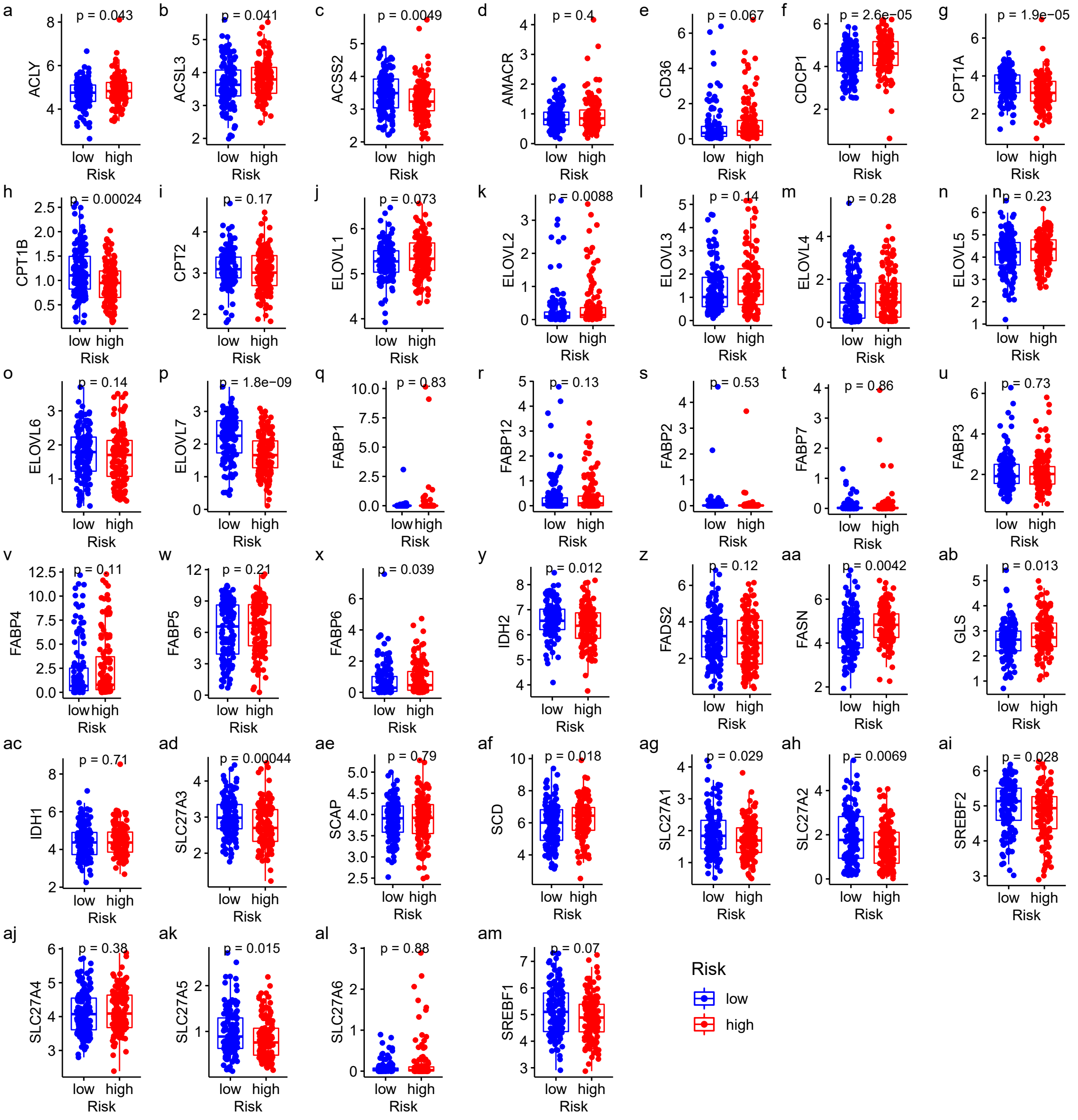

Supplementary Figure 4. The expression levels of pivotal enzymes of FAM based on the risk groups

FAM, fatty acid metabolism

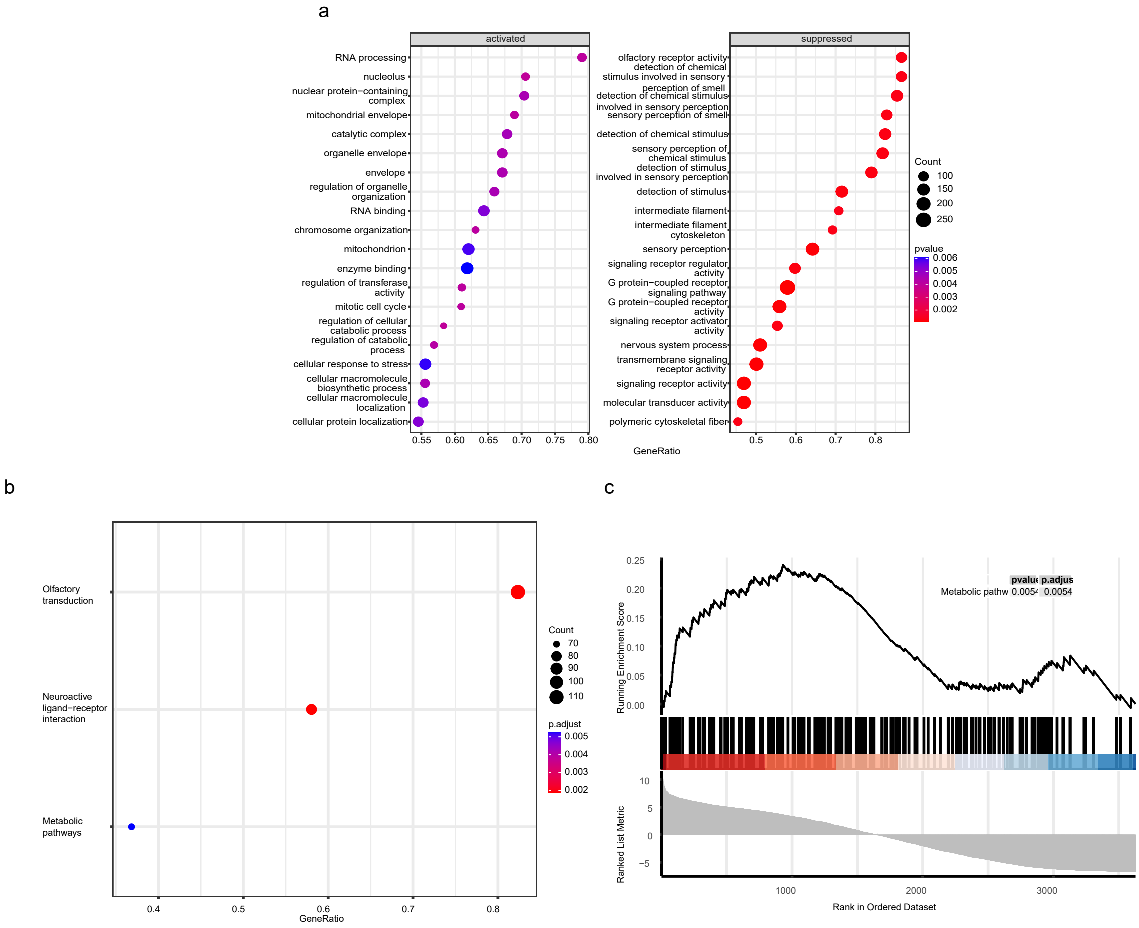

Supplementary Figure 5. Functional enrichment according to GO and KEGG

The bubble plots depicting GO enrichment results (a) and KEGG enrichment results (b) based on differentially expressed genes. (c) Positive enrichment plot for metabolic pathway gene set among the differentially expressed genes.

GO， Gene Ontology； KEGG， Kyoto Encyclopedia of Genes and Genomes

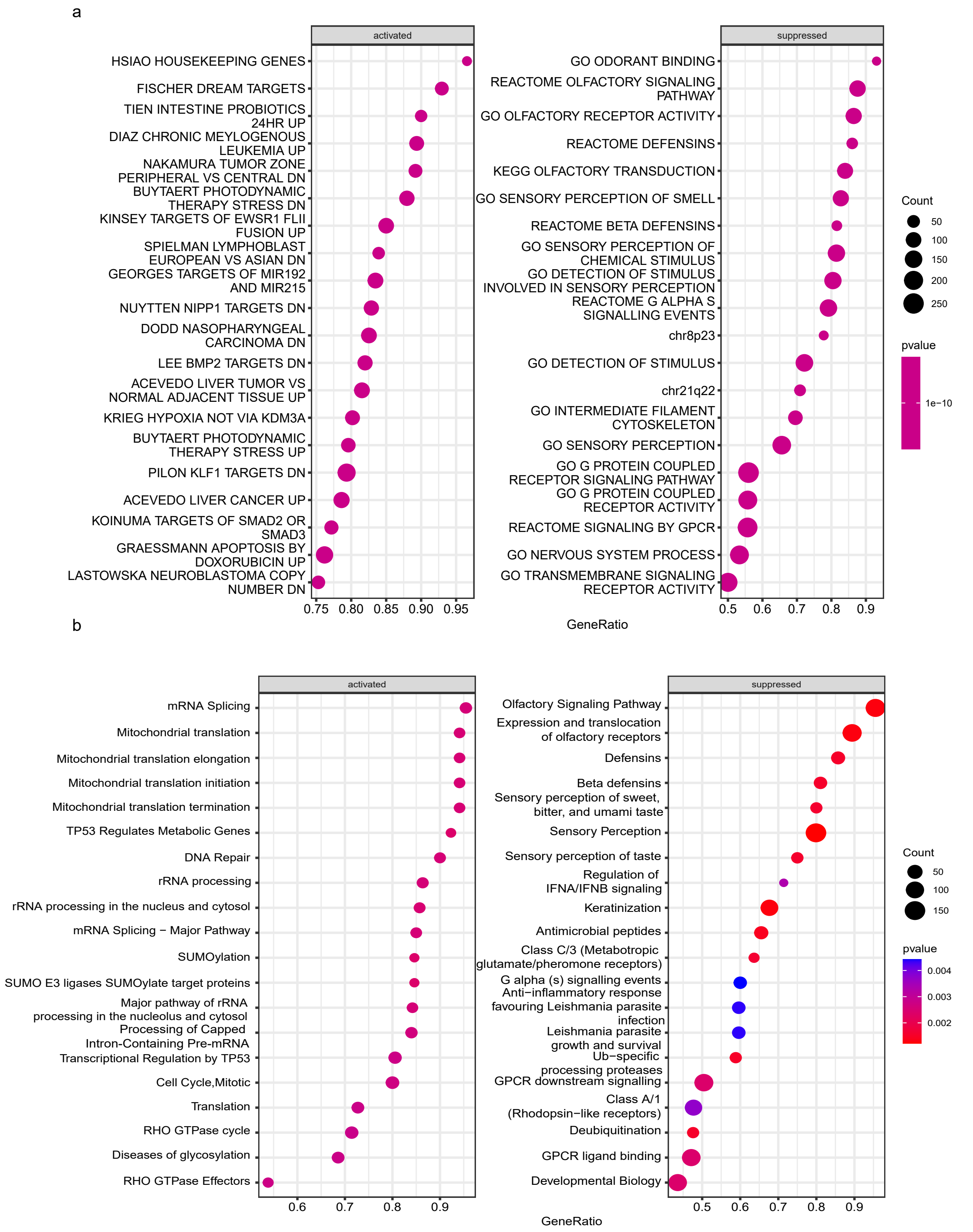

Supplementary Figure 6. Functional enrichment according to Msigdb and Reactome

The bubble plots depicting Msigdb enrichment results (a) and Reactome enrichment results (b) based on differentially expressed genes.

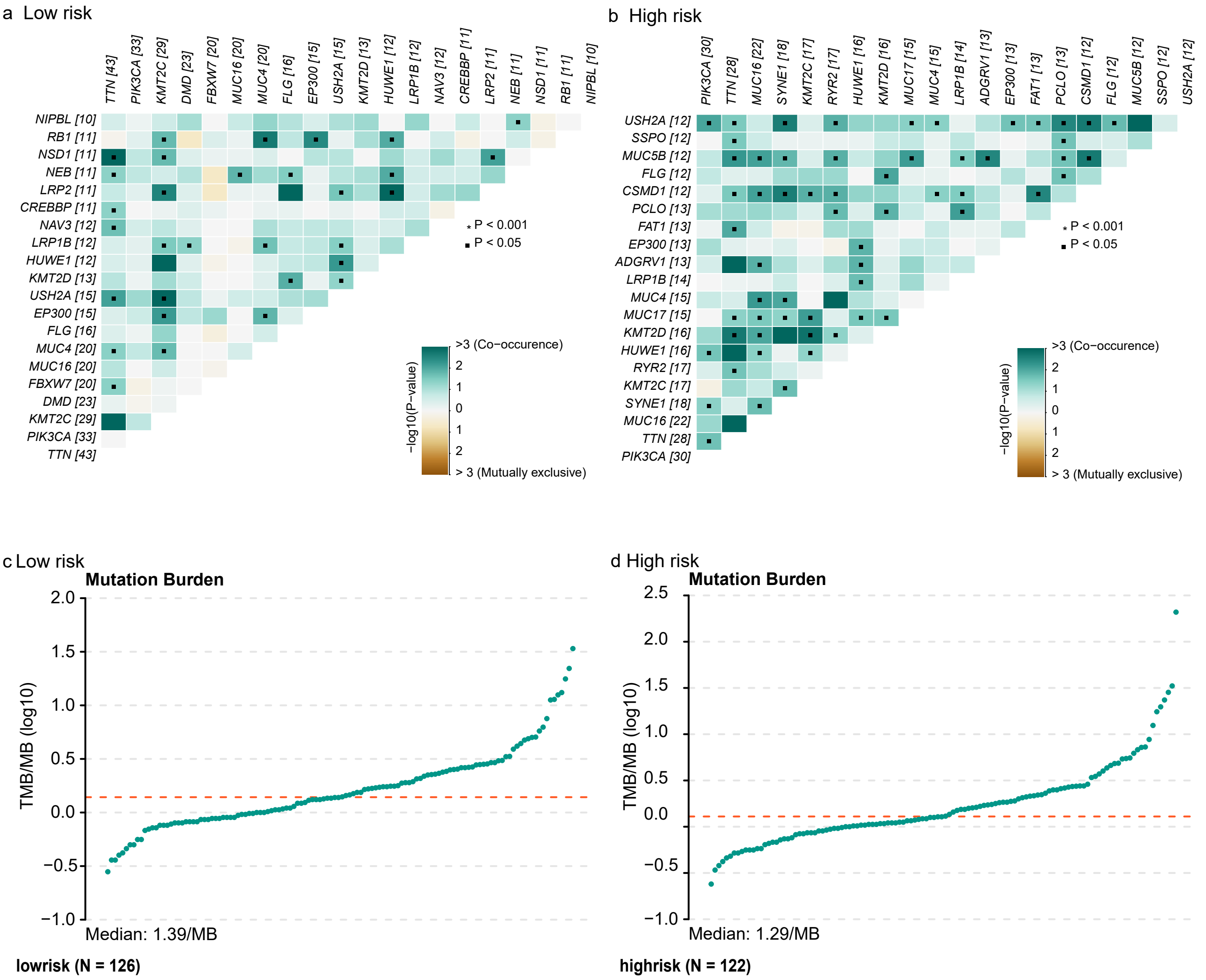

Supplementary Figure 7. The cooccurrence of mutations and tumour mutation burden in CC patients

Mutually exclusive and co-occurring gene pairs in low-risk group (a) and in high-risk group (b) displayed in a triangular matrix. Asterisk stands for $p < 0.001$ and dot for $p < 0.05$. The tumor mutation burden of each patient in low-risk group (c) and in high-risk group (d).

TMB, tumor mutation burden; CC, cervical cancer

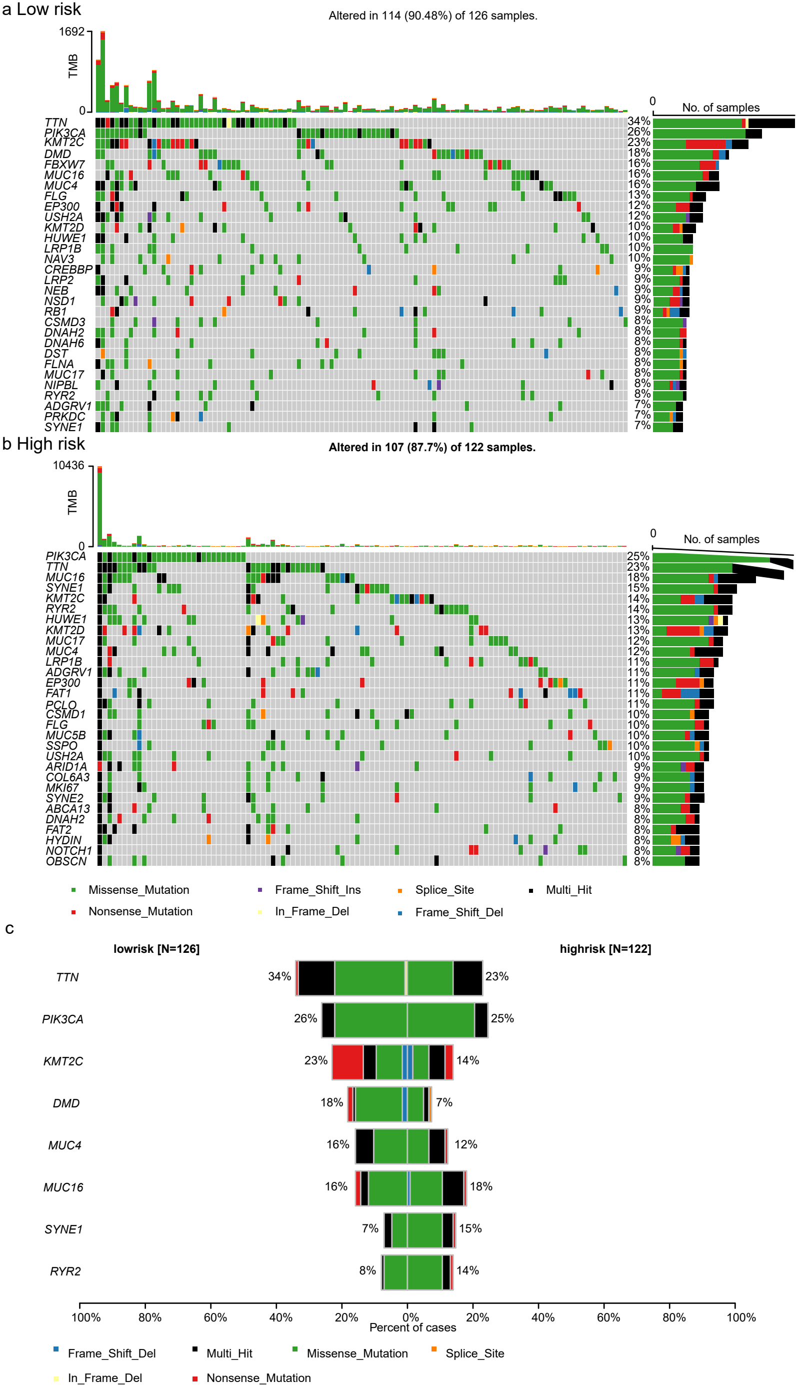

Supplementary Figure 8. The mutation pattern of most mutated genes in CC patients. The oncoplots displaying the somatic mutation landscape of top 30 genes in low-risk group (a) and in high-risk group (b). (c) Comparison on the mutation frequency and type of top 8 genes between the risk groups.

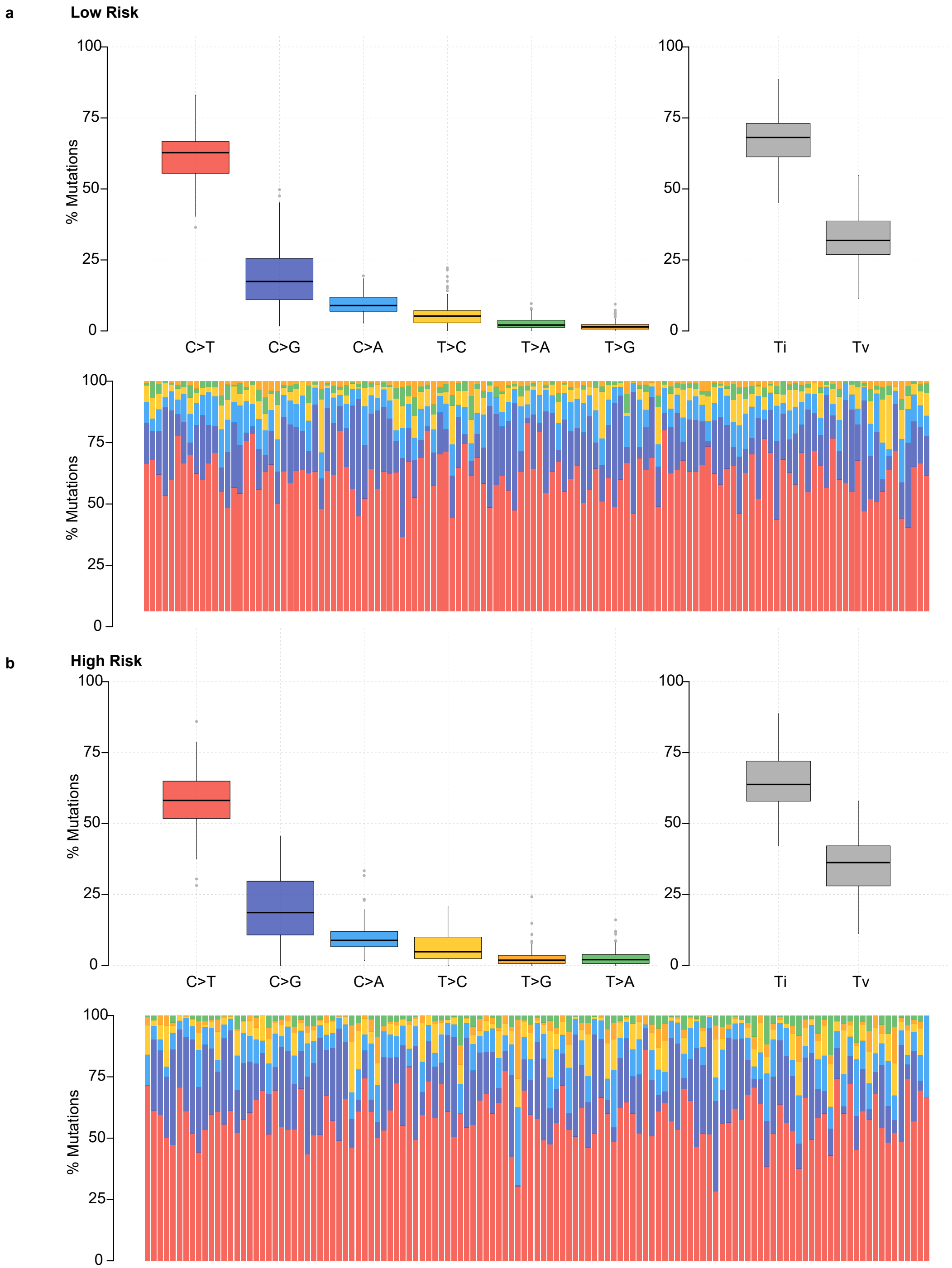

Supplementary Figure 9. Summary of transition and transversion in CC patients. Transition and transversion plot displaying distribution of six conversions in low-risk group (a) and high-risk group (b).

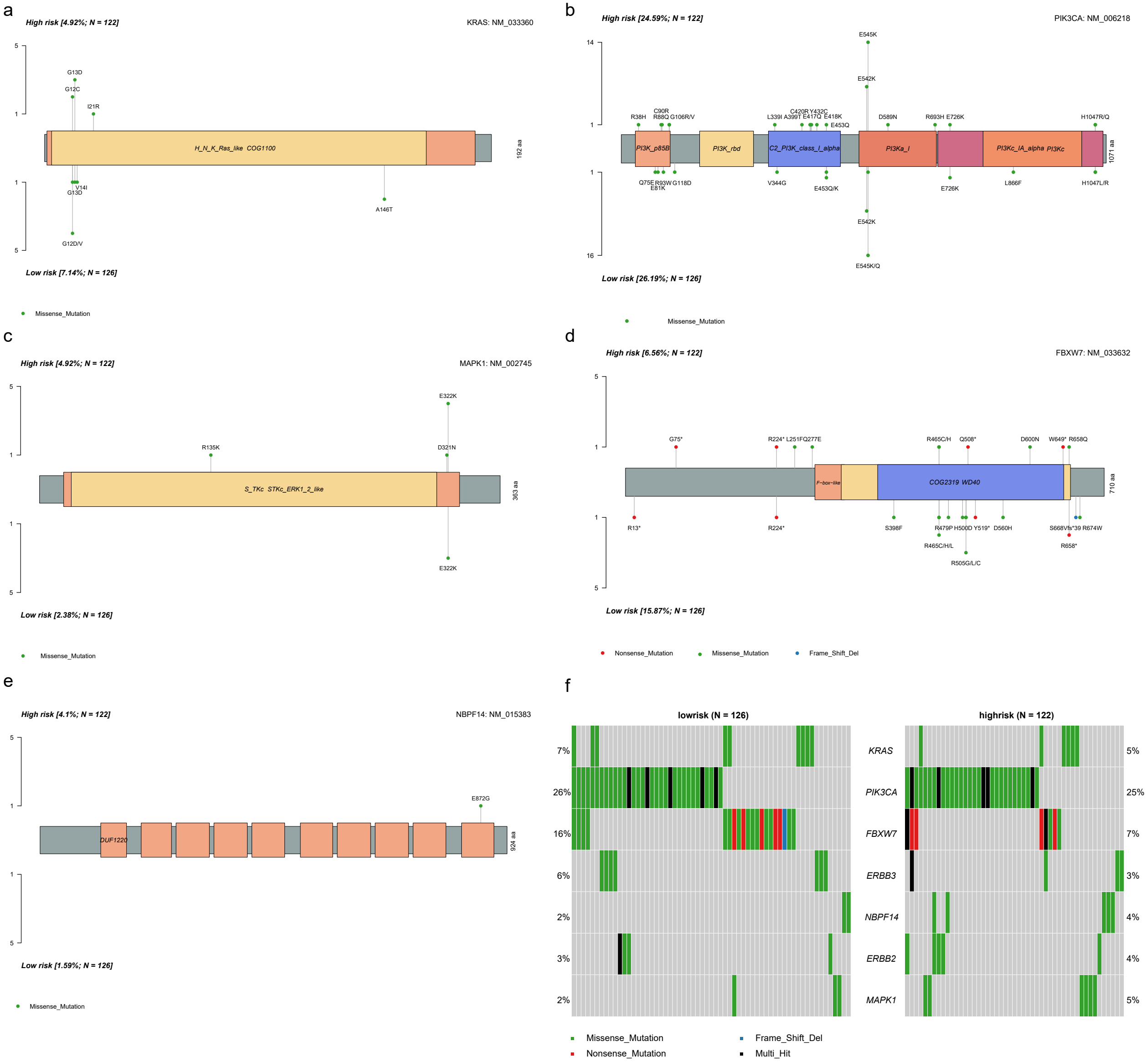

Supplementary Figure 10. The mutatiorhotspots of oncogenic driver genes.

Lollipop plot displaying mutation distribution and protein domains for *KRAS* (a), *PIK3CA* (b), *MAPK1* (c), *FBXW7* (d), and *NBPF14* (e) in different risk groups with labeled recurrent hotspots. (f) The mutation frequency and type of oncogenic driver genes.

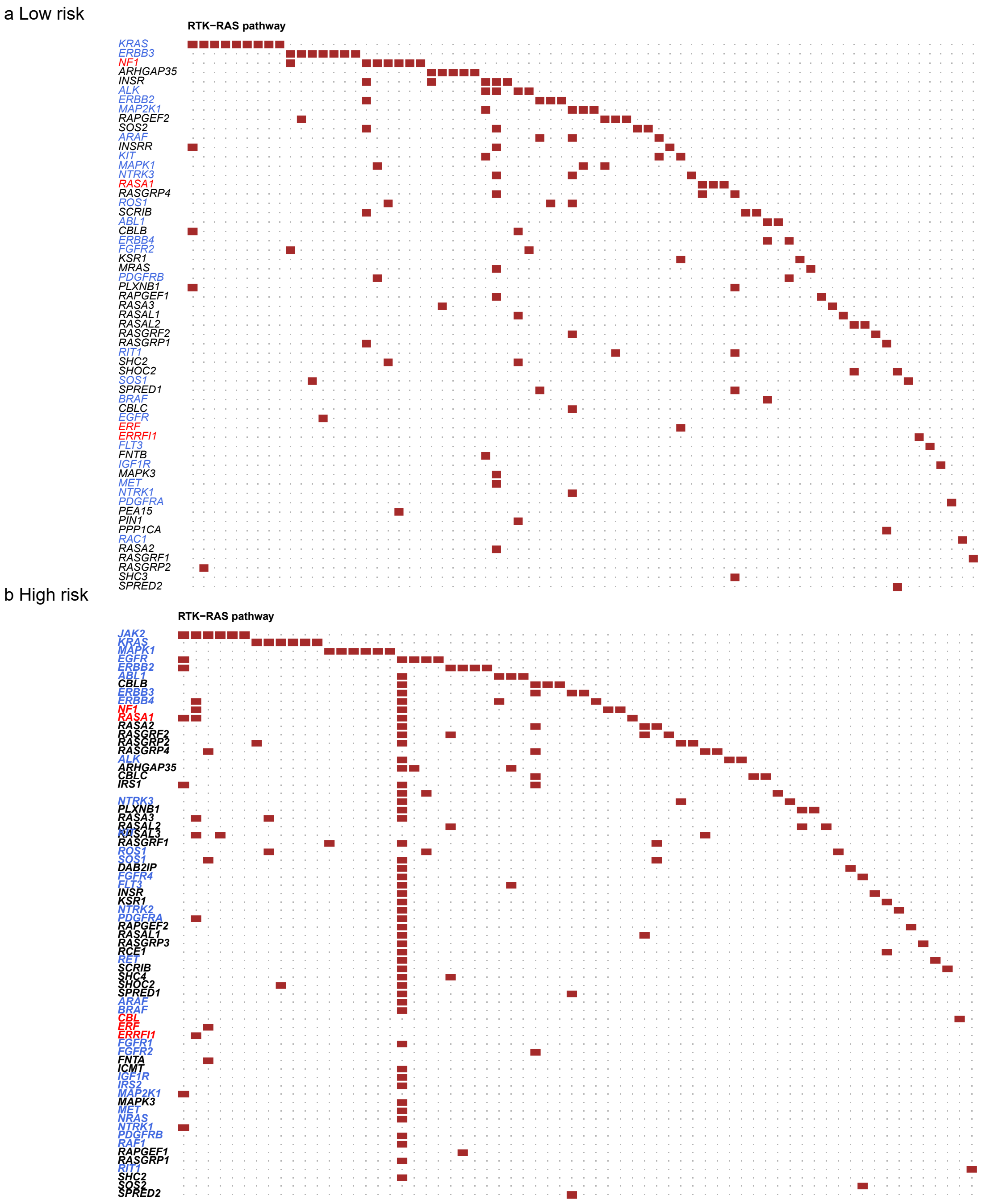

Supplementary Figure 11. The distribution of mutated genes in oncogenic RTK-RAS pathway.

The mutation distribution in oncogenic RTK-RAS pathway in the low-risk group (a) and in the high-risk group (b). Tumor suppressor genes are in red, and oncogenes are in blue.

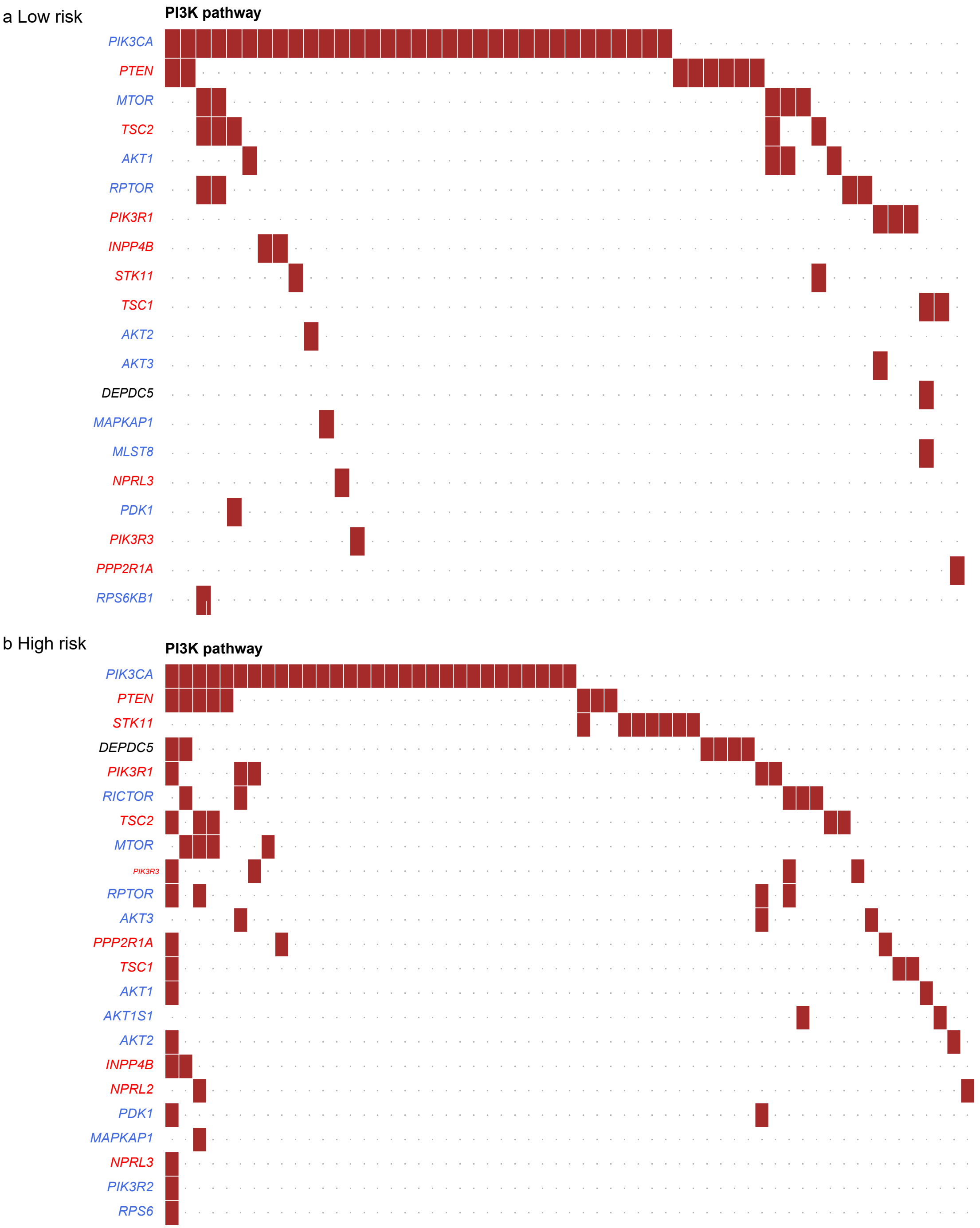

Supplementary Figure 12. The distribution of mutated genes in oncogenic PI3K pathway.

The mutation distribution in oncogenic PI3K pathway in the low-risk group (a) and in the high-risk group (b). Tumor suppressor genes are in red, and oncogenes are in blue.

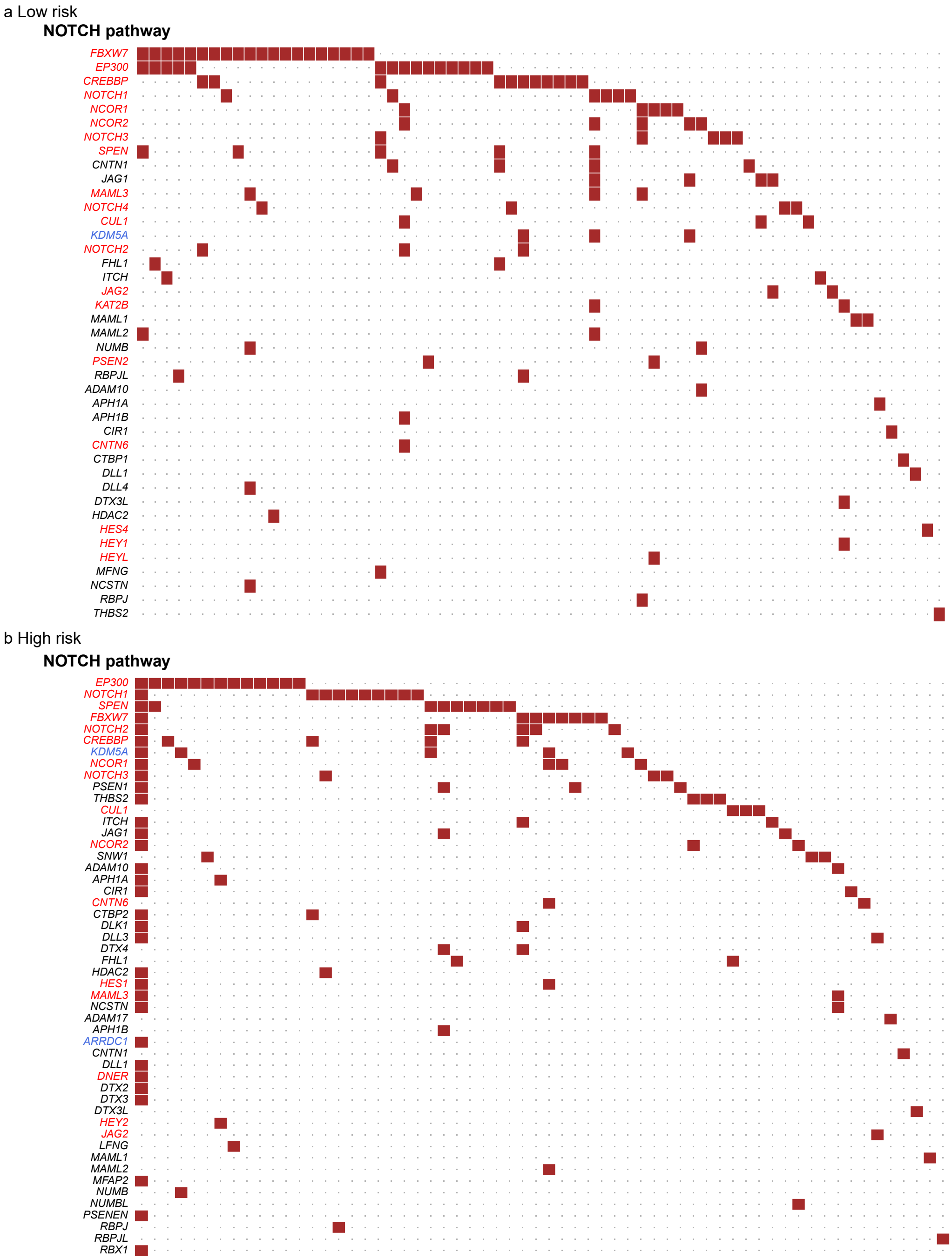

Supplementary Figure 13. The distribution of mutated genes in oncogenic NOTCH pathway.

The mutation distribution in oncogenic NOTCH pathway in the low-risk group (a) and in the high-risk group (b). Tumor suppressor genes are in red, and oncogenes are in blue.

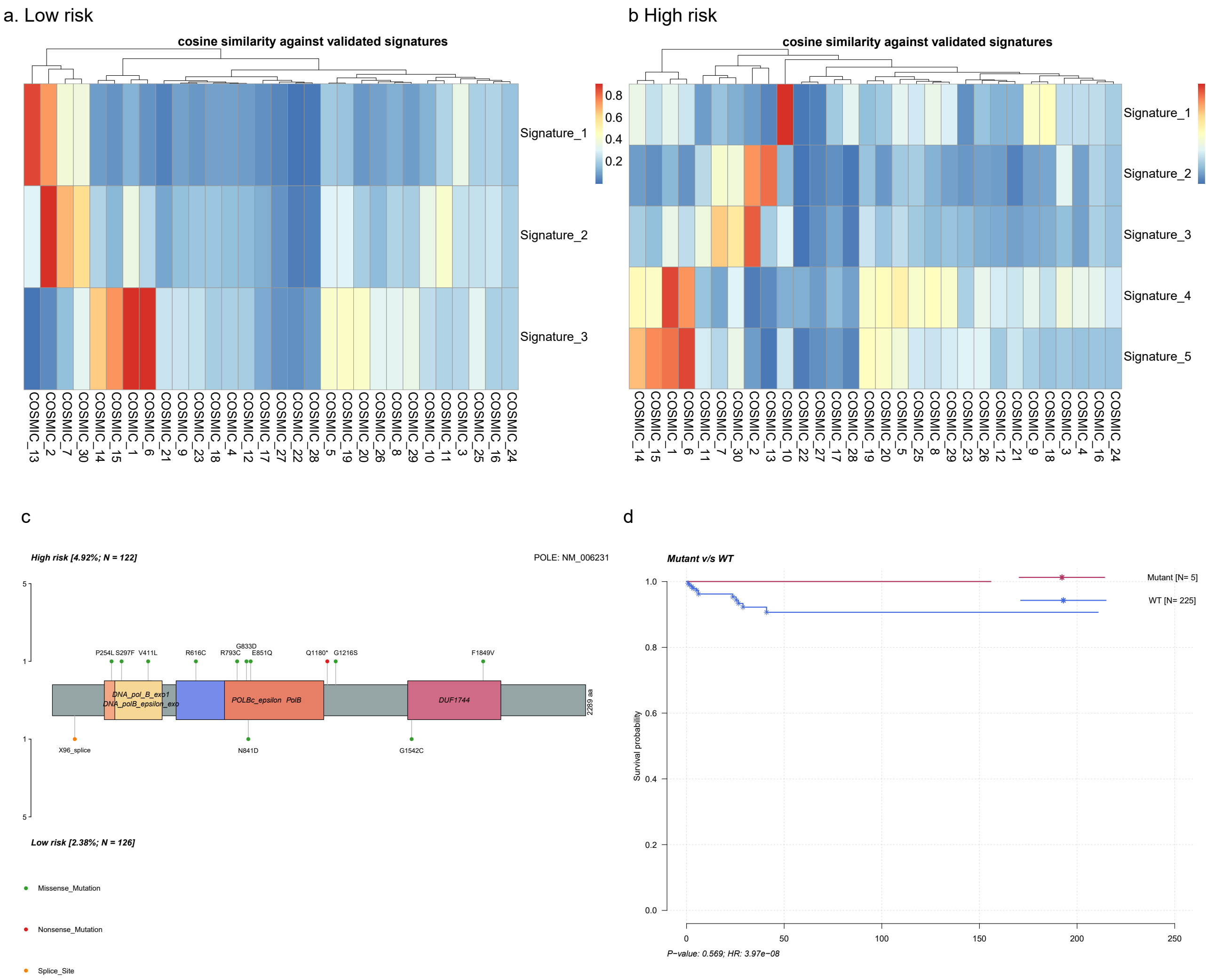

Supplementary Figure 14. Comparison against known signatures and mutation hotspot of *POLE* and their influence on survival.

The colors in the heatmap stands for the cosine similarity generated by comparison of the COSMIC signatures and the signatures detected in the low-risk groups (a) or in the high-risk groups (b). (c) Lollipop plot displaying mutation distribution and protein domains for *POLE* in the different risk groups with labeled recurrent hotspots. (d) The overall survival concerning the mutation status of *POLE*.

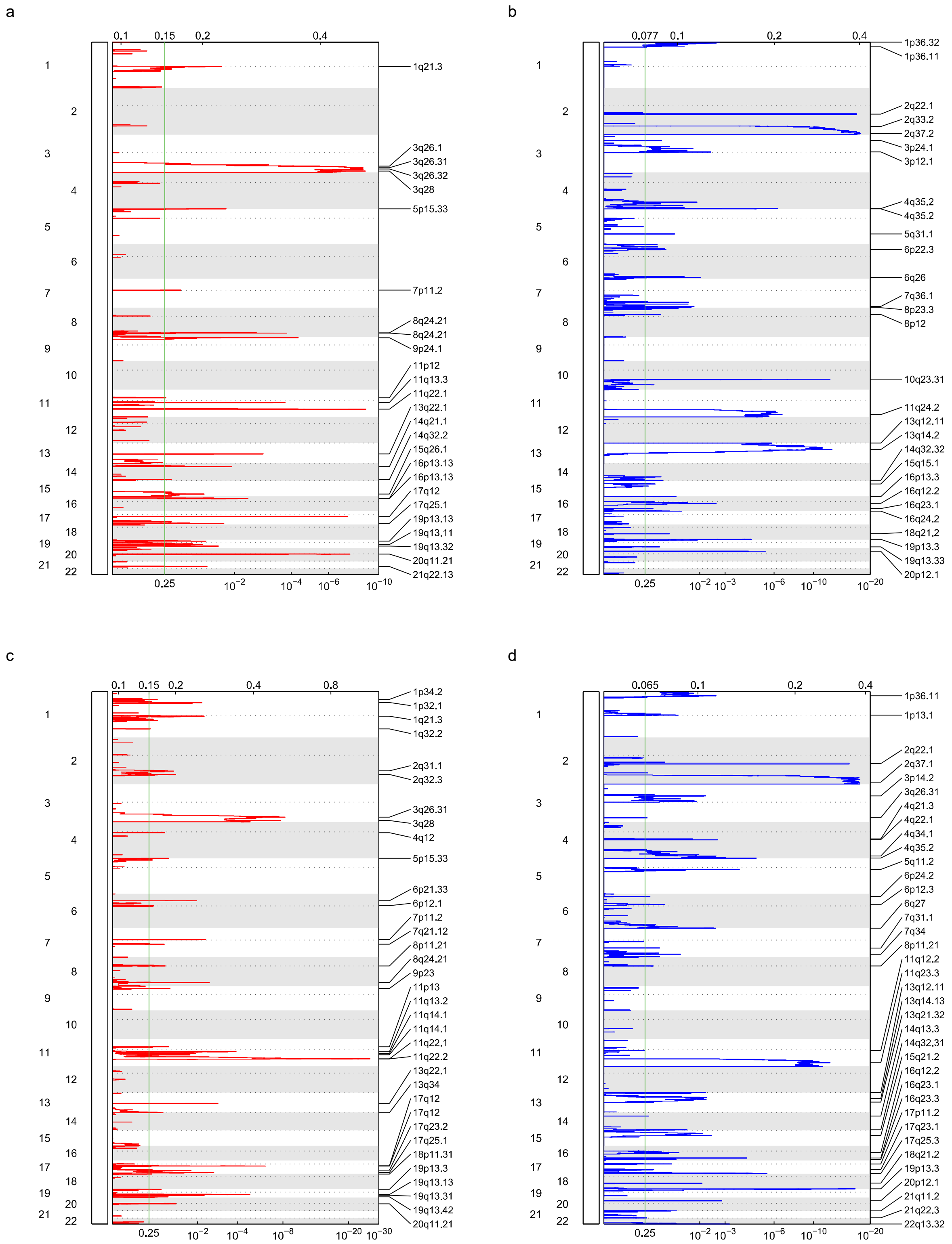

Supplementary Figure 15. Genome-wide distribution of chromatin amplification and deletion.

Genome-wide distribution of chromatin amplification (a) and deletion (b) in low-risk groups. Genome-wide distribution of chromatin amplification (c) and deletion (d) in high-risk groups.

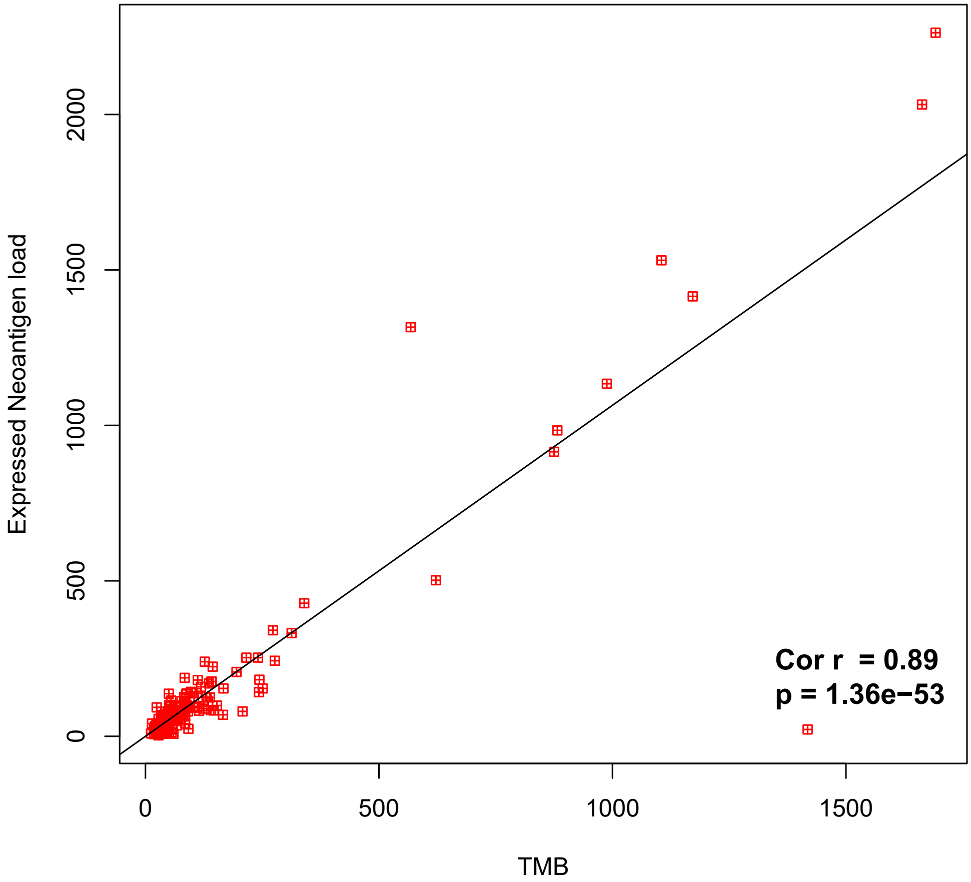

Supplementary Figure 16. Correlation between expressed neoantigen load and TMB.
Correlation between expressed neoantigen load and TMB.
TMB, tumor mutation burden.

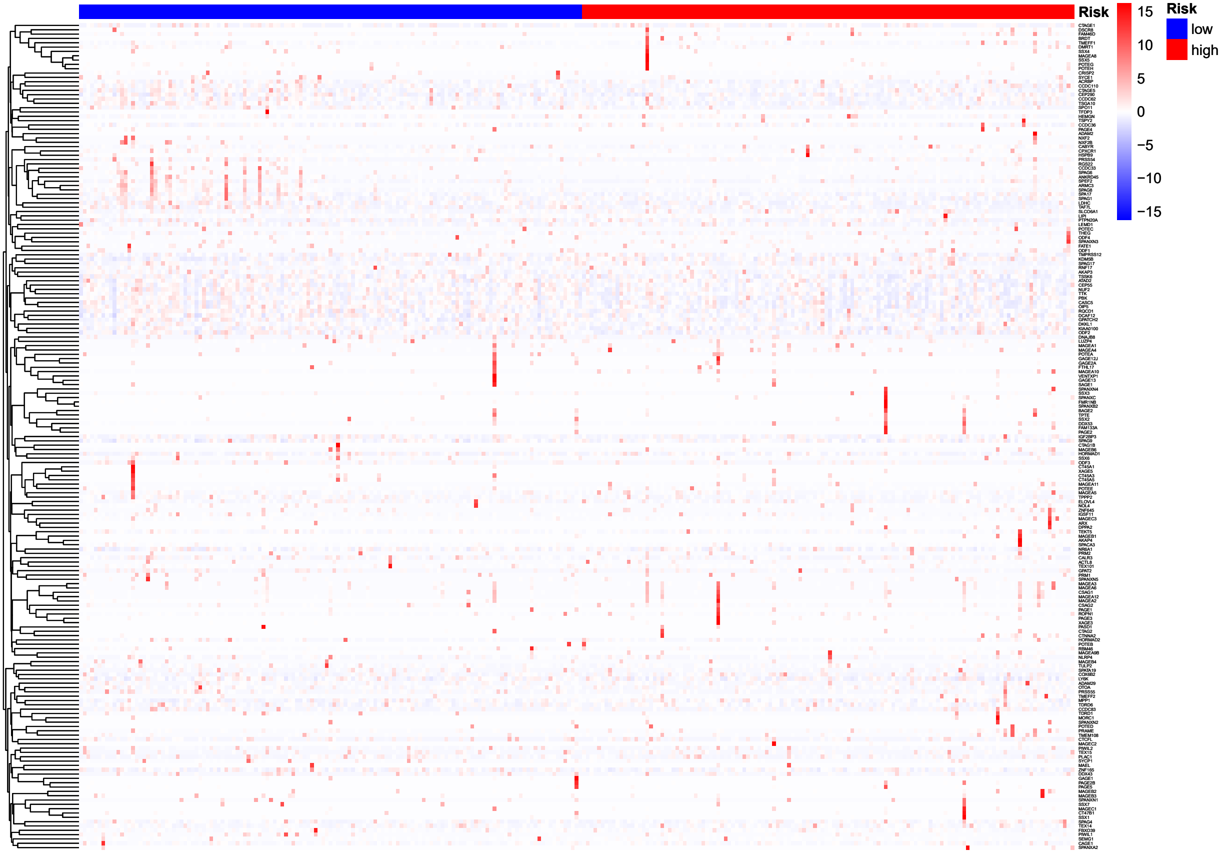

Supplementary Figure 17. Expression levels of cancer germline antigens

The heatmap of the expression levels of cancer germline antigens based on the different risk groups.

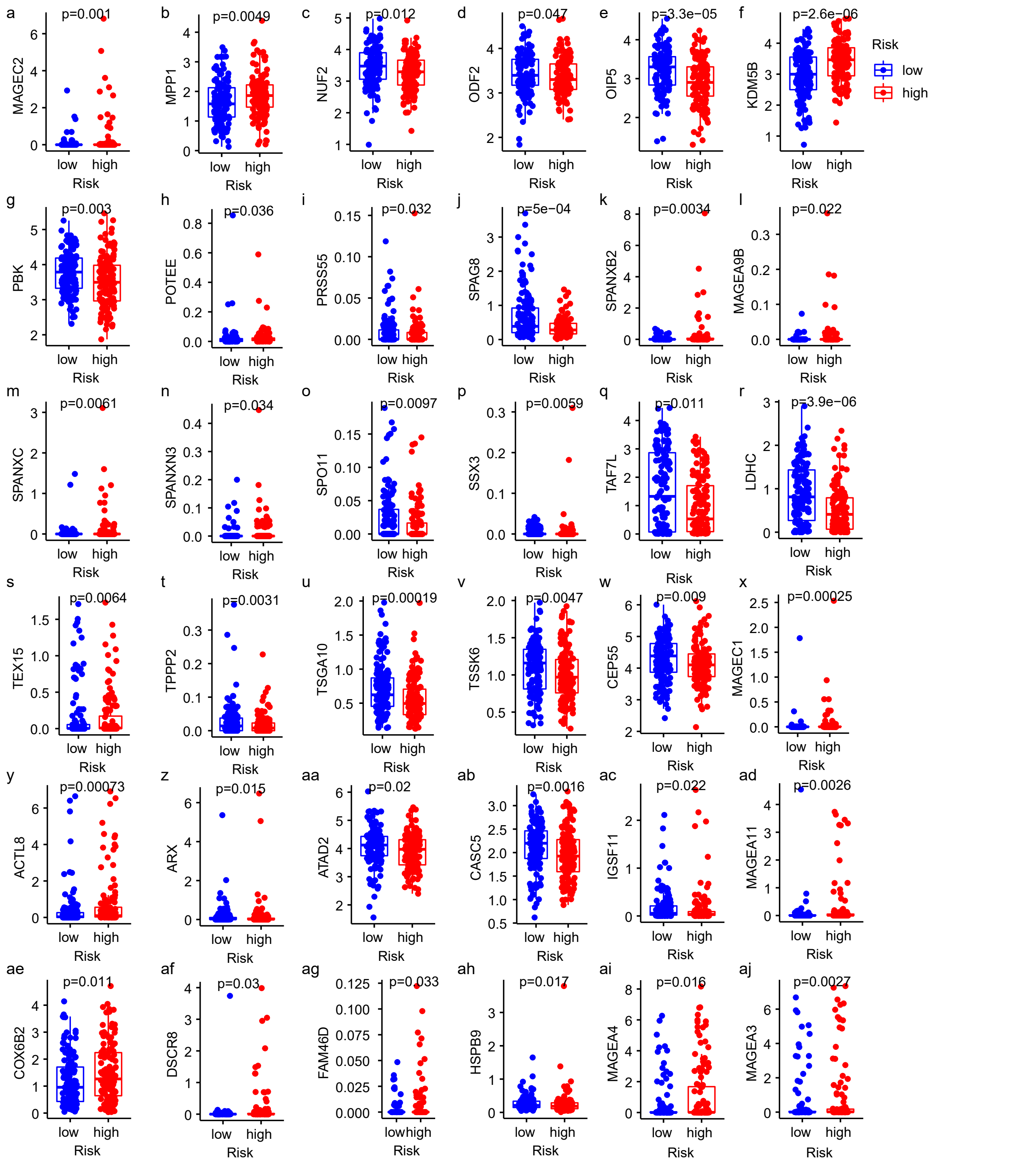

Supplementary Figure 18. Differential expressed cancer germline antigens

The significantly differential expressed cancer germline antigens concerning the different risk groups.

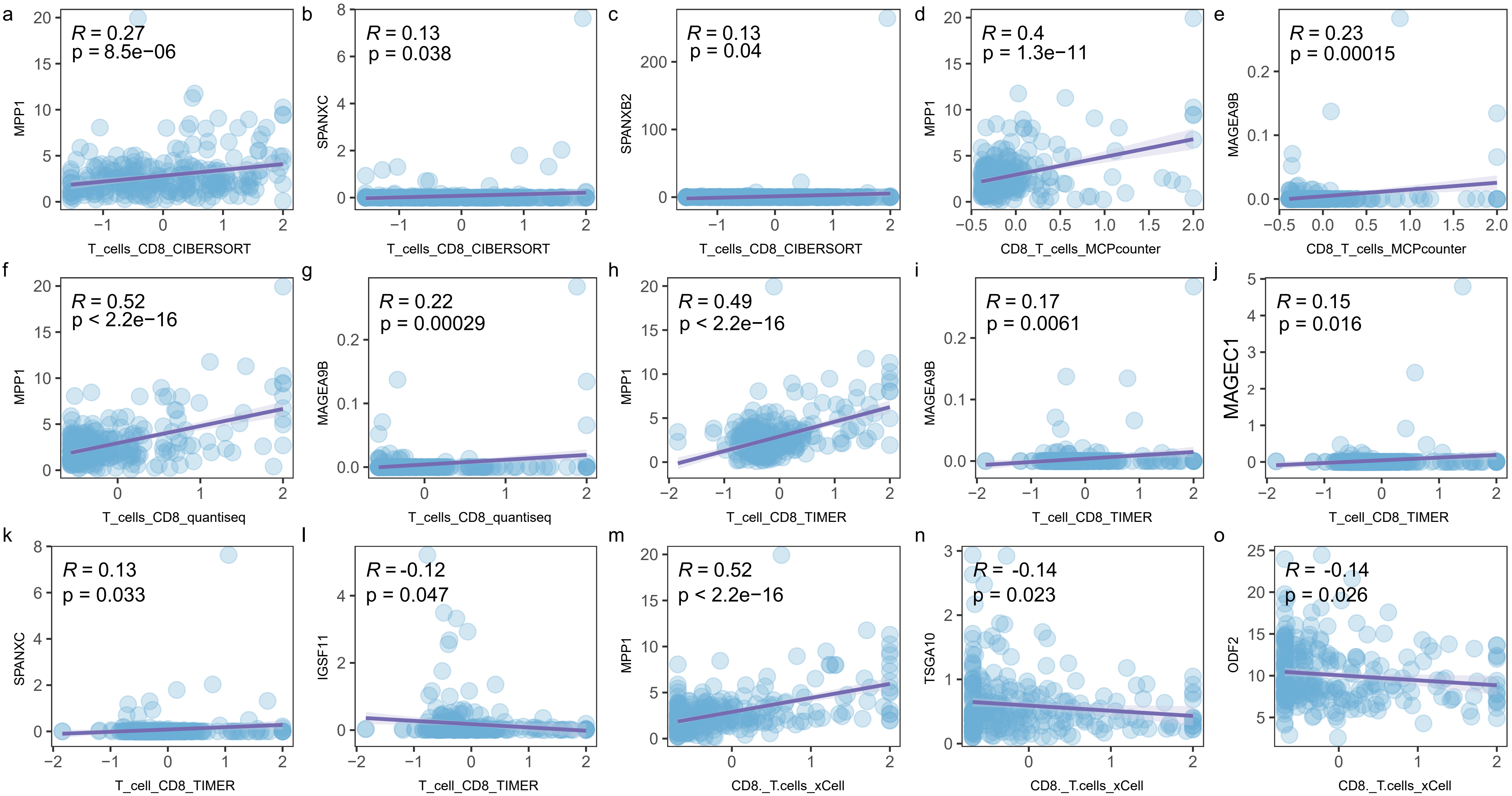

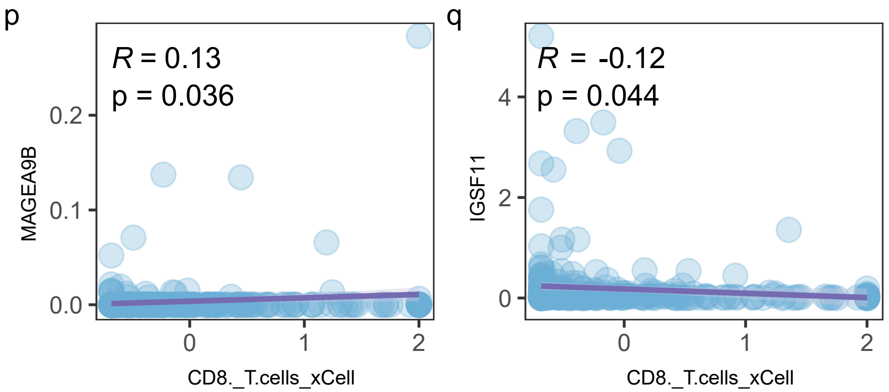

Supplementary Figure 19. Association between cancer germline antigen expression and the CD8 T cell infiltration in the tumour microenvironment.

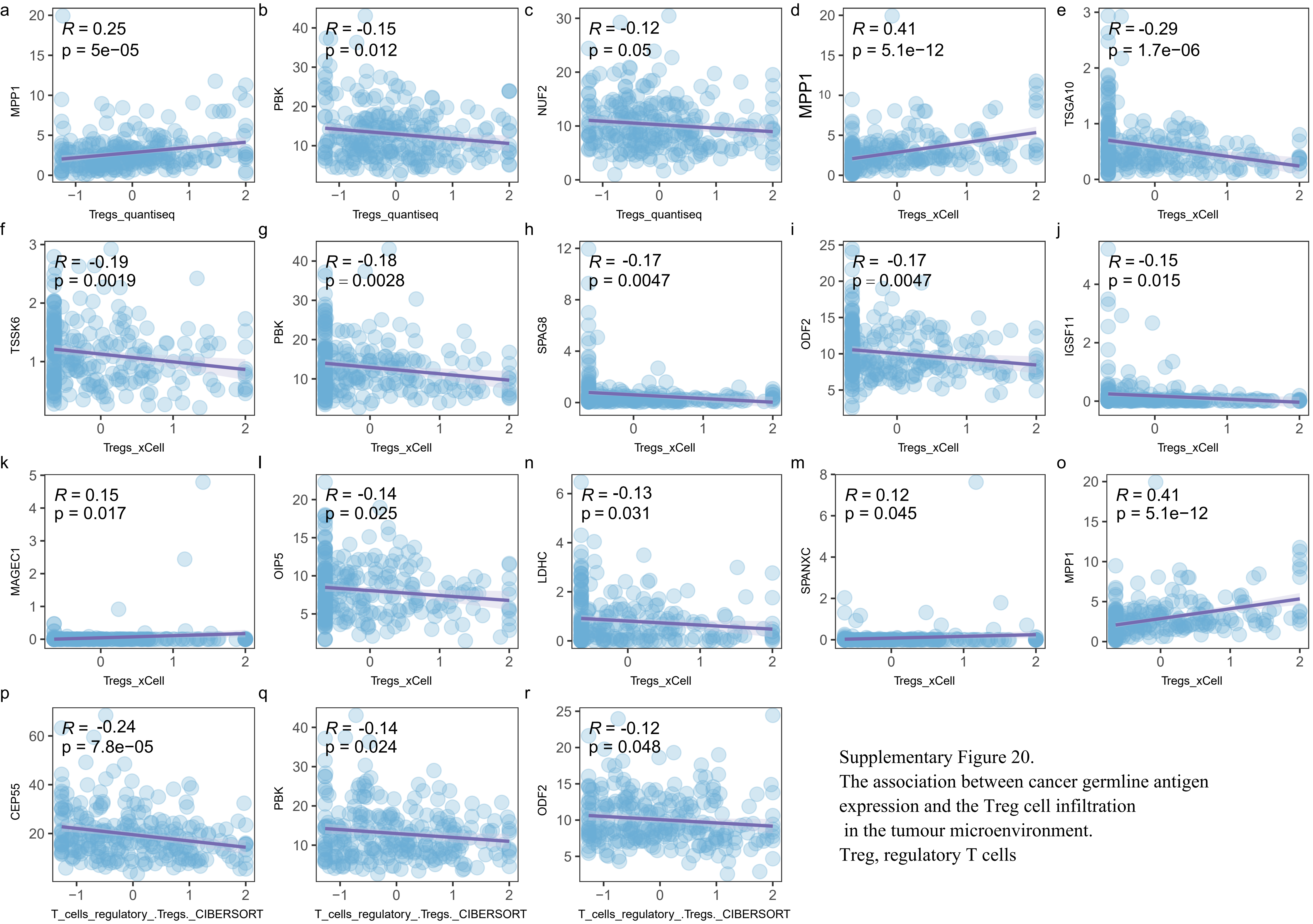

Supplementary Figure 20.
The association between cancer germline antigen expression and the Treg cell infiltration in the tumour microenvironment.
Treg, regulatory T cells

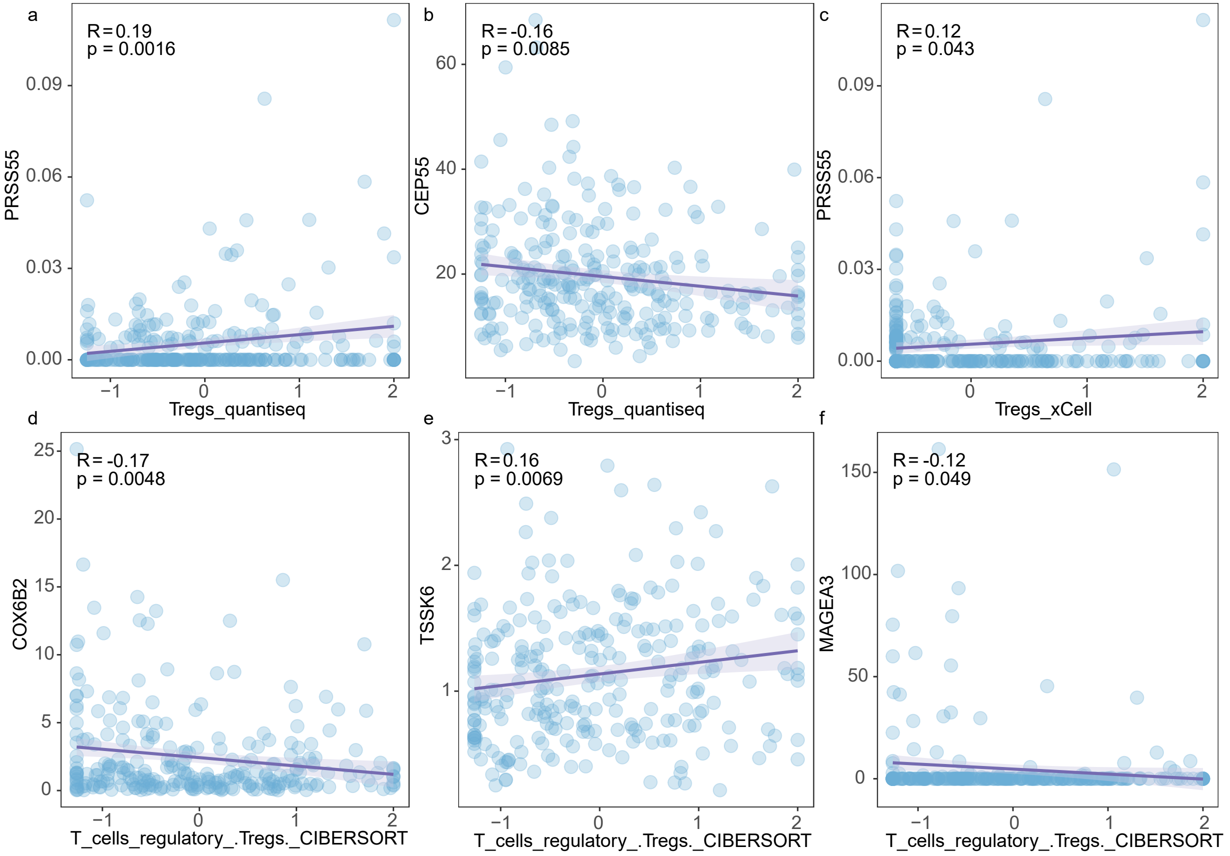

Supplementary Figure 21. The association between cancer germline antigen expression and the Treg cell infiltration in the tumor microenvironment.

Treg, regulatory T cells

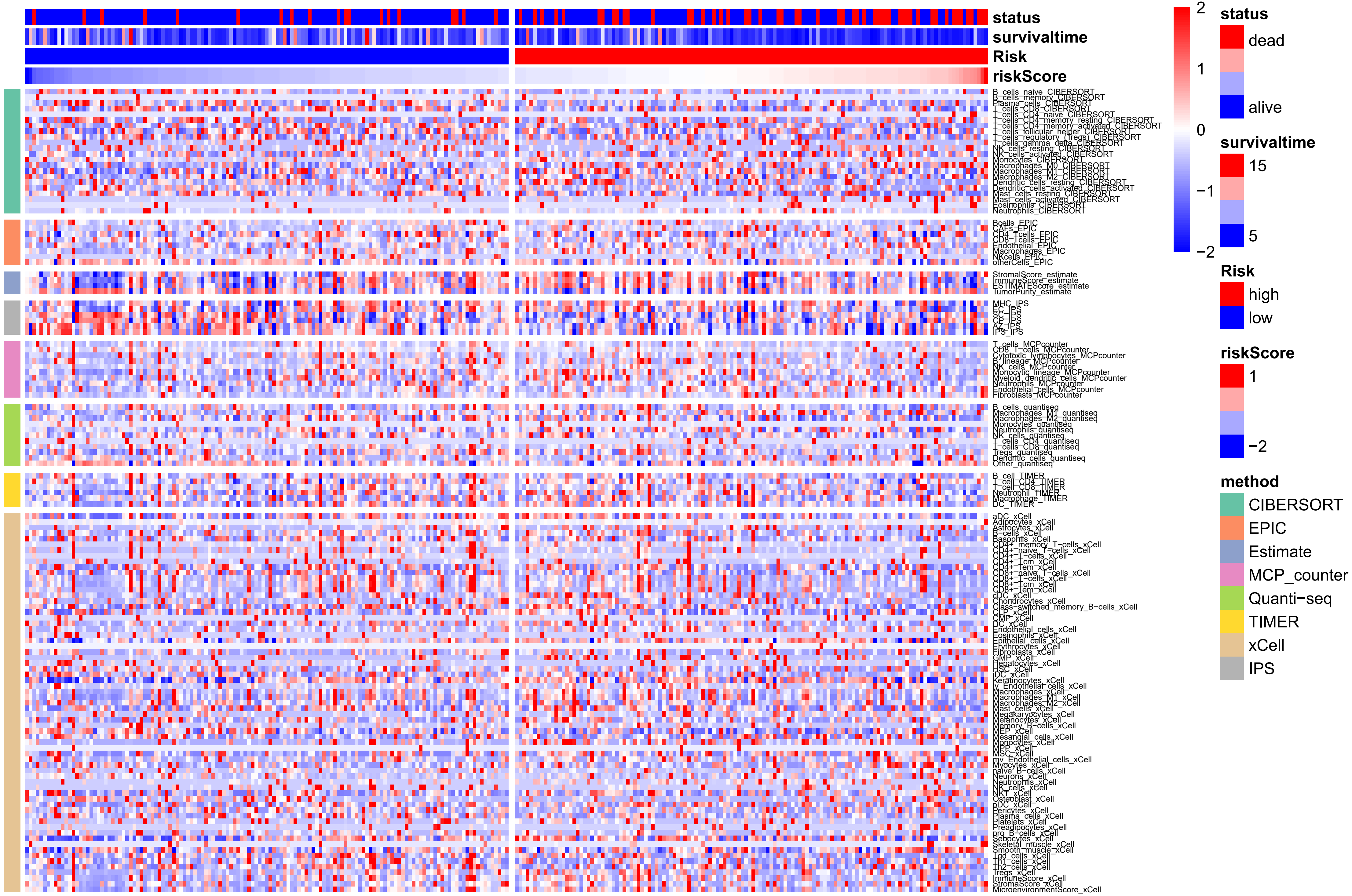

Supplementary Figure 22. Distribution of infiltrating cells

The heatmap of the distribution of infiltrating cells in tumor microenvironment calculated by 8 different algorithms.

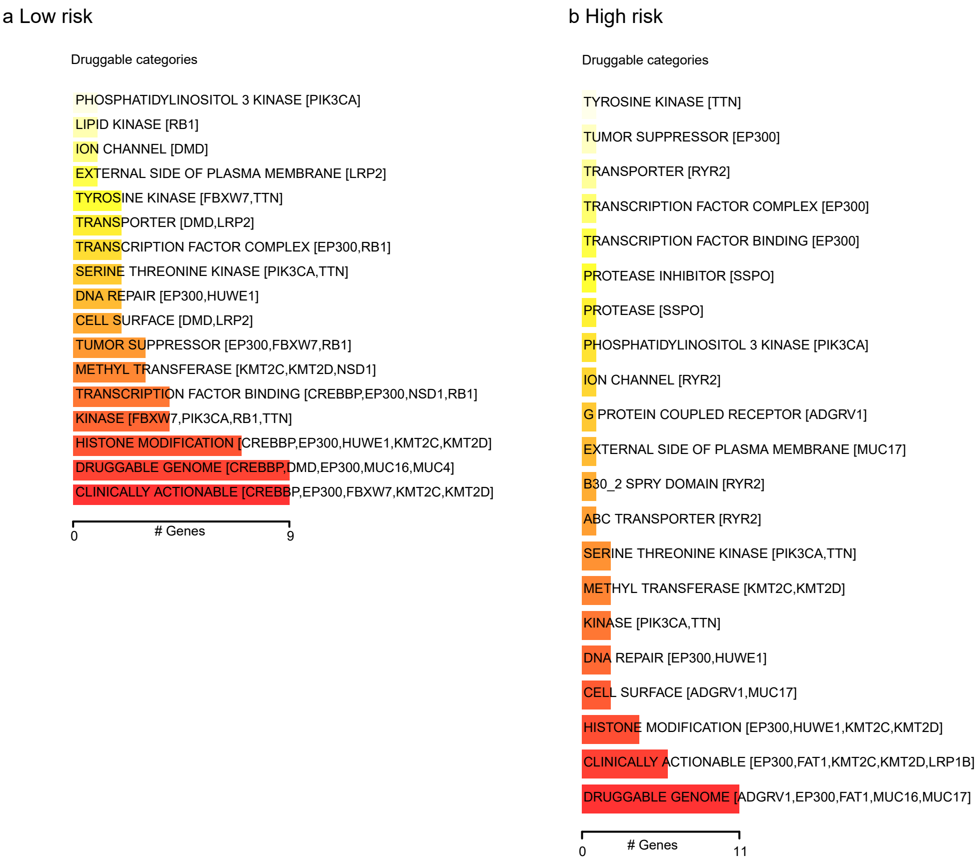

Supplementary Figure 23. Potential drug-target categories

The potential drug-target categories by maftools in the low-risk group (a) and in the high-risk group (b).
