# Supplementary material for: Prognosis and Characterization of Microenvironment in Cervical Cancer Influenced by Fatty Acid Metabolism-Related Genes
Source: J Oncol. 2023 Mar 8;2023:6851036. doi: 10.1155/2023/6851036 (PMC10017219; doi:10.1155/2023/6851036)
Supplement: Supplementary Materials — Supplementary Table 1: fatty acid metabolism-related genes. Supplementary Table 2: the LASSO genes and their coefficient. Supplementary Table 3: the results of PH assumption. Supplementary Table 4: the significant genes in SVM model. Supplementary Table 5: the significant genes in univariate model. Supplementary Table 6: the intersection of significant genes in different models. Supplementary Table 7: the intersection of hub genes by “friends” analysis and “closeness” analysis. Supplementary Table 8: the transcription factors. Supplementary Table 9: the influence of infiltrating cells and risk score on survival by Cox regression. Supplementary Figure 1: construction of FAM-related clinicopathologic nomogram with risk score. Supplementary Figure 2: validation of nomogram with risk group. Supplementary Figure 3: results of PH assumption. Supplementary Figure 4: the expression levels of pivotal enzymes of FAM based on the risk groups. Supplementary Figure 5: functional enrichment according to GO and KEGG. Supplementary Figure 6: functional enrichment according to Msigdb and Reactome. Supplementary Figure 7: the cooccurrence of mutations and tumour mutation burden in CC patients. Supplementary Figure 8: the mutation pattern of most mutated genes in CC patients. Supplementary Figure 9: summary of transition and transversion in CC patients. Supplementary Figure 10: the mutation hotspots of oncogenic driver genes. Supplementary Figure 11: the distribution of mutated genes in oncogenic RTK-RAS pathway. Supplementary Figure 12: the distribution of mutated genes in oncogenic PI3K pathway. Supplementary Figure 13: the distribution of mutated genes in oncogenic NOTCH pathway. Supplementary Figure 14: comparison against known signatures and mutation hotspot of POLE and their influence on survival. Supplementary Figure 15: genome-wide distribution of chromatin amplification and deletion. Supplementary Figure 16: correlation between expressed neoantigen load and TMB. Supplementary [file 6851036.f1.zip › Supplementary Table 1-9 (1).docx]

Supplementary Table 1. Fatty acid metabolism related genes

| FADS2 | RAP1GDS1 | SDHA | ALDH2 | ACSBG1 | HSD17B4 | HACD1 | HSD17B3 | PTGES2 |
| --- | --- | --- | --- | --- | --- | --- | --- | --- |
| DLD | CBR1 | ADIPOR2 | HPGD | CD1D | ERP29 | PTGES | AADAT | PDHA1 |
| ACAA2 | ACSF3 | MAOA | PRDX6 | CD36 | PRKAG2 | ACADVL | ELOVL2 | ALOX15B |
| PON1 | MDH1 | PECR | PPT2 | GPX1 | ENO3 | ACBD4 | CA6 | CEL |
| CYP4F8 | ACBD6 | MORC2 | SDHC | UBE2L6 | CYP2C9 | CYP4A22 | ADH1A | DPEP3 |
| ACBD5 | AWAT1 | ALOXE3 | SLC25A1 | EHHADH | ACACA | ALDH1A1 | ACAA1 | CA4 |
| OLAH | PRKAA2 | ACOT7 | ACOT8 | ABCD1 | ACSL3 | GLUL | ACOT9 | NCAPH2 |
| ADH5 | HACD2 | OSTC | CYP1B1 | CYP4F22 | FADS1 | HMGCS2 | IDH3G | ALOX15 |
| MECR | CPOX | HSD17B11 | ACSM6 | HACD4 | FAAH | CYP2U1 | NTHL1 | MIX23 |
| SDHD | HSPH1 | KMT5A | MID1IP1 | ALDH1B1 | PPARD | HTD2 | SUCLA2 | ADH4 |
| UROD | PTGDS | HADHA | ABCC1 | PTGR1 | DHCR24 | HCCS | FABP2 | GPD1 |
| PHYH | CYP2C8 | ECI1 | CYP2J2 | RXRA | THEM4 | FABP1 | PDHB | PTGR2 |
| ACOXL | NDUFAB1 | CBR3 | MCEE | PTGES3 | CRYZ | PCTP | THEM5 | CPT1B |
| LTA4H | CYP8B1 | ADH1C | HSD17B8 | BLVRA | SUCLG2 | THRSP | ACOX3 | ECH1 |
| CYP1A2 | GCDH | ELOVL3 | PON3 | TDO2 | ACADS | SCD5 | YWHAH | GGT5 |
| TBXAS1 | AMACR | PCCA | ACOT11 | PTGS1 | EPHX1 | ACSBG2 | ACSF2 | ALDH3A1 |
| ME1 | FASN | DPEP1 | GPX2 | GAD2 | AKR1C3 | CYP4F3 | MAPKAPK2 | CBR4 |
| MCAT | ODC1 | ALAD | CIDEA | ELOVL4 | CA2 | RDH11 | AOC3 | GGT1 |
| UROS | CYP2C19 | LDHA | ACADM | ACOT13 | HADHB | ADH6 | ACADSB | ACSL1 |
| ACAT1 | G0S2 | ELOVL1 | CROT | GABARAPL1 | ALOX5AP | NSDHL | FMO1 | ACAT2 |
| ALOX5 | PTS | TECRL | GPX4 | CPT1A | FAAH2 | ECHS1 | GSTZ1 | ACSS1 |
| HACD3 | IDH3B | GRHPR | FH | ETFDH | ACSM3 | PLA2G4A | ACAD10 | HADH |
| ACBD7 | SCP2 | AUH | D2HGDH | SLC22A5 | H2AZ1 | SMS | XIST | ALOX12B |
| ELOVL5 | NUDT7 | ALDOA | VNN1 | CYP4B1 | MMUT | ACO2 | UGDH | HSD17B12 |
| MDH2 | PPT1 | DPEP2 | EPHX2 | ACOT6 | PON2 | PSME1 | ACOT4 | PTGS2 |
| PTPRG | HAO2 | ACOT2 | SLC27A3 | SERINC1 | DLST | HSD17B10 | ACLY | TECR |
| HMGCS1 | ECI2 | BPHL | METAP1 | APEX1 | ELOVL6 | MIF | RETSAT | NUDT19 |
| HPGDS | HSDL2 | ADSL | PCCB | ACSL5 | SCD | RDH16 | PRXL2B | DECR2 |
| SLC25A20 | ACOX1 | ACSL6 | ACSL4 | AQP7 | IL4I1 | ACADL | PTGIS | ALDH3A2 |
| REEP6 | HMGCL | ALDH7A1 | ACAD11 | INMT | ACACB | CPT1C | CYP4F11 | ADH1B |
| ACOX2 | DECR1 | ENO2 | CYP4F2 | IDI1 | ALDH9A1 | HIBCH | LTC4S | ELOVL7 |
| ALOX12 | MMAA | NBN | LGALS1 | GAPDHS | MGLL | SLC27A2 | PPARA | HSP90AA1 |
| MLYCD | CYP1A1 | PCBD1 | SLC25A17 | BCKDHB | DBI | SUCLG1 | CPT2 | ACOT12 |
| HACL1 | ADH7 | GPD2 | TP53INP2 | IDH1 | S100A10 | CRAT | HSD17B7 | CYP4A11 |
| ACOT1 | PRKAB2 | BMPR1B |  |  |  |  |  |  |

Supplementary Table 2. The Lasso genes and their coefficient

| Gene | coefficient |
| --- | --- |
| CD1D | 0.00371234434016065 |
| CEL | -0.000285270543834027 |
| NCAPH2 | -0.0232634228222337 |
| SDHD | -0.00544300136801038 |
| ADH4 | 0.279783059240964 |
| HCCS | -0.00910669177193145 |
| THRSP | 0.422537143464369 |
| GCDH | -0.0245185766675298 |
| NUDT7 | -0.0352303680462543 |
| DPEP2 | -0.0392703416411635 |
| SERINC1 | 0.00209936353264552 |
| MIF | 0.00479589192558066 |
| ELOVL7 | -0.0877437108857868 |
| CYP1A1 | 0.601682881902629 |

Supplementary Table 3. Results of PH assumption

|  | chisq | df | p |
| --- | --- | --- | --- |
| CD1D | 0.18 | 1 | 0.67 |
| CEL | 1.19 | 1 | 0.27 |
| NCAPH2 | 2.07 | 1 | 0.15 |
| SDHD | 1.29 | 1 | 0.26 |
| ADH4 | 0.0599 | 1 | 0.81 |
| HCCS | 0.0895 | 1 | 0.76 |
| THRSP | 1.59 | 1 | 0.21 |
| GCDH | 1.58 | 1 | 0.21 |
| NUDT7 | 0.732 | 1 | 0.39 |
| DPEP2 | 0.00075 | 1 | 0.98 |
| SERINC1 | 3.97 | 1 | 0.05 |
| MIF | 5.16 | 1 | 0.02 |
| ELOVL7 | 5.67 | 1 | 0.017 |
| CYP1A1 | 0.0162 | 1 | 0.9 |
|  |  |  |  |
| MIF |  |  |  |
| high-risk | 1.95 | 1 | 0.16 |
| low-risk | 0.852 | 1 | 0.36 |
| ELOVL7 |  |  |  |
| high-risk | 11.9 | 1 | 0.00057 |
| low-risk | 0.0223 | 1 | 0.88 |
|  |  |  |  |
| Risk score |  |  |  |
| Univariate Cox analysis | 0.183 | 1 | 0.67 |
| Multivariate Cox analysis | 0.261134 | 1 | 0.609 |
|  |  |  |  |
| Risk |  |  |  |
| Univariate Cox analysis | 2.12 | 1 | 0.15 |
| Multivariate Cox analysis | 0.9678 | 1 | 0.325 |

Supplementary Table 4. The significant genes in SVM model

| SDHA | CYP2J2 | ABCC1 | DLD | CEL | PTGES | PTGR2 |
| --- | --- | --- | --- | --- | --- | --- |

Supplementary Table 5. The significant genes in univariate model

| CD1D | AADAT | NCAPH2 | IDH3G | SDHD | ADH4 | ACOXL | THRSP | ACOX3 |
| --- | --- | --- | --- | --- | --- | --- | --- | --- |
| GCDH | ELOVL3 | PCCA | GAD2 | CIDEA | CA2 | G0S2 | CPT1A | ACSS1 |
| NUDT7 | PTGS2 | SERINC1 | HSD17B10 | MIF | ADH1B | ELOVL7 | SUCLG1 |  |

Supplementary Table 6. The intersection of significant genes in different models

| Only Lasso | Only unicox | Only rf | Only svm | lasso AND unicox NOT rf NOT svm | lasso AND rf NOT unicox NOT svm | lasso AND svm NOT univariate NOT rf | unicox AND rf NOT lasso NOT svm | lasso AND unicox AND rf NOT svm |
| --- | --- | --- | --- | --- | --- | --- | --- | --- |
| ALOXE3 | CD1D | FASN | SDHA | SDHD | CYP1A1 | CEL | CA2 | NCAPH2 |
| CCS | AADAT | CYP4F22 | CYP2J2 | THRSP |  |  | IDH3G | ADH4 |
|  | ACOXL | ALDH1A1 | ABCC1 | ACOX3 |  |  | ACSS1 | NUDT7 |
|  | ELOVL3 | DHCR24 | DLD | GCDH |  |  |  | SERINC1 |
|  | PCCA | PDHA1 | PTGES | HSD17B10 |  |  |  | ELOVL7 |
|  | GAD2 | DPEP2 | PTGR2 | MIF |  |  |  |  |
|  | CIDEA | ECHS1 |  |  |  |  |  |  |
|  | G0S2 | GPD1 |  |  |  |  |  |  |
|  | CPT1A | ME1 |  |  |  |  |  |  |
|  | PTGS2 | IDI1 |  |  |  |  |  |  |
|  | ADH1B | PCCB |  |  |  |  |  |  |
|  | SUCLG1 | PPARD |  |  |  |  |  |  |
|  |  | SUCLG2 |  |  |  |  |  |  |
|  |  | MDH1 |  |  |  |  |  |  |
|  |  | GLUL |  |  |  |  |  |  |
|  |  | ACLY |  |  |  |  |  |  |
|  |  | ALOX15 |  |  |  |  |  |  |
|  |  | FADS2 |  |  |  |  |  |  |
|  |  | ACAT1 |  |  |  |  |  |  |
|  |  | FADS1 |  |  |  |  |  |  |
|  |  | HSD17B3 |  |  |  |  |  |  |

SVM, support vector machine; unicox, univariate cox regression; rf, random forest

Supplementary Table 7. The intersection of hub genes by “friends” analysis and “closeness” analysis

| Only friends | Only closeness | friends AND closeness |
| --- | --- | --- |
| ZBED9 | CRCT1 | KLF17 |
| CDA | RPRML | RHOXF1 |
| PLA2G3 | IGFL1 | DLX3 |
| KCNG3 | SOWAHA | SOX2 |
| CYP2A13 | TMEM270 | LHX5 |
| SSTR5 | DEGS2 | ACTL8 |
| PCDHGB1 |  | IRX6 |
| COX6A2 |  | ALX1 |
| HMSDD |  | HOXC12 |
| EGS2 |  | FOXJ1 |
|  |  | SCGB2A1 |
|  |  | BASP1 |
|  |  | EREG |
|  |  | SOX21 |
|  |  | IL1A |
|  |  | HMX2 |
|  |  | SP9 |
|  |  | CASP14 |
|  |  | CLDN3 |
|  |  | BMP3 |
|  |  | LBP |
|  |  | HAS2 |
|  |  | AQP5 |
|  |  | CST6 |
|  |  | CDK5R2 |
|  |  | PAPPA |
|  |  | NCCRP1 |
|  |  | ENHO |
|  |  | ALPP |
|  |  | KRT6B |
|  |  | CAMP |
|  |  | SLC6A17 |
|  |  | LRRN4 |
|  |  | HEPHL1 |

Supplementary Table 8. The transcription factors

| ADNP | AFF4 | AR | ARID3A | ARNT | ARNTL | ASCL1 | ASH2L | ATF1 |
| --- | --- | --- | --- | --- | --- | --- | --- | --- |
| ATF2 | ATF3 | ATF4 | ATF7 | BACH1 | BACH2 | BATF | BCL11A | BCL3 |
| BCL6 | BDP1 | BHLHE40 | BMI1 | BRCA1 | BRD1 | BRD2 | BRD3 | BRD4 |
| BRF1 | BRF2 | C17orf96 | CBFB | CBX2 | CBX3 | CBX5 | CBX7 | CBX8 |
| CDK2 | CDK7 | CDK8 | CDK9 | CDX2 | CEBPA | CEBPB | CENPA | CHD1 |
| CHD2 | CHD7 | CHD8 | CIITA | CPSF3L | CREBBP | CTNNB1 | CUX1 | DNMT1 |
| DNMT3A | DYRK1A | E2F1 | E2F3 | E2F4 | E2F6 | E2F7 | EBF1 | EED |
| EGR1 | EGR2 | EHF | EHMT2 | ELF1 | ELF5 | ELK1 | ELK4 | ELL2 |
| EMX1 | EOMES | EP400 | EPAS1 | EPO | ERCC6 | ERG | ESR1 | ESRRA |
| ETS1 | ETV1 | EZH1 | EZH2 | FLI1 | FOS | FOSL1 | FOSL2 | FOXA1 |
| FOXA2 | FOXK1 | FOXM1 | FOXO1 | FOXO3 | FOXP1 | FOXP2 | FOXP3 | GABPA |
| GATA2 | GATA3 | GATA4 | GATA6 | GATAD1 | GREB1 | GRHL2 | GTF2B | GTF2F1 |
| GTF2I | H2AFX | HCFC1 | HDAC1 | HDAC2 | HDAC3 | HDAC6 | HEY1 | HIF1A |
| HIRA | HNF1B | HNF4A | HNF4G | HOXA9 | HOXB13 | HOXB7 | HOXC11 | HOXC9 |
| HSF1 | HSF2 | IKZF1 | IRF1 | IRF3 | IRF4 | IRF5 | JARID2 | JMJD1C |
| JMJD6 | JUN | JUNB | JUND | KAT2B | KAT5 | KDM1A | KDM2B | KDM3A |
| KDM4C | KDM5A | KDM5B | KDM5C | KDM6B | KLF11 | KLF4 | KLF5 | LEF1 |
| LHX2 | LIN9 | LMNA | LMNB1 | LMO2 | LYL1 | MAF | MAFF | MAFK |
| MAX | MAZ | MBD2 | MBD3 | MECP2 | MED12 | MEF2A | MEF2B | MEF2C |
| MEIS1 | MITF | MXI1 | MYB | MYBL2 | MYC | MYH11 | NANOG | NCAPG |
| NCOR2 | NFATC1 | NFE2 | NFIC | NFYA | NFYB | NIPBL | NOTCH1 | NR1H2 |
| NR2C2 | NR2F1 | NR2F2 | NR3C1 | NR4A1 | NR5A2 | NRF1 | OGT | PAF1 |
| PAX3 | PAX5 | PAX6 | PBX1 | PBX3 | PDX1 | PHF8 | PIAS1 | PML |
| POLR2B | POLR3A | POLR3D | POLR3G | POU2F1 | POU5F1 | PPARD | PPARG | PRDM1 |
| PRKDC | RAG1 | RARA | RARG | RB1 | RBBP5 | RBL2 | RBP2 | RBPJ |
| RCOR1 | RELA | RFX2 | RFX5 | RING1 | RNF2 | RUNX1 | RUNX1T1 | RXRA |
| RXRG | RYBP | SALL4 | SAP30 | SCML2 | SETDB1 | SF1 | SFMBT1 | SFPQ |
| SIN3A | SIRT6 | SIX5 | SMAD1 | SMAD2 | SMAD3 | SMAD4 | SMARCA4 | SMARCB1 |
| SMARCC1 | SMARCC2 | SMC1A | SMC3 | SNAI2 | SNAPC2 | SNAPC4 | SOX17 | SOX2 |
| SOX4 | SOX9 | SP2 | SPDEF | SPIB | SRC | SREBF1 | SREBF2 | SRF |
| SSRP1 | STAT1 | STAT2 | STAT3 | STAT4 | STAT5A | STAT5B | STAT6 | SUMO1 |
| SUMO2 | SUPT5H | TAF1 | TAL1 | TAT | TBL1XR1 | TBP | TCF12 | TCF21 |
| TCF7 | TCF7L1 | TCF7L2 | TEAD1 | TEAD4 | TERF1 | TERF2 | TET2 | TFAP2A |
| TFAP2C | THAP11 | TP53 | TP63 | TP73 | TRIM28 | TTF2 | UBTF | USF1 |
| USF2 | VDR | VEZF1 | WDR5 | WHSC1 | WWTR1 | XBP1 | XRN2 | YAP1 |
| YY1 | ZBTB17 | ZBTB33 |  |  |  |  |  |  |

Supplementary Table 9. The influence of infiltrating cells and risk score on survival by Cox regression

| ID | Hazard_ratio | Low_CI | Ligh_CI | p_value | weight | weight_HR |
| --- | --- | --- | --- | --- | --- | --- |
| riskScore | 5.86 | 3.79 | 9.06 | 0.00 | 14.72 | 486.02 |
| Mast_cells_activated_CIBERSORT | 2.32 | 1.76 | 3.05 | 0.00 | 8.71 | 131.77 |
| Adipocytes_xCell | 4.69 | 2.01 | 10.94 | 0.00 | 3.45 | 368.92 |
| DC_xCell | 0.48 | 0.32 | 0.72 | 0.00 | 3.40 | -51.96 |
| Macrophages_M2_quantiseq | 0.60 | 0.44 | 0.81 | 0.00 | 3.06 | -40.44 |
| CD8+_Tem_xCell | 0.50 | 0.32 | 0.77 | 0.00 | 2.71 | -50.29 |
| iDC_xCell | 0.57 | 0.39 | 0.81 | 0.00 | 2.67 | -43.46 |
| B-cells_xCell | 0.56 | 0.39 | 0.81 | 0.00 | 2.65 | -43.51 |
| T_cells_MCPcounter | 0.42 | 0.24 | 0.74 | 0.00 | 2.59 | -57.58 |
| ImmuneScore_xCell | 0.59 | 0.41 | 0.84 | 0.00 | 2.46 | -41.25 |
| Th1_cells_xCell | 0.62 | 0.45 | 0.86 | 0.00 | 2.41 | -37.95 |
| Mast_cells_resting_CIBERSORT | 0.66 | 0.49 | 0.88 | 0.00 | 2.39 | -34.29 |
| CD8+_Tcm_xCell | 0.58 | 0.39 | 0.84 | 0.00 | 2.36 | -42.47 |
| T_cells_CD8_CIBERSORT | 0.68 | 0.52 | 0.89 | 0.01 | 2.27 | -31.70 |
| CD4+_naive_T-cells_xCell | 0.52 | 0.33 | 0.82 | 0.01 | 2.26 | -48.22 |
| CD8+_T-cells_xCell | 0.60 | 0.41 | 0.87 | 0.01 | 2.15 | -40.47 |
| Memory_B-cells_xCell | 0.47 | 0.27 | 0.82 | 0.01 | 2.12 | -53.00 |
| Cytotoxic_lymphocytes_MCPcounter | 0.58 | 0.39 | 0.87 | 0.01 | 2.08 | -41.66 |
| cDC_xCell | 0.60 | 0.41 | 0.88 | 0.01 | 2.07 | -39.97 |
| Smooth_muscle_xCell | 1.39 | 1.09 | 1.78 | 0.01 | 2.06 | 39.14 |
| MicroenvironmentScore_xCell | 0.65 | 0.47 | 0.90 | 0.01 | 2.00 | -35.22 |
| T_cells_CD8_quantiseq | 0.51 | 0.31 | 0.86 | 0.01 | 1.98 | -48.66 |
| EC_IPS | 0.73 | 0.58 | 0.93 | 0.01 | 1.96 | -26.63 |
| DC_TIMER | 0.67 | 0.49 | 0.92 | 0.01 | 1.92 | -33.26 |
| Macrophages_M0_CIBERSORT | 1.38 | 1.07 | 1.77 | 0.01 | 1.92 | 38.00 |
| Class-switched_memory_B-cells_xCell | 0.70 | 0.53 | 0.93 | 0.01 | 1.87 | -30.01 |
| Macrophages_M1_quantiseq | 1.44 | 1.08 | 1.93 | 0.01 | 1.87 | 44.15 |
| T_cell_CD8_TIMER | 0.59 | 0.39 | 0.91 | 0.02 | 1.80 | -40.85 |
| pro_B-cells_xCell | 0.59 | 0.39 | 0.91 | 0.02 | 1.79 | -40.65 |
| aDC_xCell | 0.74 | 0.58 | 0.95 | 0.02 | 1.72 | -25.81 |
| Basophils_xCell | 0.71 | 0.53 | 0.95 | 0.02 | 1.66 | -29.21 |
| B_lineage_MCPcounter | 0.63 | 0.43 | 0.94 | 0.02 | 1.65 | -36.82 |
| B_cell_TIMER | 0.68 | 0.49 | 0.95 | 0.02 | 1.63 | -32.03 |
| naive_B-cells_xCell | 0.58 | 0.37 | 0.93 | 0.02 | 1.61 | -41.54 |
| ImmuneScore_estimate | 0.77 | 0.61 | 0.97 | 0.03 | 1.56 | -23.32 |
| Bcells_EPIC | 0.61 | 0.40 | 0.95 | 0.03 | 1.53 | -38.58 |
| Endothelial_cells_MCPcounter | 1.38 | 1.03 | 1.84 | 0.03 | 1.49 | 37.54 |
| Preadipocytes_xCell | 1.44 | 1.03 | 2.02 | 0.03 | 1.47 | 44.33 |
| CP_IPS | 1.29 | 1.01 | 1.64 | 0.04 | 1.43 | 28.87 |
| CD8_T_cells_MCPcounter | 0.48 | 0.23 | 0.99 | 0.05 | 1.34 | -51.91 |
| Myeloid_dendritic_cells_MCPcounter | 0.72 | 0.52 | 0.99 | 0.05 | 1.33 | -27.83 |
| CD4+_T-cells_xCell | 0.54 | 0.30 | 0.99 | 0.05 | 1.33 | -45.86 |
| TumorPurity_estimate | 1.28 | 1.00 | 1.65 | 0.05 | 1.28 | 28.35 |
| T_cells_CD4_memory_activated_CIBERSORT | 0.75 | 0.56 | 1.01 | 0.06 | 1.25 | -24.59 |
| pDC_xCell | 0.77 | 0.58 | 1.01 | 0.06 | 1.22 | -23.40 |
| Tregs_quantiseq | 0.76 | 0.56 | 1.03 | 0.07 | 1.13 | -23.90 |
| Neutrophils_quantiseq | 1.31 | 0.97 | 1.79 | 0.08 | 1.09 | 31.48 |
| ESTIMATEScore_estimate | 0.82 | 0.65 | 1.03 | 0.08 | 1.08 | -18.48 |
| CD8_Tcells_EPIC | 0.71 | 0.47 | 1.06 | 0.10 | 1.02 | -29.08 |
| Tregs_xCell | 0.76 | 0.54 | 1.05 | 0.10 | 1.01 | -24.28 |
| CD4+_memory_T-cells_xCell | 0.64 | 0.37 | 1.09 | 0.10 | 1.00 | -36.21 |
| Epithelial_cells_xCell | 1.29 | 0.95 | 1.75 | 0.11 | 0.98 | 28.70 |
| Neutrophils_xCell | 1.33 | 0.93 | 1.91 | 0.12 | 0.91 | 32.89 |
| Plasma_cells_xCell | 0.77 | 0.55 | 1.08 | 0.13 | 0.89 | -22.73 |
| NKcells_EPIC | 0.70 | 0.43 | 1.12 | 0.14 | 0.86 | -30.12 |
| CMP_xCell | 0.63 | 0.34 | 1.18 | 0.15 | 0.82 | -36.68 |
| Macrophages_M1_xCell | 0.81 | 0.60 | 1.09 | 0.16 | 0.79 | -19.39 |
| T_cells_regulatory_(Tregs)_CIBERSORT | 0.83 | 0.63 | 1.08 | 0.17 | 0.78 | -17.30 |
| CAFs_EPIC | 1.26 | 0.91 | 1.76 | 0.17 | 0.78 | 26.35 |
| Fibroblasts_MCPcounter | 1.26 | 0.91 | 1.74 | 0.17 | 0.78 | 25.63 |
| B_cells_quantiseq | 0.80 | 0.59 | 1.10 | 0.17 | 0.78 | -19.84 |
| Macrophages_EPIC | 0.77 | 0.53 | 1.12 | 0.18 | 0.76 | -22.92 |
| Monocytic_lineage_MCPcounter | 0.81 | 0.59 | 1.11 | 0.18 | 0.74 | -19.37 |
| Other_quantiseq | 1.24 | 0.90 | 1.71 | 0.19 | 0.71 | 23.80 |
| Macrophages_M2_xCell | 0.82 | 0.61 | 1.11 | 0.19 | 0.71 | -18.11 |
| Neutrophils_MCPcounter | 1.23 | 0.90 | 1.68 | 0.20 | 0.71 | 22.91 |
| MHC_IPS | 0.86 | 0.68 | 1.08 | 0.20 | 0.71 | -14.30 |
| NK_cells_xCell | 0.77 | 0.52 | 1.16 | 0.21 | 0.67 | -22.73 |
| Skeletal_muscle_xCell | 1.33 | 0.83 | 2.13 | 0.23 | 0.64 | 33.24 |
| Neutrophils_CIBERSORT | 1.29 | 0.84 | 1.98 | 0.24 | 0.61 | 29.03 |
| CD8+_naive_T-cells_xCell | 0.86 | 0.66 | 1.13 | 0.28 | 0.56 | -13.94 |
| Dendritic_cells_resting_CIBERSORT | 0.85 | 0.63 | 1.15 | 0.29 | 0.54 | -14.87 |
| Eosinophils_CIBERSORT | 1.24 | 0.82 | 1.89 | 0.31 | 0.51 | 24.33 |
| B_cells_memory_CIBERSORT | 0.67 | 0.31 | 1.46 | 0.32 | 0.50 | -32.82 |
| SC_IPS | 1.12 | 0.89 | 1.41 | 0.35 | 0.46 | 11.75 |
| Tgd_cells_xCell | 0.88 | 0.67 | 1.16 | 0.37 | 0.43 | -12.02 |
| MEP_xCell | 0.89 | 0.69 | 1.15 | 0.37 | 0.43 | -10.84 |
| T_cells_CD4_quantiseq | 0.79 | 0.46 | 1.34 | 0.38 | 0.42 | -21.16 |
| Myocytes_xCell | 1.14 | 0.85 | 1.51 | 0.39 | 0.41 | 13.51 |
| Monocytes_CIBERSORT | 0.83 | 0.55 | 1.27 | 0.40 | 0.40 | -16.59 |
| Monocytes_quantiseq | 0.76 | 0.39 | 1.46 | 0.41 | 0.39 | -24.27 |
| Neutrophil_TIMER | 0.90 | 0.70 | 1.16 | 0.41 | 0.39 | -10.04 |
| Eosinophils_xCell | 0.85 | 0.58 | 1.26 | 0.42 | 0.37 | -14.66 |
| Macrophages_xCell | 0.88 | 0.64 | 1.21 | 0.43 | 0.37 | -12.19 |
| Osteoblast_xCell | 0.87 | 0.60 | 1.25 | 0.44 | 0.35 | -13.28 |
| Endothelial_cells_xCell | 1.15 | 0.80 | 1.66 | 0.45 | 0.35 | 15.16 |
| T_cell_CD4_TIMER | 0.90 | 0.68 | 1.19 | 0.46 | 0.34 | -10.22 |
| Keratinocytes_xCell | 1.10 | 0.85 | 1.41 | 0.48 | 0.32 | 9.63 |
| StromalScore_estimate | 0.92 | 0.72 | 1.16 | 0.48 | 0.32 | -8.23 |
| MSC_xCell | 1.09 | 0.85 | 1.39 | 0.49 | 0.31 | 9.04 |
| Megakaryocytes_xCell | 1.12 | 0.81 | 1.55 | 0.49 | 0.31 | 12.20 |
| T_cells_CD4_naive_CIBERSORT | 1.19 | 0.72 | 1.97 | 0.49 | 0.31 | 19.29 |
| T_cells_CD4_memory_resting_CIBERSORT | 1.08 | 0.86 | 1.36 | 0.49 | 0.31 | 8.34 |
| NKT_xCell | 0.92 | 0.70 | 1.20 | 0.53 | 0.28 | -8.38 |
| Platelets_xCell | 0.89 | 0.61 | 1.29 | 0.53 | 0.28 | -11.35 |
| Th2_cells_xCell | 0.92 | 0.71 | 1.19 | 0.53 | 0.28 | -7.99 |
| Melanocytes_xCell | 0.91 | 0.68 | 1.22 | 0.53 | 0.27 | -8.82 |
| Sebocytes_xCell | 1.08 | 0.85 | 1.38 | 0.53 | 0.27 | 7.98 |
| Endothelial_EPIC | 1.10 | 0.81 | 1.48 | 0.54 | 0.27 | 9.85 |
| mv_Endothelial_cells_xCell | 0.90 | 0.63 | 1.27 | 0.54 | 0.27 | -10.22 |
| MPP_xCell | 0.76 | 0.32 | 1.83 | 0.55 | 0.26 | -23.68 |
| Erythrocytes_xCell | 1.49 | 0.40 | 5.50 | 0.55 | 0.26 | 49.07 |
| NK_cells_MCPcounter | 0.88 | 0.57 | 1.36 | 0.57 | 0.25 | -11.93 |
| Hepatocytes_xCell | 0.89 | 0.61 | 1.32 | 0.57 | 0.24 | -10.67 |
| T_cells_follicular_helper_CIBERSORT | 0.93 | 0.72 | 1.20 | 0.58 | 0.24 | -7.00 |
| Macrophages_M1_CIBERSORT | 1.07 | 0.84 | 1.38 | 0.58 | 0.24 | 7.35 |
| B_cells_naive_CIBERSORT | 0.93 | 0.70 | 1.23 | 0.60 | 0.22 | -7.38 |
| Macrophages_M2_CIBERSORT | 0.93 | 0.71 | 1.23 | 0.61 | 0.22 | -6.97 |
| NK_cells_quantiseq | 1.09 | 0.79 | 1.50 | 0.62 | 0.21 | 8.58 |
| T_cells_gamma_delta_CIBERSORT | 1.08 | 0.80 | 1.46 | 0.62 | 0.20 | 7.84 |
| otherCells_EPIC | 1.08 | 0.80 | 1.45 | 0.63 | 0.20 | 7.62 |
| Macrophage_TIMER | 1.07 | 0.81 | 1.41 | 0.63 | 0.20 | 6.91 |
| Fibroblasts_xCell | 0.93 | 0.68 | 1.27 | 0.64 | 0.19 | -7.22 |
| Neurons_xCell | 0.91 | 0.62 | 1.34 | 0.64 | 0.19 | -8.67 |
| Pericytes_xCell | 0.92 | 0.66 | 1.30 | 0.64 | 0.19 | -7.73 |
| Monocytes_xCell | 0.93 | 0.69 | 1.26 | 0.65 | 0.19 | -6.63 |
| ly_Endothelial_cells_xCell | 1.08 | 0.73 | 1.60 | 0.68 | 0.17 | 8.47 |
| GMP_xCell | 0.94 | 0.69 | 1.29 | 0.72 | 0.15 | -5.66 |
| Chondrocytes_xCell | 0.95 | 0.69 | 1.29 | 0.73 | 0.14 | -5.30 |
| CLP_xCell | 0.96 | 0.75 | 1.23 | 0.77 | 0.12 | -3.67 |
| CD4+_Tcm_xCell | 0.96 | 0.71 | 1.29 | 0.77 | 0.12 | -4.47 |
| Plasma_cells_CIBERSORT | 0.96 | 0.72 | 1.28 | 0.78 | 0.11 | -3.98 |
| CD4+_Tem_xCell | 0.97 | 0.75 | 1.25 | 0.79 | 0.10 | -3.41 |
| CD4_Tcells_EPIC | 0.97 | 0.76 | 1.23 | 0.79 | 0.10 | -3.19 |
| Mast_cells_xCell | 0.97 | 0.75 | 1.25 | 0.79 | 0.10 | -3.35 |
| StromaScore_xCell | 1.04 | 0.74 | 1.46 | 0.81 | 0.09 | 4.25 |
| Mesangial_cells_xCell | 0.97 | 0.74 | 1.27 | 0.82 | 0.08 | -3.02 |
| IPS_IPS | 1.02 | 0.80 | 1.31 | 0.85 | 0.07 | 2.41 |
| NK_cells_activated_CIBERSORT | 1.03 | 0.77 | 1.36 | 0.86 | 0.07 | 2.66 |
| Dendritic_cells_quantiseq | 0.97 | 0.68 | 1.38 | 0.86 | 0.07 | -3.18 |
| HSC_xCell | 0.98 | 0.73 | 1.31 | 0.88 | 0.06 | -2.30 |
| AZ_IPS | 0.99 | 0.77 | 1.26 | 0.91 | 0.04 | -1.45 |
| Astrocytes_xCell | 0.98 | 0.72 | 1.34 | 0.92 | 0.04 | -1.53 |
| Dendritic_cells_activated_CIBERSORT | 1.01 | 0.74 | 1.37 | 0.95 | 0.02 | 1.00 |
| NK_cells_resting_CIBERSORT | 1.00 | 0.73 | 1.38 | 0.98 | 0.01 | 0.43 |
